# Supplementary material for: Multi-layered Spatial Transcriptomics Identify Secretory Factors Promoting Human Hematopoietic Stem Cell Development
Source: Cell Stem Cell. 2020 Nov 5;27(5):822–839.e8. doi: 10.1016/j.stem.2020.08.004 (PMC7671940; doi:10.1016/j.stem.2020.08.004)
Supplement: Document S1. Figures S1–S7 and Tables S1 and S2 [file mmc1.pdf]

Cell Stem Cell, Volume *27*

Supplemental Information

Multi-layered Spatial Transcriptomics Identify

Secretory Factors Promoting Human

Hematopoietic Stem Cell Development

Edie I. Crosse, Sabrina Gordon-Keylock, Stanislav Rybtsov, Anahi Binagui-Casas, Hannah Felchle, Nneka C. Nnadi, Kristina Kirschner, Tamir Chandra, Sara Tamagno, David J. Webb, Fiona Rossi, Richard A. Anderson, and Alexander Medvinsky

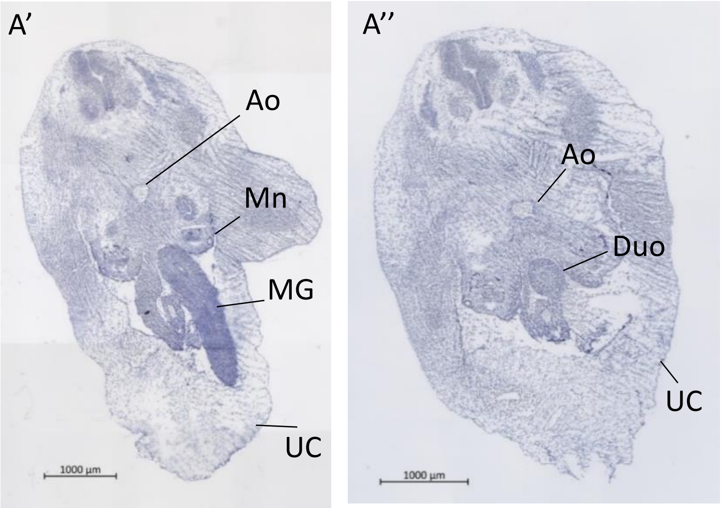

B

	[V] vs [VL]+[DL]+[D]	[VL] vs [V]+[DL]+[D]	[DL] vs [V]+[VL]+[D]	[D] vs [V]+[VL]+[DL]
**LCM-Seq1** No. of genes	+33	+28	+116	+267
	-100	-7	-911	-7
	V_Inner vs D_Inner	V_Mid vs D_Mid	V_Outer vs D_Outer	
**LCM-Seq2** No. of genes	+52	+129	+17	
	-53	-100	-47	

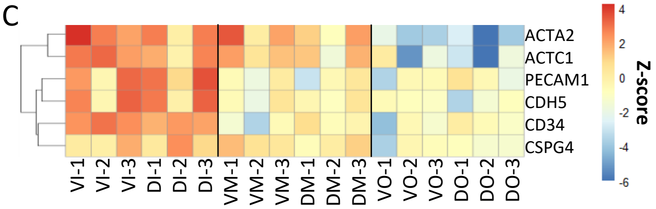

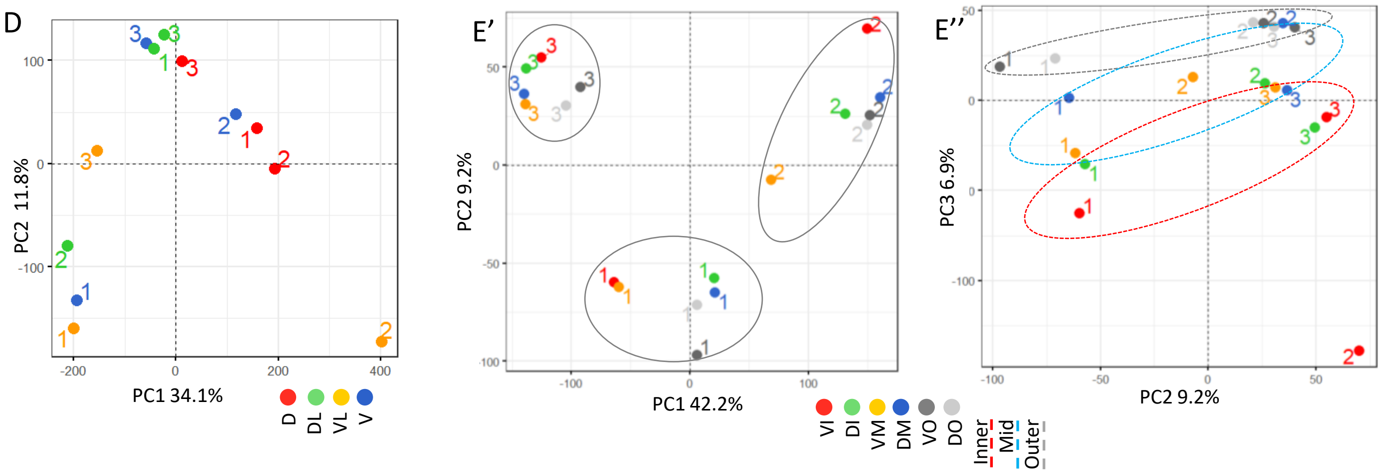

Figure S1, related to Figure 1.

(A) Representative images of anatomical landmarks used for identification of the region to take for LCM-Seq from CS16 transverse sections. A' shows the midgut loop (MG) protruding into the umbilical cord (UC), Ao= Dorsal Aorta, Mn = Mesonephros. A'' shows appearance of the duodenum (Duo) and disappearance of the MG.

(B) Table of differentially expressed genes in LCMSeq1 and LCMSeq2 analyses (+ = upregulated, - = downregulated, p.adj<0.05).

(C) Heatmap of ln-normalised gene expression for key endothelial and mesenchymal markers (LCM-Seq2).

(D) PCA analysis of LCM-Seq1 showing PC1 vs PC2. V=Ventral, VL=Ventro-lateral, DL=Dorsal=lateral, D=Dorsal

(E) PCA analysis of LCM-Seq2 showing PC1 vs PC2 (E') and PC2 vs PC3 (E''). V/DI = Ventral/Dorsal Inner, V/DM = Ventral/Dorsal Mid, V/DO = Ventral/Dorsal Outer, N=3 embryos.

A B C

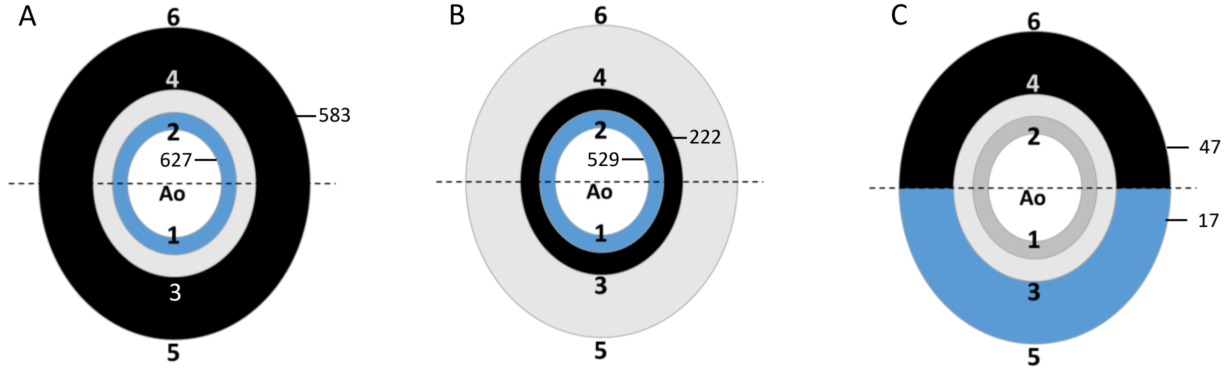

A

	NES	FDR
Reactome Defensins	-1.99	0.02
Spermatogenesis	-1.45	0.05
Nucleotide-like Purinergic Receptors	-1.73	0.07
Smooth Muscle Contraction	2.75	0.00
Integrin B3 Pathway	2.26	0.00
Epithelial Mesenchymal Transition	2.32	0.00
TGF Beta Signaling	2.31	0.00
TNFA Signaling Via Nfkb	2.23	0.00
Androgen Response	2.14	0.00
Hypoxia	2.08	0.00
IL2 Stat5 Signaling	1.94	0.00
MTORC1 Signaling	1.88	0.00
Myogenesis	1.82	0.00
Coagulation	1.76	0.00
Rho GTPases Activate PAKS	2.20	0.00
Wnt Beta Catenin Signaling	1.76	0.00
Unfolded Protein Response	1.78	0.00
PDGFRb Pathway	2.17	0.00
Cell Extracellular Matrix Interactions	2.18	0.00
Response To Elevated Platelet Cytosolic Ca2plus	2.19	0.00
Nectin Pathway	2.17	0.00
Apoptosis	1.72	0.00
Reactive Oxygen Species Pathway	1.71	0.00
Lymph Angiogenesis Pathway	2.12	0.00

B

	NES	FDR
DNA Methylation	-2.13	0.00
Mitosis	-2.00	0.00
Transcription	-1.99	0.01
TGF Beta Signaling	1.71	0.03
Nectin Pathway	2.00	0.05
IL23 Pathway	1.89	0.06
Maturity Onset Diabetes of the Young	1.86	0.06
Integrin A9b1 Pathway	1.84	0.07
Integrin Cell Surface Interactions	1.77	0.07
IL2 Stat5 Signaling	1.57	0.07
Leukocyte Transendothelial Migration	1.77	0.07
Platelet Activation Signaling And Aggregation	1.82	0.08
Gap Junction Assembly	1.75	0.09
Notch4 Intracellular Domain Regulates Transcription	1.73	0.09
Pre Notch Processing In Golgi	1.73	0.09
Integrin3 Pathway	1.71	0.10
Signaling By Ras Mutants	1.65	0.14
Oncogenic Mapk Signaling	1.62	0.16
Response To Elevated Platelet Cytosolic Ca2plus	1.59	0.18
Erbb4 Pathway	1.57	0.19
Basigin Interactions	1.54	0.19
Thrombin Par4 Pathway	1.55	0.19
Il8 Cxcr2 Pathway	1.55	0.19

C

	NES	FDR
E2F Targets	-2.66	0.00
Myc Targets V1	-2.60	0.00
DNA Replication/Mitosis pathways	-2.42	0.00
Oxidative Phosphorylation	-2.15	0.00
Regulation of mRNA Stability By Proteins That Bind AU Rich Elements	-2.28	0.00
Fatty Acid Metabolism	-2.00	0.00
Ribosome	-2.17	0.00
Gluconeogenesis	-2.17	0.00
Mitochondrial Translation	-2.14	0.00
Regulation Of Runx3 Expression And Activity	-2.10	0.00
PDGFRA Pathway	2.15	0.02
EPO Pathway	2.16	0.02
IGF1 Pathway	1.97	0.07
NFAT TF pathway	1.98	0.08
Receptor Type Tyrosine Protein Phosphatases	1.99	0.09
Notch HLH Transcription Pathway	1.93	0.10
PDGF Pathway	1.90	0.11
TPO Pathway	1.89	0.11
Nucleotide Like Purinergic Receptors	1.82	0.13
CD8 TCR Downstream	1.85	0.14
EGF Pathway	1.84	0.14
Renin Angiotensin System	1.84	0.14

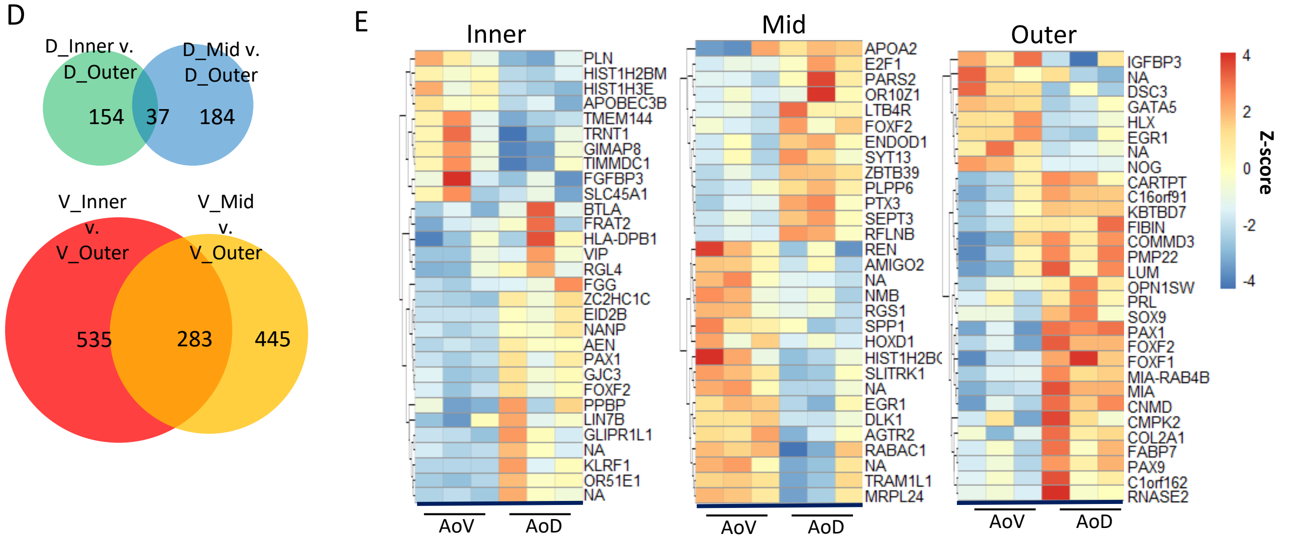

Figure S2, related to Figure 1.

(A) Selected pathways enriched in both V_Inner vs V_Outer and D_Inner vs D_Outer comparisons, FDR<0.25,

(B) Selected pathways enriched in both V_Inner vs V_Mid and D_Inner vs D_Mid comparisons, FDR<0.25. ((A-C) Numbers on schematics show the numbers of genes differentially upregulated in specific sub-domain, p.adj<0.05)

(C) Top differentially enriched pathways ranked by FDR for V_Outer vs D_Outer, FDR<0.25

(D) Number of overlapping pathways (FDR<0.25) between Inner vs Outer and Mid vs Outer for the dorsal (top) and ventral (bottom).

(E) Top 30 genes by significance for ventral vs. dorsal contrast in each concentric layer (p.adj<0.05, NA = Ensembl ID has no assigned gene name).

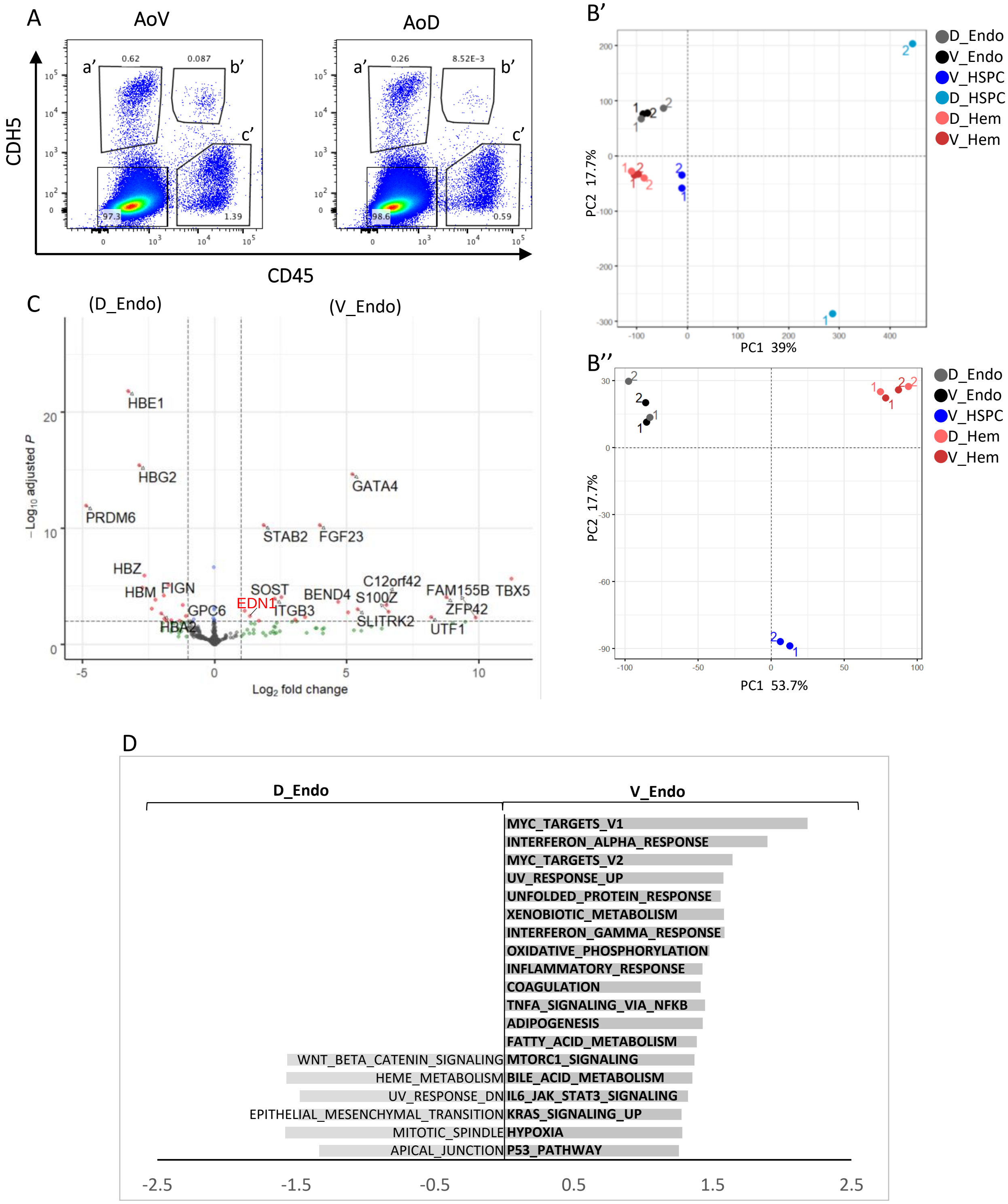
A
AoV
AoD
CDH5
CD45
a'
b'
c'
0.62
0.087
97.3
1.39
0.26
8.52E-3
98.6
0.59
B'
D_Endo
V_Endo
V_HSPC
D_HSPC
D_Hem
V_Hem
PC1 39%
PC2 17.7%
B''
PC1 53.7%
C
(D_Endo)
(V_Endo)
HBE1
HBG2
PRDM6
HBZ
HBM
PIGN
GPC6
HBA2
EDN1
SOST
ITGB3
STAB2
FGF23
GATA4
BEND4
S100Z
C12orf42
SLITRK2
FAM155B
TBX5
ZFP42
UTF1
Log2 fold change
−Log10 adjusted P
D
D_Endo
V_Endo
MYC_TARGETS_V1
INTERFERON_ALPHA_RESPONSE
MYC_TARGETS_V2
UV_RESPONSE_UP
UNFOLDED_PROTEIN_RESPONSE
XENOBIOTIC_METABOLISM
INTERFERON_GAMMA_RESPONSE
OXIDATIVE_PHOSPHORYLATION
INFLAMMATORY_RESPONSE
COAGULATION
TNFA_SIGNALING_VIA_NFKB
ADIPOGENESIS
FATTY_ACID_METABOLISM
WNT_BETA_CATENIN_SIGNALING
MTORC1_SIGNALING
HEME_METABOLISM
BILE_ACID_METABOLISM
UV_RESPONSE_DN
IL6_JAK_STAT3_SIGNALING
EPITHELIAL_MESENCHYMAL_TRANSITION
KRAS_SIGNALING_UP
MITOTIC_SPINDLE
HYPOXIA
APICAL_JUNCTION
P53_PATHWAY
-2.5
-1.5
-0.5
0.5
1.5
2.5

Figure S3, related to Figure 3.

(A) Sorting strategy for hematoendothelial populations. The dorsal aorta (Ao) was manually bisected into ventral (AoV) and dorsal (AoD) and sorted based on VC and CD45 expression that gave V/D_Endo (a'), V/D_HSPC (b'), V/D_Hem (c') populations.

(B) PCA of haematoendothelial populations including D_HSPC population (B') and after exclusion from further consideration (B''). 1 and 2 indicate independent embryos.

(C) Volcano plot of differentially expressed genes between V_Endo and D_Endo populations (positive and negative loading respectively).

(D) All GSEA pathways for V_Endo vs D_Endo populations.

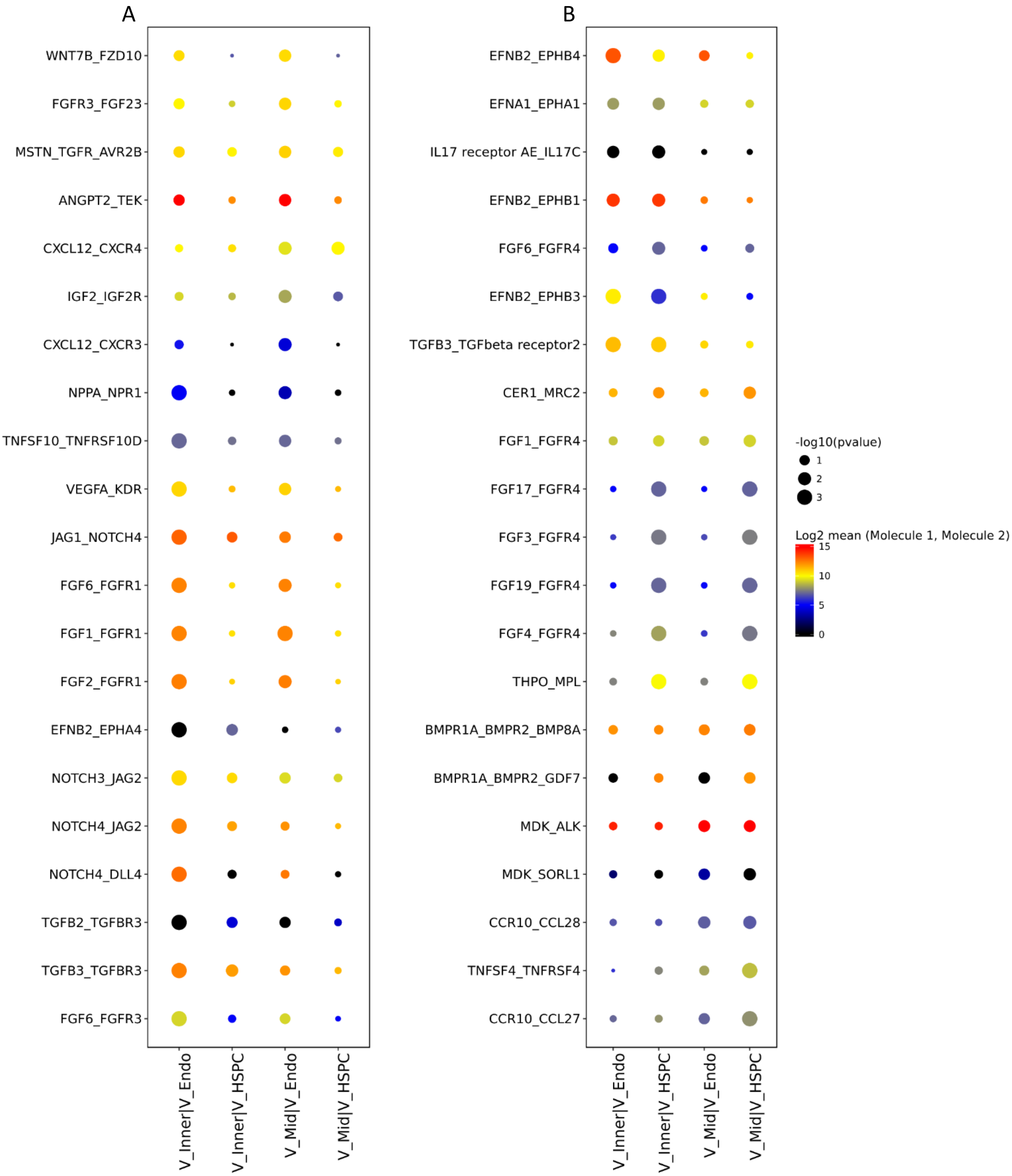

Figure S4, related to Figure 3. CellPhoneDB v2.0 ligand receptor interactions for V_Inner and V_Mid subdomains with V_Endo and V_HSPC populations. Size of spot indicates significance (-log10(pvalue)). Colour indicates expression levels (Log2 mean (Molecule 1 – Molecule 2).

(A) Top interactions by p.value for V_Inner/Mid vs V_Endo.

(B) (B) Top interactions by p.value for V_Inner/Mid vs V_HSPC. p<0.01

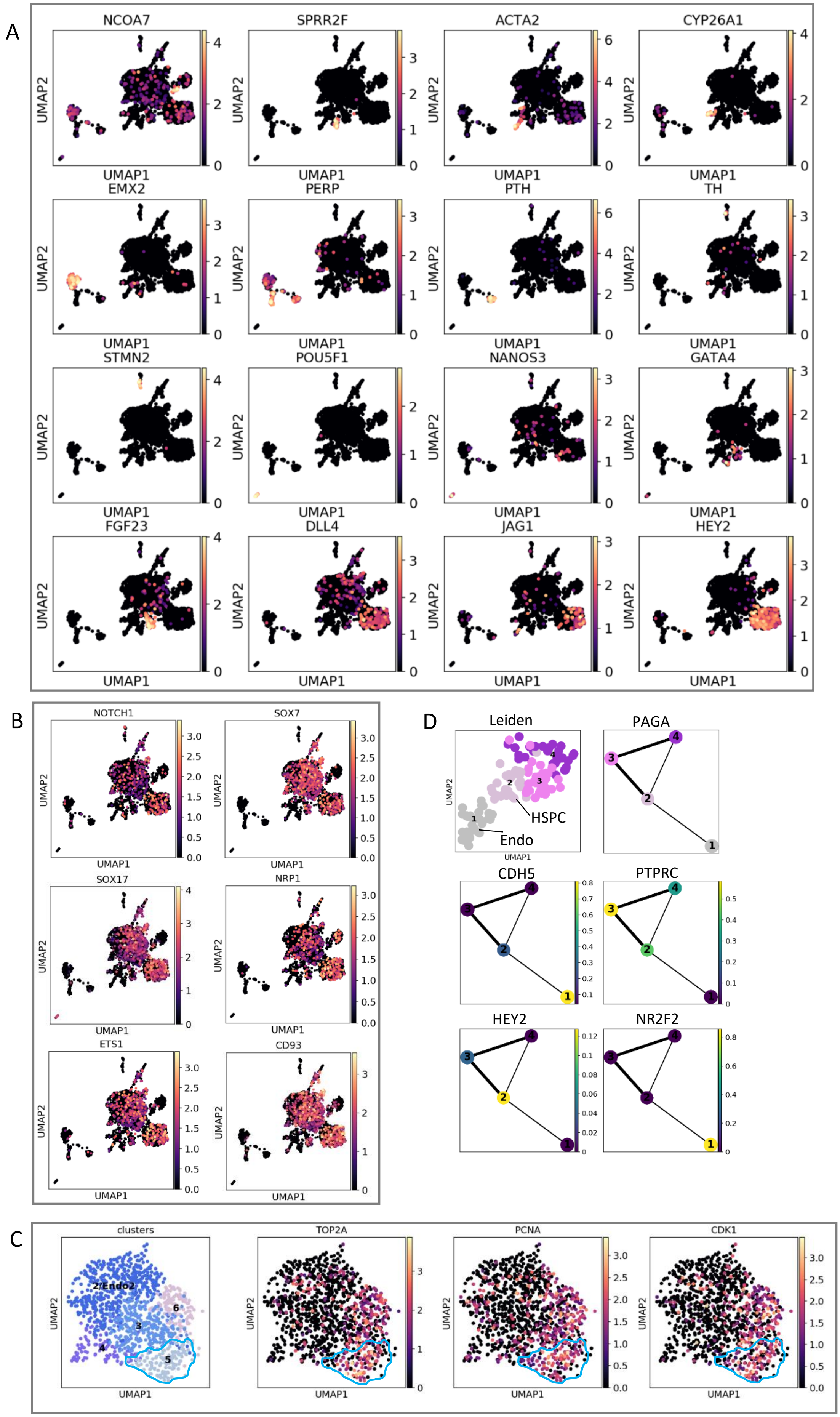
A
NCOA7
SPRR2F
ACTA2
CYP26A1
EMX2
PERP
PTH
TH
STMN2
POU5F1
NANOS3
GATA4
FGF23
DLL4
JAG1
HEY2
UMAP1
UMAP2
B
NOTCH1
SOX7
SOX17
NRP1
ETS1
CD93
D
Leiden
PAGA
HSPC
Endo
CDH5
PTPRC
HEY2
NR2F2
C
clusters
2/Endo2
TOP2A
PCNA
CDK1

Figure S5, related to Figure 4.

(A) Expression mapping of ln-normalised top marker genes to CS16 AoV single cell dataset UMAP.

(B) Expression mapping of additional ln-normalised arterial markers to CS16 AoV single cell dataset UMAP.

(C) Subsetted central endothelial network clusters from AoV dataset with mapped ln-normalised expression of proliferation associated genes. The blue line outlines cluster 5.

(D) Second CS16 single cell dataset of Ao endothelial and HSPCs (N=1). Lineage relationships predicted by PAGA with expression levels of key genes indicated for each cluster.

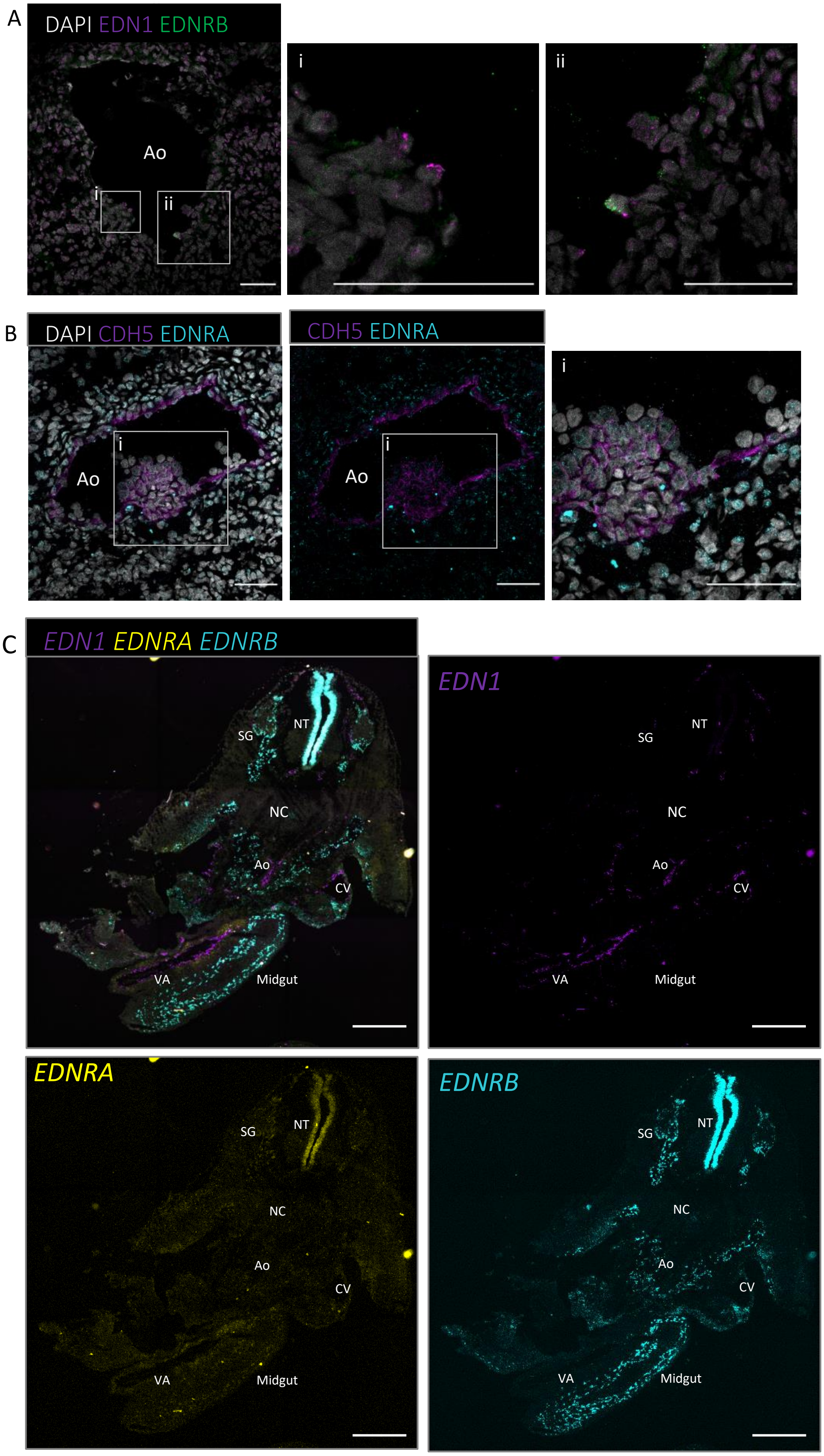
A
DAPI EDN1 EDNRB
Ao
i
ii
B
DAPI CDH5 EDNRA
CDH5 EDNRA
Ao
i
C
EDN1 EDNRA EDNRB
NT
SG
NC
Ao
CV
VA
Midgut
EDN1
EDNRA
EDNRB

Figure S6, related to Figure 6.

(A) EDN1, EDNRB immunostaining (confocal microscopy). Images in i and ii show enlarged area of the boxed region in (A).

(B) CDH5 and EDNRA immunostaining in the dorsal aorta (Ao) and surrounding mesenchyme. Scale bars = 50µm. Image in i show enlarged area of the boxed region in (B).

(A) EDN1, EDNRA and EDNRB mRNA across a CS16 whole tissue section (RNAScope). NT = neural tube, NC = notochord, Ao = dorsal aorta, CV = cardinal vein, VA = vitelline artery. SG = spinal ganglia, scale bars = 500µm.

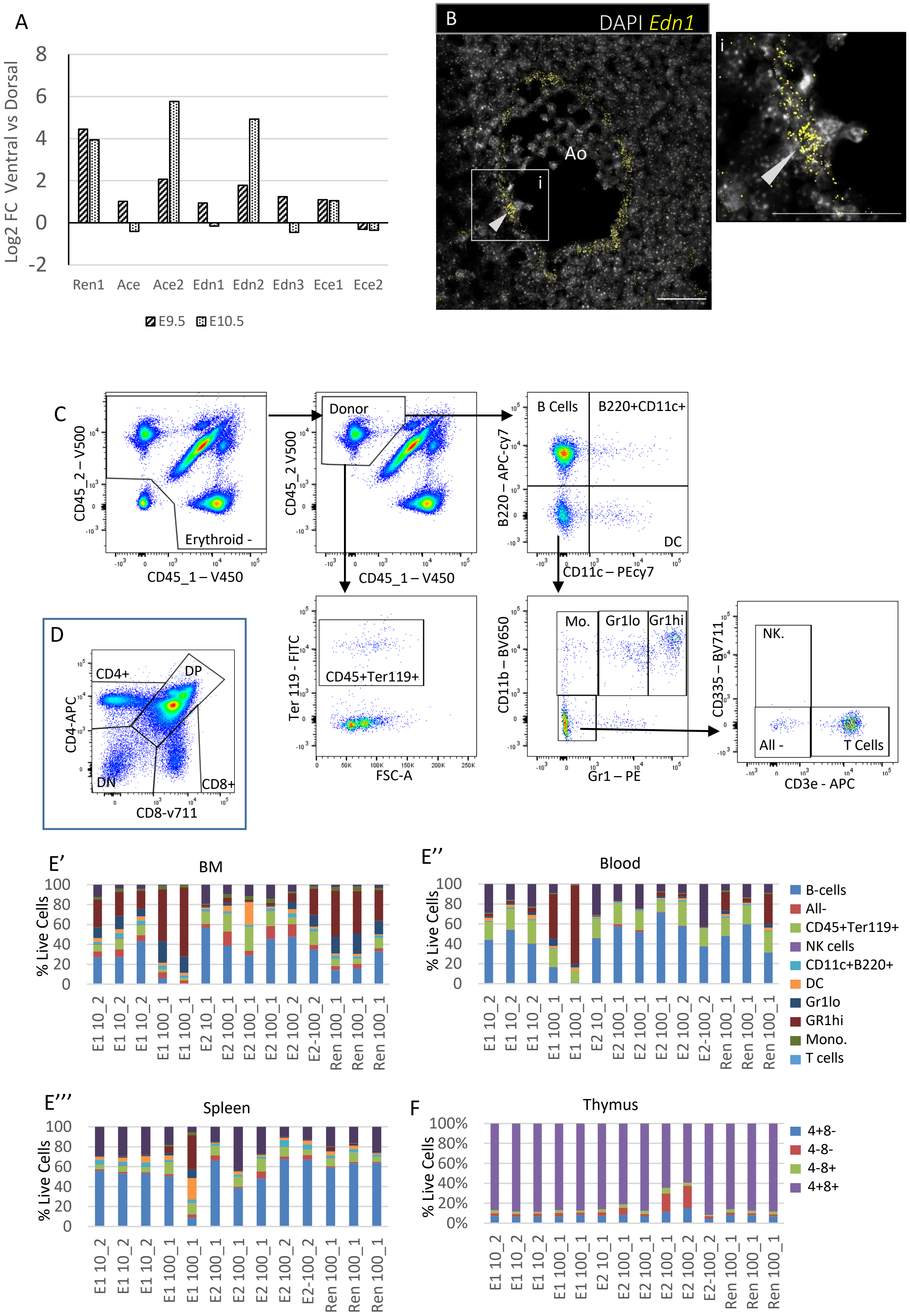
A
Log2 FC Ventral vs Dorsal
8
6
4
2
0
-2
Ren1
Ace
Ace2
Edn1
Edn2
Edn3
Ece1
Ece2
E9.5
E10.5
B
DAPI Edn1
Ao
i
C
CD45_2 – V500
CD45_1 – V450
Erythroid -
Donor
B Cells
B220+CD11c+
B220 – APC-cy7
CD11c – PEcy7
DC
Ter 119 - FITC
CD45+Ter119+
FSC-A
CD11b – BV650
Mo.
Gr1lo
Gr1hi
Gr1 – PE
CD335 – BV711
NK.
All -
T Cells
CD3e - APC
D
CD4-APC
CD4+
DP
DN
CD8+
CD8-v711
E’
BM
% Live Cells
E’’
Blood
B-cells
All-
CD45+Ter119+
NK cells
CD11c+B220+
DC
Gr1lo
GR1hi
Mono.
T cells
E’’’
Spleen
F
Thymus
4+8-
4-8-
4-8+
4+8+
E1 10_2
E1 100_1
E2 10_1
E2 100_1
E2 100_2
E2-100_2
Ren 100_1

Figure S7, related to Figure 7.

(A) Log2 fold change of the mouse AoV vs AoD at E9.5 and E10.5. Dataset from A.McGarvey et all., 2017.

(B) *Edn1* mRNA expression in E10.5 dorsal aorta (Ao). Arrowhead shows *Edn1* hotspot directly below IAHC.

(C) Representative multilineage analysis of harvested recipient's organs from reconstituted recipients shown in Figure 7D;

(D) Representative donor-derived cell contribution into the recipient's thymus of reconstituted recipients.

(E, E', E'', F) Donor-derived cell composition of the bone marrow (BM), blood,spleen and thymus (each population is shown as a percentage of live cells).

Analysis set	Dissection strategy	Subdomain	Shorthand	Description
LCM-Seq1	D-V axis across entire Ao calibre (Figure 1A)	Ventral	[V]	Vessel wall (3-4 cells depth)
		Ventro-lateral	[VL]	
		Dorsal-lateral	[DL]	
		Dorsal	[D]	
LCM-Seq2	Concentric layers radiating from Ao (Figure 2A)	Ventral Inner	V_Inner	Endothelial/Perivascular
		Dorsal Inner	D_Inner	Endothelial/Perivascular
		Ventral Mid	V_Mid	Sub-aortic stroma
		Dorsal Mid	D_Mid	Sub-aortic stroma
		Ventral Outer	V_Outer	Outer stroma
		Dorsal Outer	D_Outer	Outer stroma

Table S1, related to Figures 1 and 2. Nomenclature for sub-domains microdissected for LCM-Seq1 and LCM-Seq2 analyses.

Analysis set	Dorsal/Ventral	Population	Shorthand	Description
Sorted haematoendothelial populations from AoV and AoD (RNAseq)	Ventral	VC+CD45-	V_Endo	Endothelial
	Ventral	VC+CD45+	V_HSPC	Haematopoietic Stem Progenitor Cells
	Ventral	VC-CD45+	V_Hem	Mature haematopoietic
	Dorsal	VC+CD45-	D_Endo	Endothelial
	Dorsal	VC+CD45+	D_HSPC	Haematopoietic Stem Progenitor Cells
	Dorsal	VC-CD45+	D_Hem	Mature haematopoietic

Table S2, related to Figure 3. Nomenclature for the sorted HE populations dataset.
