# Supplementary material for: Multi-layered Spatial Transcriptomics Identify Secretory Factors Promoting Human Hematopoietic Stem Cell Development
Source: Cell Stem Cell. 2020 Nov 5;27(5):822–839.e8. doi: 10.1016/j.stem.2020.08.004 (PMC7671940; doi:10.1016/j.stem.2020.08.004)
Supplement: Document S2. Article plus Supplemental Information [file mmc5.pdf]

Cell Stem Cell

Resource

Multi-layered Spatial Transcriptomics Identify Secretory Factors Promoting Human Hematopoietic Stem Cell Development

Graphical Abstract

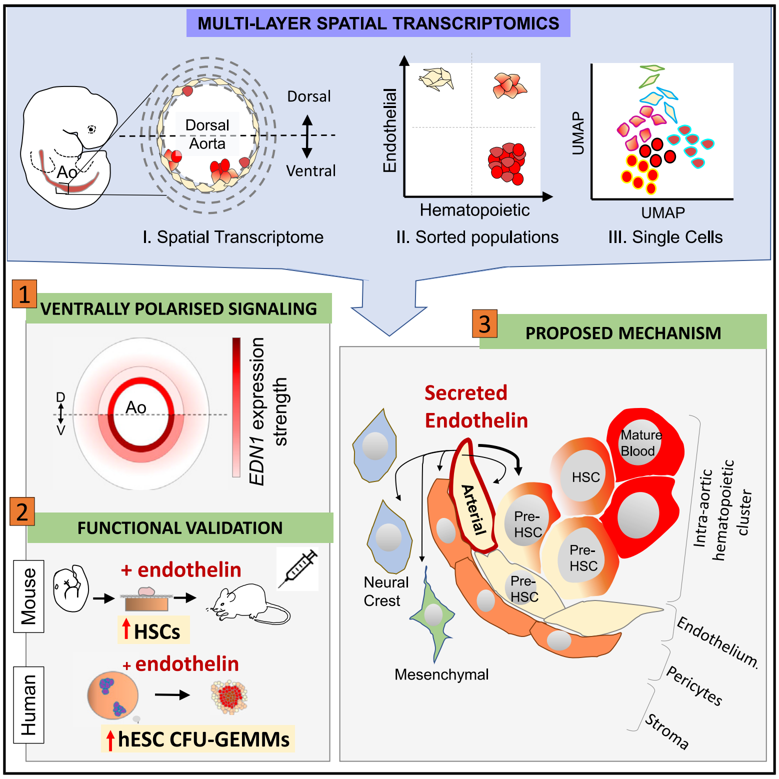

Highlights

- Spatial transcriptome profiling of the human HSC developmental niche
- Characterization of an HSC precursor population at single-cell resolution
- Cardiac EGF pathway is ventrally enriched next to developing IAHCs/HSCs
- Ventrally secreted endothelin promotes development of HSCs

Authors

Edie I. Crosse, Sabrina Gordon-Keylock, Stanislav Rybtsov, ..., Fiona Rossi, Richard A. Anderson, Alexander Medvinsky

Correspondence

a.medvinsky@ed.ac.uk

In Brief

Crosse et al. combined spatial, population, and single-cell transcriptomics to interrogate signaling in the human hematopoietic stem cell (HSC) embryonic niche. They identified secreted proteins proximal to the site of HSC emergence, including endothelins, which, they demonstrated, can promote hematopoiesis in mouse and human.

Crosse et al., 2020, Cell Stem Cell 27, 822–839
November 5, 2020 © 2020 Elsevier Inc.
https://doi.org/10.1016/j.stem.2020.08.004

CellPress

CellPress

Cell Stem Cell

Resource

Multi-layered Spatial Transcriptomics Identify Secretory Factors Promoting Human Hematopoietic Stem Cell Development

Edie I. Crosse,[1] Sabrina Gordon-Keylock,[1] Stanislav Rybtsov,[1] Anahi Binagui-Casas,[1] Hannah Felchle,[1] Nneka C. Nnadi,[1] Kristina Kirschner,[2] Tamir Chandra,[3] Sara Tamagno,[1] David J. Webb,[4] Fiona Rossi,[1] Richard A. Anderson,[5] and Alexander Medvinsky[1,6,*]

[1]MRC Centre for Regenerative Medicine, University of Edinburgh, Edinburgh EH16 4UU, UK
[2]Institute of Cancer Sciences, University of Glasgow, Bearsden G61 1QH, UK
[3]MRC Human Genetics Unit, University of Edinburgh, Edinburgh EH4 2XU, UK
[4]BHF Centre for Cardiovascular Science, University of Edinburgh, Edinburgh EH16 4TJ, UK
[5]MRC Centre for Reproductive Health, University of Edinburgh, Edinburgh EH16 4TJ UK
[6]Lead Contact
*Correspondence: a.medvinsky@ed.ac.uk
https://doi.org/10.1016/j.stem.2020.08.004

SUMMARY

Hematopoietic stem cells (HSCs) first emerge in the embryonic aorta-gonad-mesonephros (AGM) region. Studies of model organisms defined intersecting signaling pathways that converge to promote HSC emergence predominantly in the ventral domain of the dorsal aorta. Much less is known about mechanisms driving HSC development in humans. Here, to identify secreted signals underlying human HSC development, we combined spatial transcriptomics analysis of dorsoventral polarized signaling in the aorta with gene expression profiling of sorted cell populations and single cells. Our analysis revealed a subset of aortic endothelial cells with a downregulated arterial signature and a predicted lineage relationship with the emerging HSC/progenitor population. Analysis of the ventrally polarized molecular landscape identified endothelin 1 as an important secreted regulator of human HSC development. The obtained gene expression datasets will inform future studies on mechanisms of HSC development *in vivo* and on generation of clinically relevant HSCs *in vitro*.

INTRODUCTION

The first definitive hematopoietic stem cells (HSCs) that give rise to the adult hematopoietic system in mouse and human emerge in the embryonic aorta-gonad-mesonephros (AGM) region (Medvinsky and Dzierzak, 1996; Ivanovs et al., 2011; de Bruijn et al., 2000). Generation of HSCs and progenitor cells is manifested as formation of intra-aortic hematopoietic cell clusters (IAHCs), conserved across vertebrates (Garcia-Porrero et al., 1995; Medvinsky et al., 1996; Tavian et al., 1996; Tavian et al., 1999; Jaffredo et al., 1998; Ciau-Uitz et al., 2000; Yokomizo and Dzierzak, 2010). IAHCs/HSCs are formed through transition of the aortic endothelium toward the hematopoietic fate in a process termed endothelial-to-hematopoietic transition (EHT) (Jaffredo et al., 1998; Zovein et al., 2008; Medvinsky et al., 2011; Batsivari et al., 2017; Chen et al., 2009; Taoudi et al., 2008; Kissa and Herbomel, 2010; Bertrand et al., 2010). In the mouse, HSC maturation is accompanied by sequential upregulation of the hematopoietic markers CD41, CD43, and CD45 and critical transcription factors (TFs) such as Runx1, Gata2, and Gfi1 (Mikkola et al., 2003; Taoudi et al., 2005; Nottingham et al., 2007; Boisset et al., 2010; Rybtsov et al., 2011; de Pater et al., 2013; Rybtsov et al., 2014; Thambyrajah et al., 2016; Baron et al., 2018).

In the human embryo, definitive HSCs emerge between Carnegie stage 14 (CS14) and CS17 (postovulatory days 32–41) (Ivanovs et al., 2011), which overlaps with the time of appearance of IAHCs (Tavian et al., 1996, 1999). To date, human EHT cannot be tracked directly *in vivo*. However, *in vitro* modeling using human embryonic stem cells (hESCs) revealed transition through endothelial intermediates toward the hematopoietic fate (Slukvin, 2013; Ayllón et al., 2015; Ditadi et al., 2015; Rönn et al., 2015; Ditadi et al., 2017). Recent single-cell transcriptomics analysis at earlier CS12–CS14 (postovulatory days 27–32) also indicated a lineage relationship between human endothelium and hematopoietic stem and progenitor cells (HSPCs) (Zeng et al., 2019).

IAHCs/HSCs emerge predominantly in the ventral domain of the dorsal aorta (AoV), which has been identified as the functional HSC niche in mouse and human (Peeters et al., 2009; Taoudi and Medvinsky, 2007; Ivanovs et al., 2014; Souilhol et al., 2016a; McGarvey et al., 2017; Ciau-Uitz et al., 2016). Subsequent analysis of ventrally polarized secreted factors revealed their important role in mouse HSC development (Souilhol et al., 2016a; McGarvey et al., 2017). Although analysis of vertebrate models shed light on early hematopoietic development, the mechanisms underpinning this process in human are much less clear (Easterbrook et al., 2019).

 © 2020 Elsevier Inc.

Check for updates

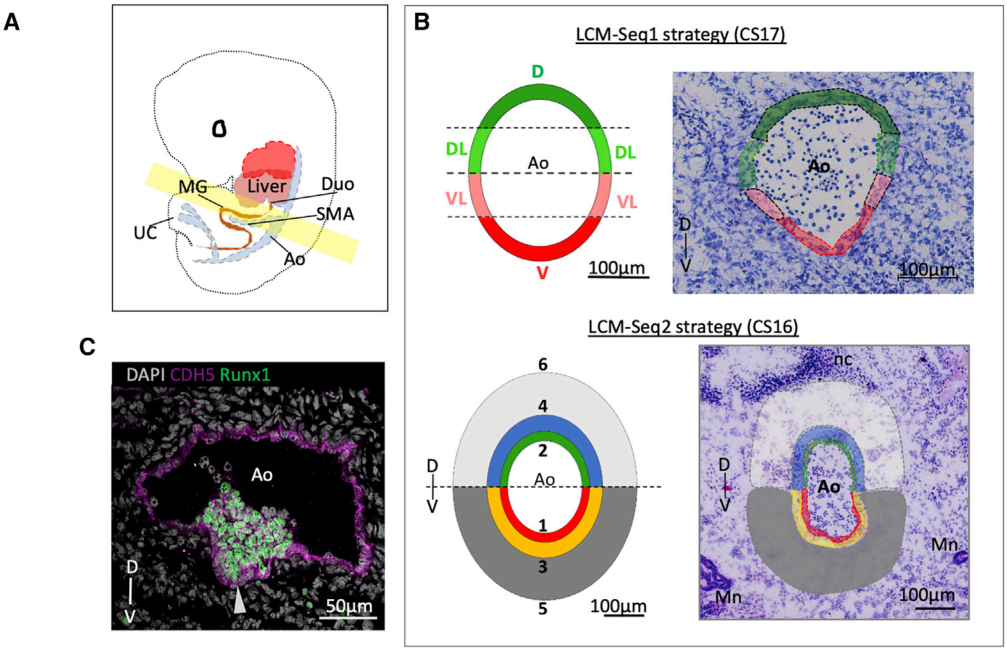

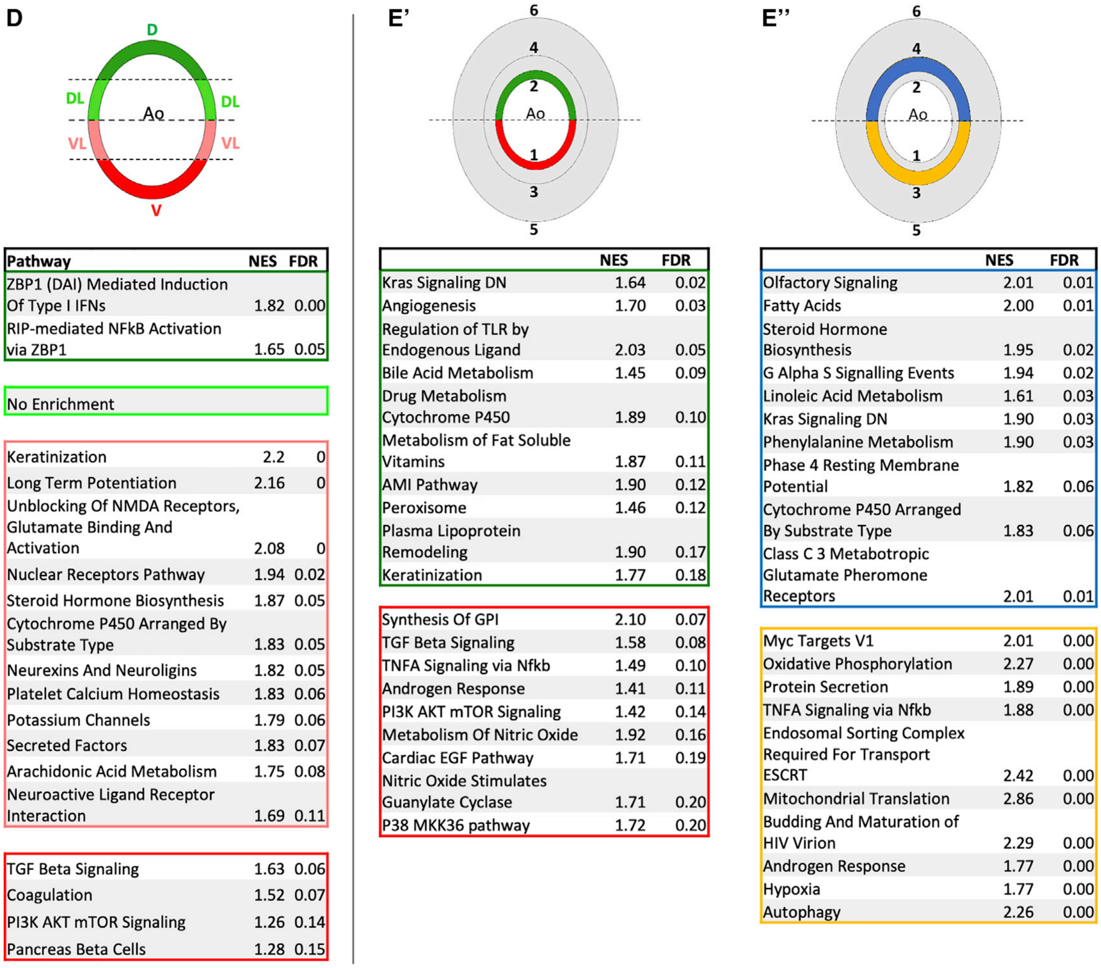

D

Pathway	NES	FDR
ZBP1 (DAI) Mediated Induction Of Type I IFNs	1.82	0.00
RIP-mediated NFkB Activation via ZBP1	1.65	0.05

No Enrichment		

Keratinization	2.2	0
Long Term Potentiation	2.16	0
Unblocking Of NMDA Receptors, Glutamate Binding And Activation	2.08	0
Nuclear Receptors Pathway	1.94	0.02
Steroid Hormone Biosynthesis	1.87	0.05
Cytochrome P450 Arranged By Substrate Type	1.83	0.05
Neurexins And Neuroligins	1.82	0.05
Platelet Calcium Homeostasis	1.83	0.06
Potassium Channels	1.79	0.06
Secreted Factors	1.83	0.07
Arachidonic Acid Metabolism	1.75	0.08
Neuroactive Ligand Receptor Interaction	1.69	0.11

TGF Beta Signaling	1.63	0.06
Coagulation	1.52	0.07
PI3K AKT mTOR Signaling	1.26	0.14
Pancreas Beta Cells	1.28	0.15

E'

	NES	FDR
Kras Signaling DN	1.64	0.02
Angiogenesis	1.70	0.03
Regulation of TLR by Endogenous Ligand	2.03	0.05
Bile Acid Metabolism	1.45	0.09
Drug Metabolism Cytochrome P450	1.89	0.10
Metabolism of Fat Soluble Vitamins	1.87	0.11
AMI Pathway	1.90	0.12
Peroxisome	1.46	0.12
Plasma Lipoprotein Remodeling	1.90	0.17
Keratinization	1.77	0.18

Synthesis Of GPI	2.10	0.07
TGF Beta Signaling	1.58	0.08
TNFA Signaling via Nfkb	1.49	0.10
Androgen Response	1.41	0.11
PI3K AKT mTOR Signaling	1.42	0.14
Metabolism Of Nitric Oxide	1.92	0.16
Cardiac EGF Pathway	1.71	0.19
Nitric Oxide Stimulates Guanylate Cyclase	1.71	0.20
P38 MKK36 pathway	1.72	0.20

E''

	NES	FDR
Olfactory Signaling	2.01	0.01
Fatty Acids	2.00	0.01
Steroid Hormone Biosynthesis	1.95	0.02
G Alpha S Signalling Events	1.94	0.02
Linoleic Acid Metabolism	1.61	0.03
Kras Signaling DN	1.90	0.03
Phenylalanine Metabolism	1.90	0.03
Phase 4 Resting Membrane Potential	1.82	0.06
Cytochrome P450 Arranged By Substrate Type	1.83	0.06
Class C 3 Metabotropic Glutamate Pheromone Receptors	2.01	0.01

Myc Targets V1	2.01	0.00
Oxidative Phosphorylation	2.27	0.00
Protein Secretion	1.89	0.00
TNFA Signaling via Nfkb	1.88	0.00
Endosomal Sorting Complex Required For Transport ESCRT	2.42	0.00
Mitochondrial Translation	2.86	0.00
Budding And Maturation of HIV Virion	2.29	0.00
Androgen Response	1.77	0.00
Hypoxia	1.77	0.00
Autophagy	2.26	0.00

(legend on next page)

Here we aimed to spatially characterize the developing HSC niche (hereafter referred to as "niche") and identify secreted factors involved in early human HSC development. Using laser capture microdissection coupled with RNA sequencing (LCM-seq), we investigated dorsal-ventral (D-V) molecular differences across the dorsal aorta (Ao) with a focus on cell layers close to IAHC formation. We also studied gene expression dynamics across EHT within the aortic niche at the population and single-cell levels and revealed a close link of emerging HSPCs with a specific endothelial cell subset in which the arterial signature was markedly downregulated.

Our analyses identified numerous ventrally polarized signaling pathways, including those with a well-documented role in HSPC development. We focused on one of them, cardiac epidermal growth factor (EGF), not implicated previously in HSC development and found that its major regulator, endothelin 1, enhances the multipotency of human ES cell-derived hematopoietic progenitors, whereas in the mouse, the highly similar isoform endothelin 2 is a strong pro-HSC maturation factor. Additionally, the gene expression database generated here can provide deep insights into normal and potentially congenital pathological processes related to blood development and potentially inform strategies to gain better control of *ex vivo* HSC manipulations.

RESULTS

Mapping D-V Signaling Polarization in the HSC Developmental Niche

To reveal D-V polarization within the human Ao, we performed spatially defined microdissection using LCM. Transverse cryosections of CS16–CS17 embryos were taken between the liver caudal border (rostral limit) and the midgut loop (caudal limit) (Figures 1A and S1A), where IAHCs/HSCs predominantly emerge (Tavian et al., 1996; Tavian et al., 1999; Easterbrook et al., 2019).

Two types of microdissections were performed. In the first series (LCM-seq1), the Ao walls (3–4 cell layers thick, CS17 embryos, n= 3), were dissected into ventral ([V]), ventrolateral ([VL]), dorsolateral ([DL]) and dorsal ([D]) subdomains (Figure 1B; Table S1). In the second series (LCM-seq2), three concentric cell layers were microdissected, radiating away from the Ao lumen (CS16 embryos, n = 3), referred to as inner (luminal endothelial and perivascular cells), mid (subendothelial stromal cells), and outer (more distal stromal cells) (Figure 1B). Each layer was subdissected along the midline into dorsal and ventral domains (see nomenclature in Table S1) and used for RNA sequencing (RNA-seq). In both experiments, sister sections were immunostained for CDH5 (VE-Cadherin) and Runx1 to validate the presence of CDH5+Runx1+ IAHCs (Figure 1C). Thus, D-V polarization was assessed at two levels: across the entire Ao diameter (LCM-seq1) and across the entire Ao wall depth (LCM-seq2), enabling prioritization of key genes.

Molecular Signaling Is Polarized along the D-V Axis

Gene set enrichment analysis (GSEA) of the LCM-seq1 dataset revealed an exceptionally high pathway number enriched in [VL] (250) compared with [V] (4), [DL] (4), and [D] (5) (Data S1). [VL] was enriched for neuro-specific pathways (e.g., long-term potentiation, unblocking of NMDA receptors, glutamate binding and activation, neurexins and neuroglins, and neuroactive ligand receptor interaction; (Figure 1D), in keeping with the proximity to ventrolaterally localized sympathetic ganglia, an important component of the HSC niche (Fitch et al., 2012). Notably, [V] showed enrichment of transforming growth factor β (TGF-β) signaling, observed previously in the mouse AoV (McGarvey et al., 2017).

The second dataset, LCM-seq2, revealed D-V differences across the Ao wall and deeper stromal layers (Figures 1E and S2C). As expected, endothelial (*PECAM1*, *CDH5*, and *CD34)* and perivascular markers (*ACTA2*, *ACTC1*, and *CSPG4* [NG2]) were highly expressed in the inner layer, followed by a decrease in endothelial (but sustained perivascular markers) in the mid layer and a further decrease of these markers in the outer layer (Figure S1C). Principal-component analysis (PCA) clustered samples by biological variant along principal component 1 (PC1) and PC2, highlighting biological variability between human samples (biological variability is less marked for LCM-seq1) (Figures S1D and S1E). By PC3, stratification (and distinct molecular identities) of the inner, mid, and outer layers became evident (Figure S1E''). For subsequent differential expression analysis, biological variability was corrected (STAR Methods).

GSEA identified inner layer-specific pathways involved in blood vessel function, maintenance, and angiogenesis (including smooth muscle contraction, integrin β3, and TGF-β signaling) (Figures S2A and S2B). Notably, ventrally enriched pathways in all three concentric layers included tumor necrosis factor alpha (TNF-α) signaling via nuclear factor κB, phosphatidylinositol 3-kinase (PI3K) AKT MTOR signaling, and metabolism of nitric oxide, associated with HSC development (Figures 1E and S2C; Data S1) (Adamo et al., 2009; North et al., 2009; Wang et al., 2011; Espín-Palazón et al., 2014; Zhou et al., 2016). Additionally, the V_Outer compared with the D_Outer layer was enriched for some known hematopoietic factors, including EPO and TPO (Figure S2C). Thus, all AoV cell layers can potentially contribute to ventrally polarized development of the adult hematopoietic

Figure 1. Signaling Heterogeneity along the D-V Axis of the Ao

(A) Schematic of a CS16–CS17 embryo. The region highlighted in yellow is taken for LCM-seq; anatomical landmarks of rostral and caudal limits are shown in Figure S1. Ao, dorsal aorta; Duo, duodenum; SMA, superior mesenteric artery; MG, midgut loop; UC, umbilical cord.

(B) Strategy of LCM-mediated subdissection (left) superimposed onto an example Ao transverse section (right) for LCM-seq1 (top) and LCM-seq2 (bottom). V, ventral; VL, ventrolateral; DL, dorsal-lateral; D, dorsal; 1, V_Inner; 2, D_Inner; 3, V_Mid; 4, D_Mid; 5, V_Outer; 6, D_Outer; Mn, mesonephros; nc, notochord.

(C) Sister section stained for CDH5 and Runx1 using antibody staining. The arrowhead indicates an IAHC adhering to the V endothelium.

(B and C) The D-V axis is indicated.

(D and E) Top pathways by false discovery rate (FDR) for LCM-seq domains highlighted in the schematic. The color of the table corresponds with the subdomain indicated in the schematic above. FDR < 0.25.

(D) LCM-seq1: D, DL, VL, and V (each versus the remaining 3 domains).

(E) LCM-seq2: V_Inner (red) versus D_Inner (green) (E') and V_Mid (yellow) versus D_Mid (blue) (E'').

Numbers of significant differentially expressed genes for each contrast are shown in Figure S1B.

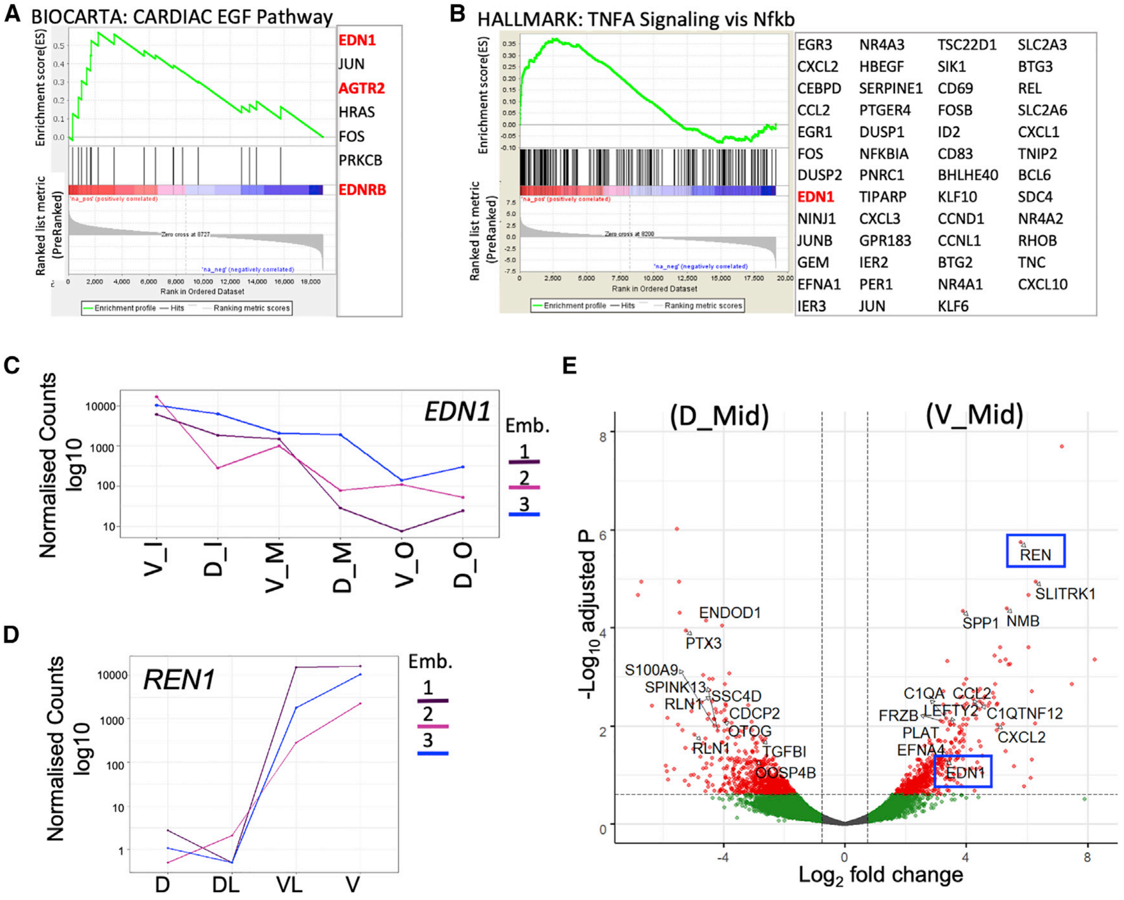

Figure 2. Spatial Molecular Polarization across the Ao Wall and Surrounding Stroma (LCM-Seq2)

(A and B) Enrichment plots for V_Inner versus D_Inner "Cardiac EGF" pathway (A) and V_Mid versus D_Mid TNF-α signaling via NF-κB (B). Contributing genes are shown. Endothelin- and angiotensin-related genes are highlighted in red.

(C) LCM-seq2: the expression levels of EDN1 decrease with distance from the Ao lumen (LCM-seq2) (adjusted p value [p.adj] = 2×10^{-7}; N = 3, indicated by different colors).

(D) LCM-seq1: the expression levels of REN increase significantly from D and DL to V and VL.

(E) Volcano plot of significant genes, V_Mid versus D_Mid, with secreted factors marked (positive loading versus negative loading, respectively). *REN* (most significant secreted factor (p.adj = 1.8×10^{-6}) and *EDN1* are marked by blue boxes.

system. Furthermore, despite polarization across the AoV depth, a remarkable integrity between the V_Inner and V_Mid concentric layers (283 pathways shared) (Figure S2D), suggested strong functional coherence within the developing HSC niche. The same AoD concentric layers shared only 37 pathways, indicating functional heterogeneity.

Renin and Endothelin 1 Are Ventrally Enriched Secreted Factors

AoV secreted factors are potential key HSC niche signaling molecules. Intriguingly, V_Inner, intimately related to IAHC formation, was enriched for the cardiac EGF pathway currently not associated with HSC development (Figures 1E' and 2A). This pathway is stimulated by the secreted factor endothelin 1 (EDN1) and the renin-angiotensin pathway ligand angiotensin II (causing cardiac hypertrophy in response to high blood pressure) (Shah and Catt, 2003). Indeed, *EDN1*, *EDNRB* (endothelin receptor B), and *AGTR2* (angiotensin receptor 2) contributed to V_Inner (versus D_Inner) and V_Mid (versus D_Mid) enrichment (Figure 2A). *EDN1* also contributed to ventral enrichment of HSC-related TNF-α signaling via NF-κB in V_Inner and V_Mid (Figure 2B). *EDN1* was the most significant secreted factor that showed a monotonic expression gradient, increasing toward the Ao lumen (Figure 2C). Notably, among three genes (LCM-seq1) with monotonic expression growing from [D]/[DL] toward [V]/[VL] was *REN* (Figure 2D), which acts upstream of angiotensin II and angiotensin-converting enzyme (*ACE*), a suggested HSC marker in the human AGM (Jokubaitis et al., 2008; Sinka et al., 2012). Additionally, *REN* was the most significantly upregulated secreted factor in V_Mid versus D_Mid (Figures 2E and S2E). The entire renin-angiotensin pathway was also enriched in V_Outer versus D_Outer (Figure S2C). Renin and endothelin 1, both encoding blood pressure regulators, have a reciprocal and interactive relationship (Beierwaltes and Carretero, 1992; Lin et al., 1993; Ackermann et al., 1995; Rossi et al., 1999). Their coincident ventral enrichment is suggestive of a potential role in the hematopoietic niche.

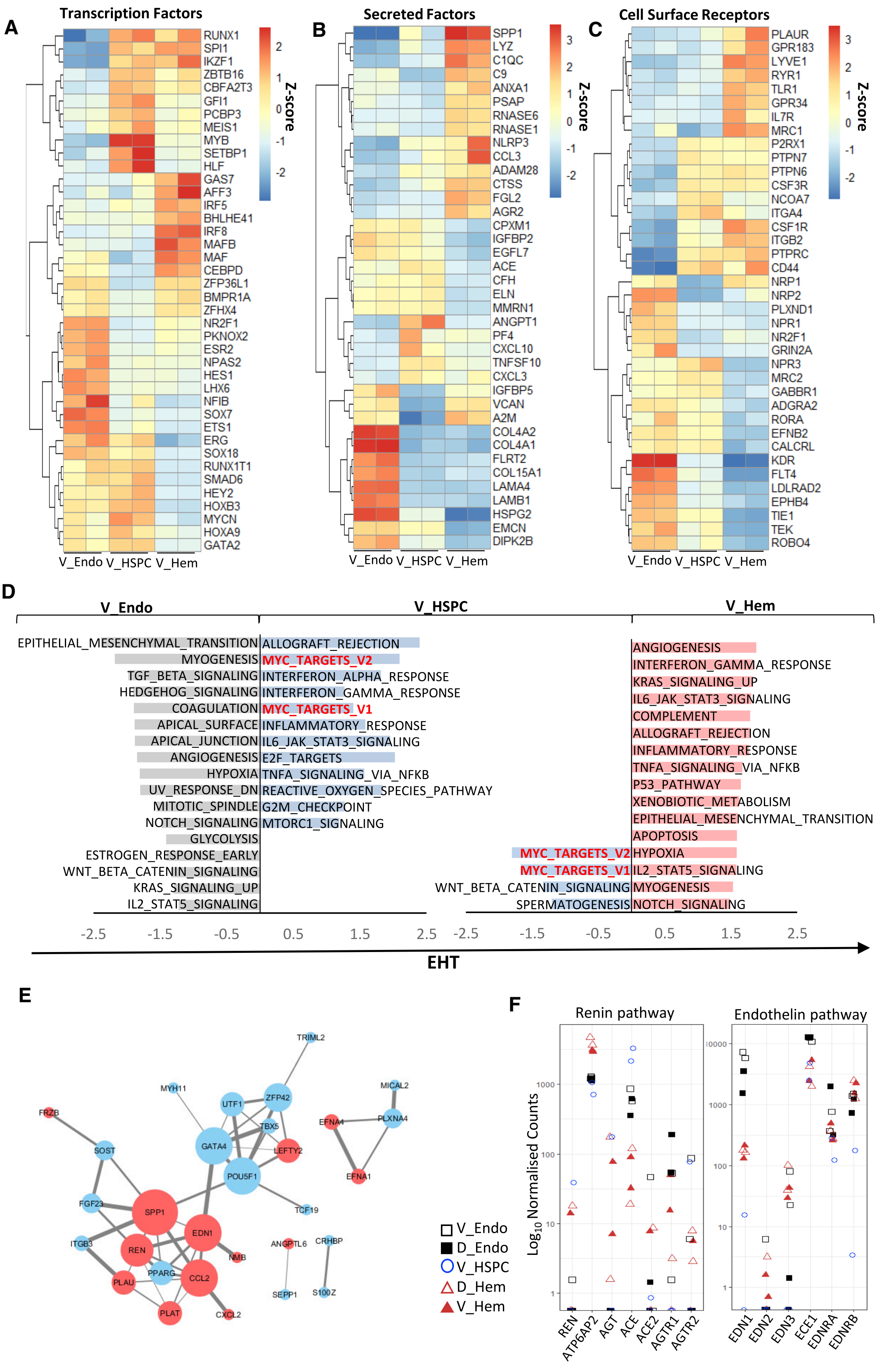

(legend on next page)

Gene Expression Dynamics across the EHT

Profiling of Three Major EHT Populations

To map gene expression during EHT, the Ao from two embryos (CS15–CS16) were manually bisected, and three cell populations from the AoV and dorsal (AoD) domains were sorted and profiled by RNA-seq: endothelial CDH5+CD45− cells (V_Endo and D_Endo), hematopoietic stem/progenitor CDH5+CD45+ cells (V_HSPCs and D_HSPC), and mature hematopoietic CDH5−CD45+ cells (V_Hem and D_Hem) (Figure S3A; Table S2). PCA showed clustering according to cell population identity, except for scattered D_HSPCs (Figure S3B'), which might reflect an unstable nature of hematopoiesis in the AoD (these samples were excluded from further analysis). As expected, V_HSPC, in accordance with its intermediate position along the EHT axis, falls between the V/D_Hem and V/D_Endo populations along PC1 (Figure S3B'').

D-V endothelial polarization (Figure S3C) was evident from differential gene expression in the V_Endo (36) and D_Endo (58) populations (Data S2). GSEA again showed TNF-α signaling via NF-κB and MTORC signaling enrichment in the V_Endo versus D_Endo population as in all ventral layers detected by LCM-seq1 (Figures S3D, 1E, and S2C; Data S1). Top significant genes in V_Endo were *GATA4*, *STAB2*, *FGF23*, and *TBX5* (not detected by LCM-seq, likely because of confounding signals from non-endothelial, perivascular cell types) (Figure S3C). Thus, analysis of purified cell populations complemented spatial LCM-seq and revealed additional D-V molecular polarization. Notably, *EDN1* was also enriched in the sorted V_Endo compared with D_Endo.

Molecular Signaling across EHT

We then profiled differentially expressed TFs, secreted factors, and cell surface proteins within V_Endo, V_HSPC, and more mature V_Hem populations (Figures 3A–3C; Data S2) and observed 5 main patterns. (1) 244 genes upregulated from V_Endo to V_HSPC and then downregulated in V_Hem populations provided an "HSPC signature" (Data S2), including the known TFs *GFI1* and *MYB* (Thambyrajah et al., 2016; Mucenski et al., 1991; Labastie et al., 1998) but also the less investigated *HLF* and *SETBP1* (Goyama et al., 2008; Yokomizo et al., 2019; Figure 3A) and the TEK ligand *ANGPT1*. (2) Other genes were upregulated from V_Endo to V_HSPC and stayed upregulated in V_Hem (e.g., the essential hematopoietic TFs *RUNX1* and *SPI1*). (3) Here genes were expressed in V_Endo and V_HSPC but downregulated in the V_Hem population (e.g., the TFs *HOXA9*, *HEY2*, *GATA2*, and *SOX18*). (4) Some genes were downregulated in the V_HSPC population compared with V_Endo and V_Hem (e.g., the TF *MAF*, the zinc finger *ZFP36L1*, and the secreted factors *A2M*, *VCAN*, and *IGFBP5*), suggesting that their inhibition may be required for HSPC production by the endothelium (Figures 3A and 3B). (5) Finally, some genes were upregulated in the V_Hem population compared with V_HSPC and V_Endo (e.g., the TFs *GAS7* and *AFF3* and the secreted factor SPP1) (Figure 3C; Data S2).

Further GSEA allowed us to assign ventralized pathways detected by LCM-seq (TGF-β signaling, TNF-α signaling via NF-κB, and MTORC1) to specific EHT populations. TGF-β signaling was assigned to V_Endo, MTORC1 to V_HSPC populations, and TNF-α signaling via NF-κB to V_HSPC and V_Hem populations (Figure 3D). Notch signaling, enriched in the V_Endo and V_Hem populations, was downregulated in the V_HSPCs population, in line with reports that Notch signaling inhibition is required for EHT (Tang et al., 2013; Lizama et al., 2015; Souilhol et al., 2016b; Gama-Norton et al., 2015). In contrast, Myc targets were upregulated in V_HSPC versus V_Endo and V_Hem, in line with c-Myc involvement in hematopoietic development and adult HSC function (Wilson et al., 2004; Dubois et al., 2008).

Modeling the Niche Interactome

We then mapped predicted molecular interactions between secreted niche factors (V_Inner/Mid, LCM-seq2) and highly expressed genes in the V_Endo population using StringDB (Figure 3E). *EDN1* showed interactions with *SPP1* (osteopontin) and the V_Endo TF *GATA4*. Of note, SPP1 and GATA4 have well-documented roles in cardiac remodeling and hypertrophy, in line with ventral enrichment of the cardiac EGF pathway (Figures 1E' and 2A; Hautala et al., 2001; Mohamed et al., 2015; Oka et al., 2006). *SPP1* also showed a large number of interactions with V_Endo genes (SOST, FGF23, ITGB3, POU5F1, and PPARG) and other niche factors, including *REN* (8 total interactions, confidence > 0.4) (Figure 3E). To infer receptor-ligand interactions between ventral LCM-seq2 and the sorted populations, we used CellPhoneDB (Efremova et al., 2020; Figures S4A and S4B). Notable putative receptor-ligand interactions ($p < 0.01$) included *ANGPT2* (V_Inner/Mid) with the receptor *TEK* (TIE2) (V_Endo population) and *FGF1*, *FGF3*, *FGF4*, *FGF17*, and *FGF19* (V_Inner/Mid) with the receptor FGFR4 (V_HSPC population). As expected, V_Inner has more significant Notch receptor-ligand interactions with V_Endo than V_HSPC (V_Inner *NOTCH3/4*, V_Endo *JAG2/DLL4*; V_Inner *JAG1*, V_Endo *NOTCH4*), which corresponds to downregulation of Notch signaling as EHT progresses (Gama-Norton et al., 2015; Souilhol et al., 2016b) (for a full matrix of receptor-ligand interactions, see Data S2).

Endothelin/Renin Interactive Signaling

The sorted hematoendothelial populations showed no or low *REN* expression, consistent with its high expression in V_Mid but not V_Inner (Figure 3F). *AGTR1/2* angiotensin receptor expression was low, whereas the *ATP6AP2* prorenin receptor was substantially expressed in all hematoendothelial populations, suggesting renin action via *ATP6AP2* independent of ACE. *EDN1* was most highly expressed in endothelial populations (especially in V_Endo, as stated previously), downregulated

Figure 3. Dynamics in Gene Expression across the Endothelial-to-Hematopoietic Transition (EHT)

(A–C) Heatmaps of relative expression levels for TFs (A), secreted factors (B), and receptors (C) that are differentially expressed in V_Endo versus V_HSPC and in V_HSPC versus V_Hem (p.adj < 0.05).

(D) Patterns of signaling along EHT. Shown is GSEA pathway enrichment for V_Endo versus V_HSPC populations and V_HSPC versus V_Hem populations. Overlapping pathways enriched in V_HSPC in both comparisons are shown in red text (FDR < 0.25).

(E) StringDB interactions between LCM-seq1 V_Inner (versus D_Inner) and V_Mid (versus D_Mid) secreted factors (red) and V_Endo (versus D_Endo) genes (blue). The size of a node indicates the number of connecting edges. The width of a line indicates confidence of the interaction; confidence levels = 0.4–0.9.

(F) Dot plots of normalized expression levels of endothelin and renin core pathway genes for hematoendothelial populations.

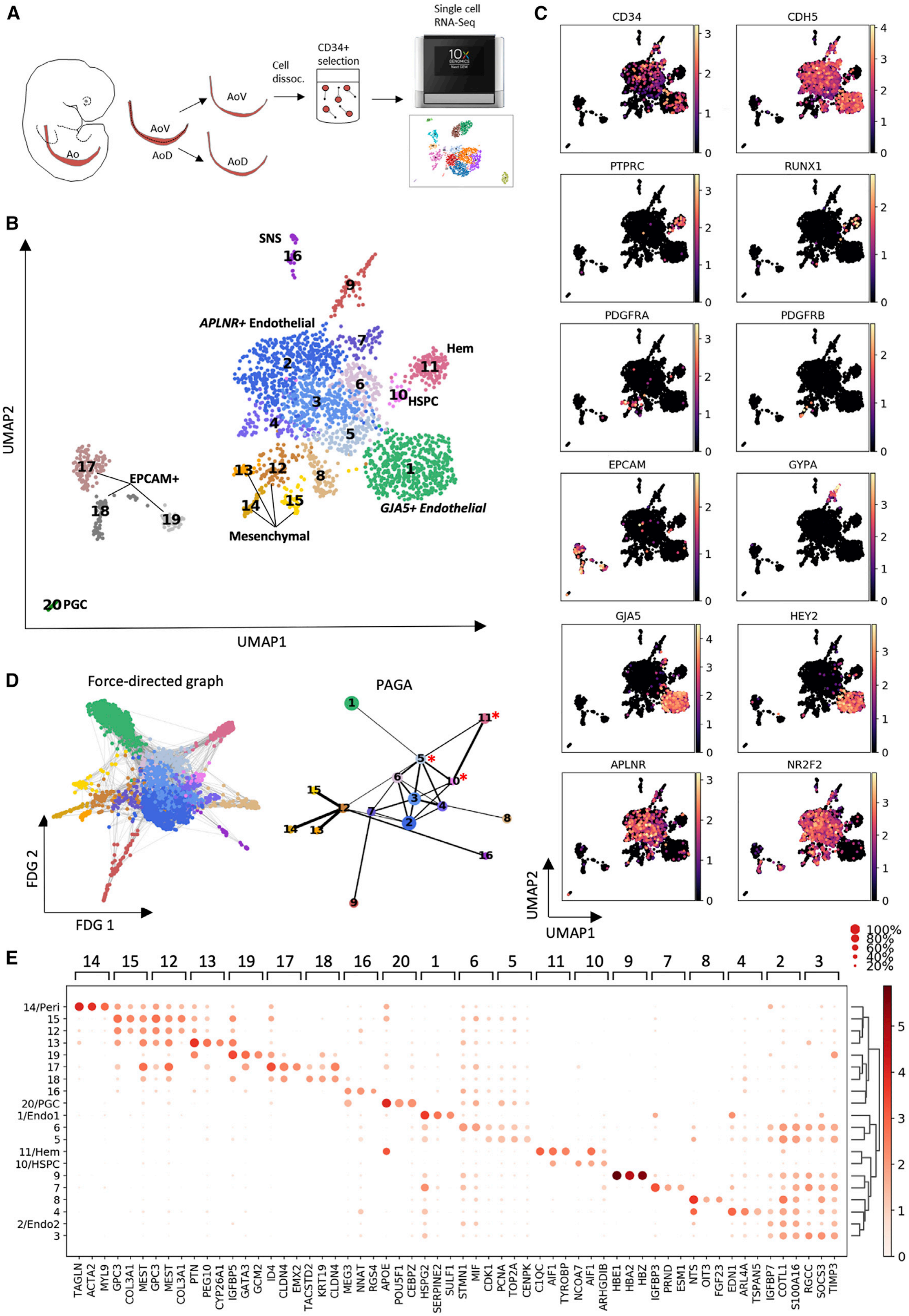

(legend on next page)

to a minimum in the V_HSPC population, and moderately upregulated in hematopoietic (V+D_Hem) populations. The endothelin-activating enzyme *ECE1* was considerably expressed in all populations, peaking on endothelium (V+D_Endo), and the endothelin receptors *EDNRA* and *EDNRB* in all hematoendothelial populations with low but clear expression in V_HSPC, suggesting a direct effect of endothelin on EHT.

Single-Cell RNA-Seq Analysis Reveals Direct Ancestors of HSPCs

Exploring Hematoendothelial Heterogeneity

To gain further insight, we explored the hematoendothelial heterogeneity in AoV (CS16) (Figure 4A). CD34+ cells from manually subdissected AoV were purified using beads and transcriptionally profiled using 10X Genomics. Contaminating CD34− cells (~21%) allowed additional resolution of non-hematoendothelial niche components. 2,379 cells were clustered using a nonlinear dimensionality reduction technique, uniform manifold approximation and projection (UMAP), followed by the Leiden algorithm to produce 20 clusters (CLs) (Figure 4B).

Visualizing gene markers (Figure 4C) enabled assigning basic cell-type identities to the CLs (Figure 4B). CL12 represents a population branching into 3 distinct mesenchymal subtypes: perivascular *ACTA2+PDGFRB+* CL14 and two unidentified ones, *CYP26A1+* CL13 and *SPRR2F+* CL15 (Figures 4B, 4C, and S5A). Standing alone, CL16 likely represents a population of sympathoadrenal progenitors co-expressing *TH* with several neuronal markers; e.g., *STMN2*, *ASCL1*, and *CADM1* (Figure S5A; DataS3). There are also 3 separate epithelium-like *EPCAM+* CLs: *EMX2+* CL17, likely related to the mesonephric epithelium (Pellegrini et al., 1997); *PERP*^{hi} CL18 of unclear identity; and CL19, exclusively expressing *PTH* (parathyroid hormone), an HSC regulator in adult bone marrow (Calvi et al., 2003; Figures 4B and S5A). The small CL20 represents *POU5F1+ NANOS3+* primordial germ cells (PGCs) (Figures 4B and S5A).

CD34 and *CDH5* mapped 10 CLs (CL1–CL10) containing endothelial cells or HSPCs (Figures 4B and 4C). A partition-based graph abstraction (PAGA) topology tree shows a central group of interconnected endothelial CLs, CL2–CL6 (Figure 4D), referred to as the central endothelial network. These CLs express the venous markers *APLNR* and *NR2F2* (*COUP-TFII*), as do the branching endothelial CLs CL7–CL9 (Figure 4C). The large CL1 linked weakly to the central endothelial network, expresses predominantly arterial markers (*GJA5* and *HEY2*), and is referred to as arterial endothelium (Figures 4C and 4D). However, some markers considered to be arterial, including *SOX7*, *SOX17*, *NOTCH1*, and *NRP1*, are expressed across the central endothelial network and CL1, suggesting ambiguity in distinction between arterial and venous endothelium (Figure S5B; Corada et al., 2013; Kim et al., 2007; Pendeville et al., 2008; Villa et al., 2001; Yamamizu et al., 2010; Herzog et al., 2001). Furthermore, the *ETS1* and *CD93* markers associated with the HSC program expressed across all endothelial populations captured in this dataset (Figure S5B; Bertrand et al., 2005; Park et al., 2018; Ciau-Uitz et al., 2013; Taoudi et al., 2005). CL9, expressing the erythroid marker *GYPA* and the embryonic/fetal hemoglobins *HBE1*, *HBZ*, and *HBA2*, represents primitive erythroid progenitors (Figures 4C and 4E).

Analysis of Lineage Relationships

Two CLs adjacent to the endothelial network represent the hematopoietic lineage (CL10 and CL11; Figure 4B). The small CL10 immediately attached to the endothelial network is the HSPC population co-expressing endothelial and hematopoietic *CDH5+PTPRC+*(*CD45+*) determinants (Figure 4C), marked by the transcription enhancer gene *NCOA7* identified in our HSPC signature (Figures S5A and 3C). HSPC CL10 is directly linked to a more mature hematopoietic CL11 with simultaneous *PTPRC* up- and *CDH5* downregulation (Figures 4C and 4D). Intriguingly, PAGA most confidently links HSPC CL10 with *APLNR+-COUP−TFII+RUNX1−* CL5, making it the most likely candidate for hematogenic endothelium (Figure 4D). The HSPC population shows downregulation of venous markers and minimal arterial marker expression (*GJA5* and *HEY2*). The force-directed graph also indicates a lineage relationship between arterial CL1 and CL5, suggesting that CL5 is a "bridge" population between the arterial endothelium and hematopoietic populations (Figure 4D). The non-arterial signature of CL5 likely reflects COUP-TFII-mediated downregulation of Notch signaling, required for HSC maturation (Tang et al., 2013; Lizama et al., 2015; Souilhol et al., 2016b; Gama-Norton et al., 2015; You et al., 2005). Notably, CL5 is enriched for cell cycle-related pathways (Data S3) associated with elevated expression of *TOP2A*, *PCNA*, and *CDK1* (Figure S5C). This is in line with the previous finding that EHT toward mature mouse HSCs is accompanied by activation of proliferation (Batsivari et al., 2017). Additional single-cell analysis of limited cell numbers from a second CS16 embryo also showed a lineage relationship between *COUP-TFII* endothelium (*CDH5+-PTPRC−*) and HSPCs (*CDH5+PTPRC+*) (Figure S5D).

Cell Population-Specific Resolution Suggests Autocrine and Paracrine Endothelin 1 Action

To identify source populations of ventrally enriched secreted factors detected above, all such V_Inner/Mid factors (LCM-seq2) were mapped onto the CS16 AoV single-cell dataset (Figure 5A).

Figure 4. Exploring Heterogeneity of the Hematoendothelial Population Using Single-Cell RNA-Seq Analysis

(A) Experimental strategy for single-cell analysis. A CS16 Ao was bisected into V (AoV) and D (AoD) domains. CD34+ cells were bead purified from the AoV cell suspension and subjected to 10X single-cell sequencing.

(B) UMAP with Leiden clustering of the CS16 AoV single-cell dataset (N = 1, n = 2,379 single cells). Hem, hematopoietic; HSPC, Hem stem/progenitor cell; SNS, sympathoadrenal nervous system). The central endothelial network (CL1–CL7) links with large arterial (*GJA5+*) CL1 and HSPC cluster 10 (CL10), which transits into mature blood cells (CL11). Note that the arterial CL1 linked to HSPC CL10 via the bridge CL5 with a downregulated arterial signature (evident from force-directed graph visualization; D). For a detailed description of all CLs, see Results.

(C) Mapped ln-normalized expression of key genes identifying cell subtypes.

(D) Force-directed graph visualization and partition-based graph abstraction (PAGA) topology tree of cells from (B), excluding CL17–CL20. Note that HSPC CL10 is most strongly linked to the bridging CL5 with a downregulated arterial signature and downstream mature blood cells (CL.11), highlighted by asterisks. The width of edges in PAGA indicate the strength of connectivity between CLs.

(E) Mean expression for top 3 markers in each CL revealed in (B) (the plot is ln normalized).

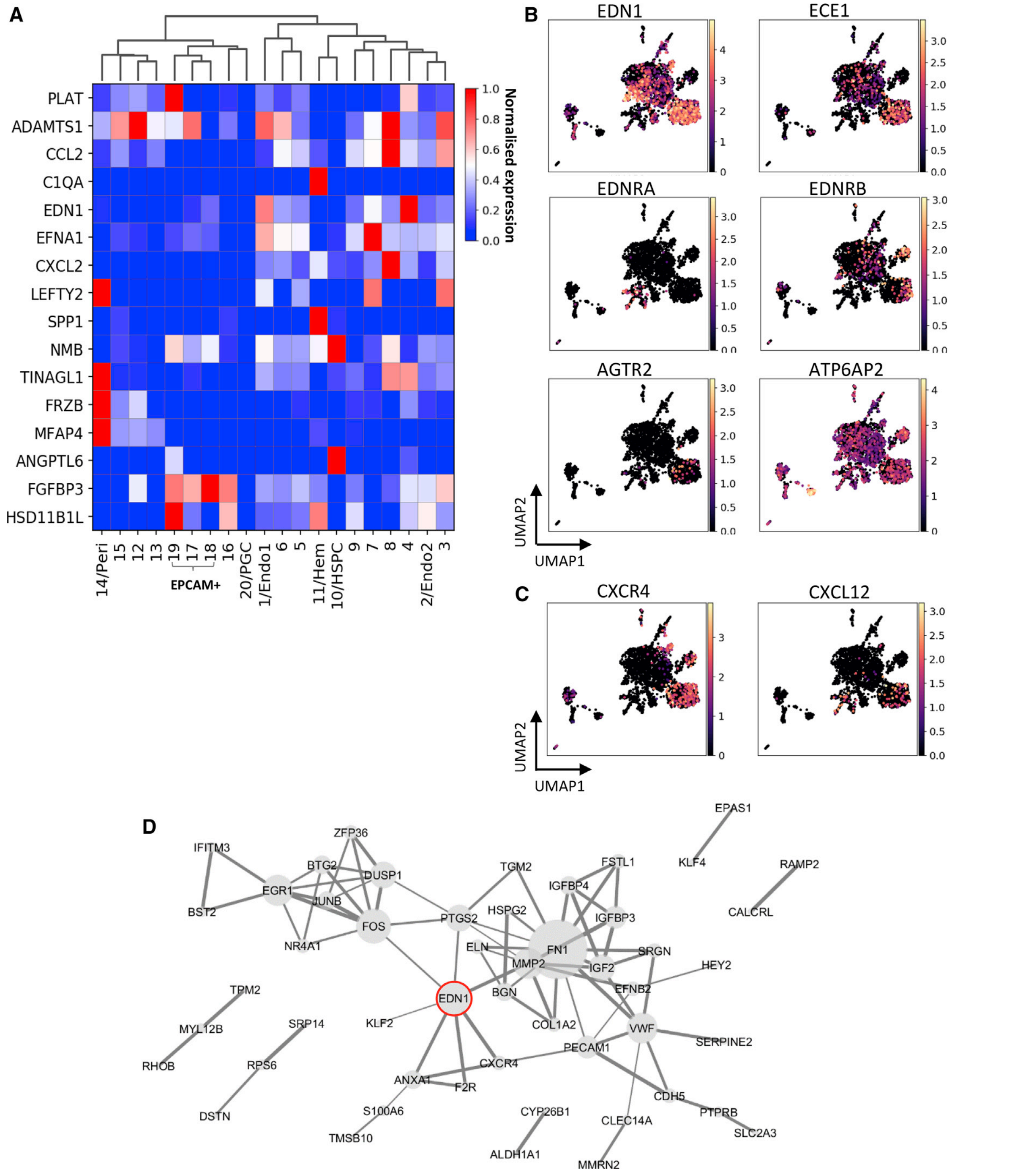

Figure 5. Mapping LCM-Seq Niche Signals to Cell Populations in the AoV Suggests Autocrine and Paracrine Signaling of Endothelin 1

(A) Expression of differentially expressed secreted factors detected in the V_Inner and V_Mid subdomains (LCM-seq1) in cell CLs shown in Figure 4B.

(B) Mapping expression of endothelin and renin core pathway genes on the CS16 AoV single-cell dataset (the plot is ln normalized).

(C) Mapping expression of CXCR4 and CXCL12 to cell CLs shown in Figure 4 (the plot is ln normalized).

(D) StringDB network of high-confidence known and predicted interactions between genes expressed in arterial CL1, including direct protein-protein physical and indirect functional associations (confidence > 0.7). The size of a node indicates the number of connecting edges. The width of a line indicates confidence of the interaction; confidence levels = 0.4–0.9.

We found that the hematopoietic CL11 has highest expression of *SPP1* involved in adult HSC migration and proliferation (Nilsson et al., 2005) and complement factor *C1QA*, in line with expression in the V_Hem population (hematoendothelial dataset; Figure 3B; Data S2). The endothelial CL8 produces the metalloproteinase *ADAMTS1* and chemokines *CCL2* and *CXCL2* with

potential to recruit monocytes and other immune cells to the AoV (Ajuebor et al., 1998). Pericytes (CL14) secrete the Wnt signaling modulator *FRZB* (Cruciat and Niehrs, 2013), the inhibitor of transforming growth factor β (TGF-β) and BMP signaling *LEFTY2* (Ulloa and Tabibzadeh, 2001), as well as *TINAGL1* and *MFAP4*. Epithelial *EPCAM*+ CL19 is a source of *HSD11B1L* and the plasminogen activator *PLAT*. Additionally, the top ventrally polarized endothelial genes *GATA4* and *FGF23* from the hematoendothelial sort dataset (V_Endo versus D_Endo) were markedly expressed in the endothelial CL8 (Figure S5A), indicating its potential significance in the HSC niche.

Most components of the renin-angiotensin pathway are not detectable in the single-cell dataset, except for the angiotensin receptor *AGTR2*, localized to the arterial CL1, and prorenin receptor *ATP6AP2* global expression, particularly high in CL19 (Figure 5B). Although *EDN1* localized broadly in the endothelial central network with particularly high and consistent expression in CL1, CL4, and CL7, the strongest *ECE1* (activating enzyme) expression indicates that arterial CL1 is the main source of active endothelin 1 (Figure 5B). Furthermore, the chemokine *CXCL12* and its receptor *CXCR4*, involved in HSC development and function, are expressed in CL1 (Figure 5C; Lapidot and Kollet, 2002; Wright et al., 2002; Sugiyama et al., 2006). CL1 is also a strong source of Notch ligands (*DLL4*, *JAG1*, and *HEY2*) (Figure S5A). Co-expression of all these markers indicates the importance of arterial endothelium in the developing HSC niche (Hadland et al., 2015). Expression of the endothelin receptor *EDNRA* is mainly confined to the mesenchymal populations (CL12–CL15), whereas *EDNRB* is expressed mostly in the arterial endothelial CL1 and hematopoietic CL11, consistent with expression in the V_Endo and V_Hem fractions. Thus, endothelin 1 signaling emanating from the Ao endothelial lining potentially acts in an autocrine and paracrine (mesenchymal and hematopoietic) manner. Notably, StringDB interactome analysis of CL1 places *EDN1* in the center, connecting it with other central signaling molecules: *FOS*, *MMP2*, *PTGS2*, and *CXCR4* (Figure 5D).

Hotspots of Endothelin 1 Expression Correlate with Localization of IAHCs

EDN1 and *REN* expression patterns were analyzed at the RNA and protein levels using RNAScope and immunofluorescence. Despite high *REN* expression in the [V]+[VL] subdomains (LCM-seq1) and the V_Mid subdomain (LCM-seq2), it was detected only in occasional cells immediately below the ventral endothelium (Figure 6A). However, higher REN expression was observed along the vessels branching ventrolaterally from the Ao toward the mesonephros (Figure 6B). Hematoendothelial cells are potential targets for REN because of expression of the receptor *ATP6AP2* (Figure 3F).

Despite *EDN1* expression by endothelial cells across the entire D-V axis (Figures 6A and 6D''), some endothelial cells had a higher density of the *EDN1* probe making a signal cluster (>300 pixels/2,048 × 2,048 pixel image), referred to as *EDN1* hotspots (Figures 6C–6E). Quantification determined significantly more *EDN1* hotspots in the AoV compared with the AoD (paired t test, $p < 0.01$), in line with *EDN1* enrichment in sorted ventral endothelium (Figure 6E). Furthermore, *EDN1* hotspots are frequently located proximal to Runx1+ cells or IAHCs with strong statistical correlation (Figures 6C, 6D, and 6G). Some lower *EDN1*-expressing mesenchymal cells were also detected below IAHCs (Figure 6Ai). This suggests that high *EDN1* expression near IAHCs may play a role in HSPC development.

Notably, immunostaining detected a strong EDN1 protein presence in IAHCs (Figures 6F and S6A). Because our population and single-cell analyses showed only minimal *EDN1* mRNA expression in HSPCs (Figures 3F and 5B), we assume sequestering of external EDN1 protein onto emerging IAHCs through receptor binding. Indeed, low EDNRA levels were detected within IAHCs, in line with moderate expression in HSPCs (Figures 3F and S6B), in addition to broad expression in the mesenchyme (Figures 5B, CL12–CL15); 6D'', and S6C). Although we cannot fully exclude autonomous EDN1 expression within IAHCs, this is unlikely to be due to predominant association of EDN1 immunostaining with the cell membrane and not with the cytoplasm. The receptor EDNRB was not detectable in IAHCs but observed frequently in single budding cells (Figure S6A) and weakly in the endothelium. Strong EDNRB expression was observed in presumably ventrally migrating neural crest derivatives (Figures 6D'' and S6C; Nataf et al., 1996). Some *EDNRB*+ cells can be macrophages of hematopoietic CL10 (single-cell dataset; Figure 5B).

Endothelins Enhance Hematopoietic Development in the Mouse and Human Systems

We first investigated whether endothelin signaling can promote adult-type hematopoiesis using the mouse model. Previous spatial transcriptome analysis determined that mouse *Ren1* was ventrally enriched in embryonic day 9.5 (E9.5) and E10.5 AoV but *Edn1* in E9.5 AoV only (Figure S7A; McGarvey et al., 2017). The similar isoform *Edn2*, ventrally enriched at E9.5 and E10.5 (which differs from *Edn1* by 3 amino acids only), was included in the functional analysis. As in the human, ventral hotspots of *Edn1* mRNA were detected proximal to emerging IAHCs (Figure S7B). Similarly, EDN1 protein was detected on CD31+-cKit+ IAHCs (E10.5), mainly on cell membranes exposed to the Ao lumen, and excluded from cell-cell contact interfaces, suggesting sequestering via receptors (in contrast to CD31 and cKit) (Figure 7A). Weaker EDN1 staining was also observed throughout the subaortic mesenchyme.

To establish whether endothelin signaling can promote adult-type hematopoiesis, we used an *ex vivo* system recapitulating HSC development (Rybtsov et al., 2014; Figure 7B). Reaggregates of E9.5 caudal tissues were cultured on floating membranes with addition of one of the factors REN1, EDN1, or EDN2 (each in two concentrations: 10 and 100 ng/μL). After 7 days, reaggregates were plated in a colony-forming (CFU-C) assay. Although total CFU-C numbers did not change markedly (Figure 7C), each factor significantly shifted CFU-C production from CFU-Macs to more immature CFU-GEMMs (in a dose-dependent manner for EDN1 and EDN2, Fisher's test, $p < 0.001$) (Figure 7D).

Reaggregate cultures were also transplanted into irradiated Ly5.1 mice, and hematopoietic (CD45_2) repopulation was assessed at 8 and 18 weeks (3 experiments) (Figure 7E). All 3 factors enhanced long-term multilineage repopulation in the peripheral blood, spleen, and bone marrow compared with controls (statistically significant for REN [100 ng/μL] and EDN2 100 ng/μL], $p < 0.05$, paired t test) (Figures 7E and S7C–S7F).

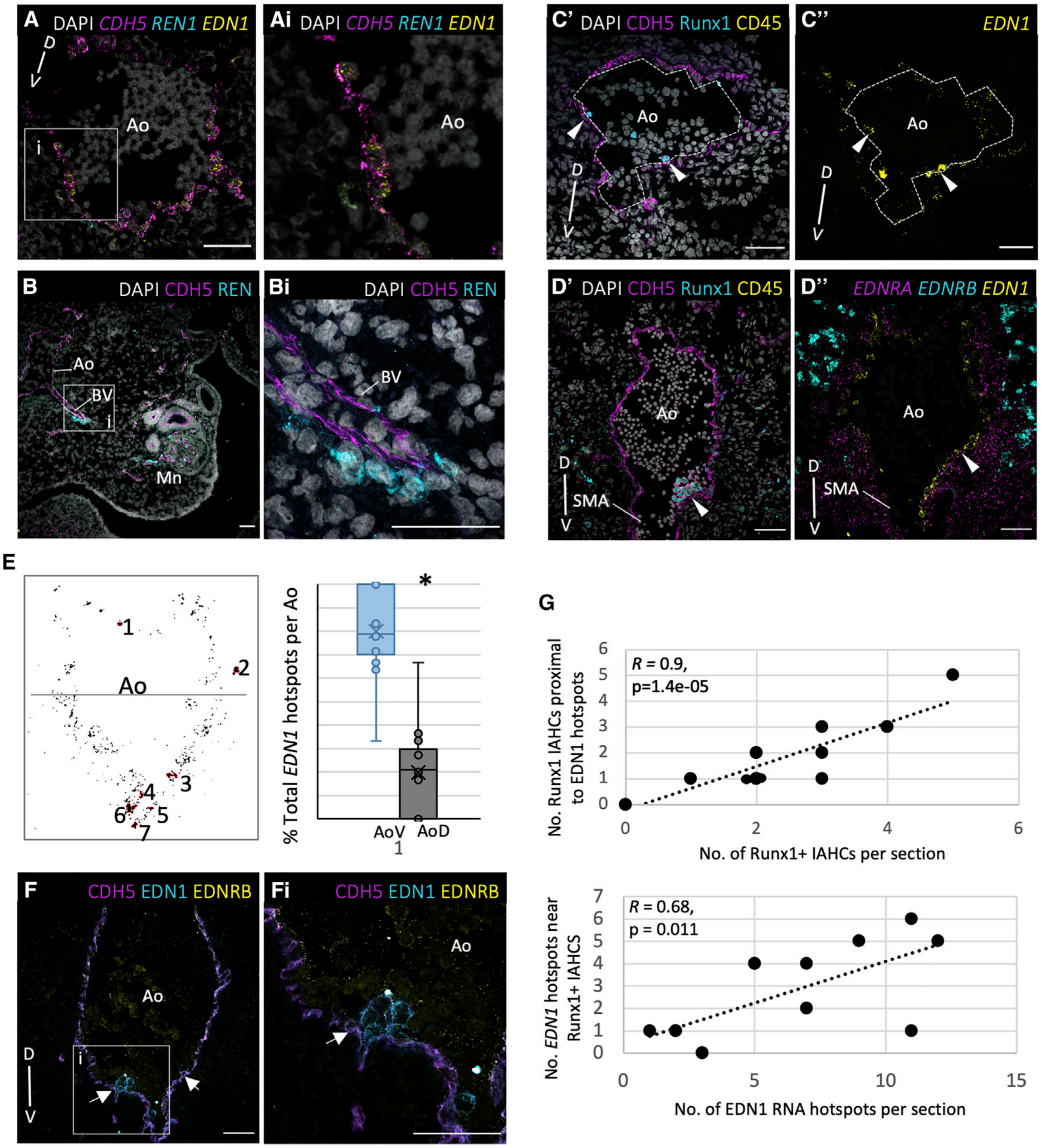

Figure 6. Endothelin-1 Expression Highly Correlates with Localization of IAHCs

(A) Expression overlap between *EDN1*, *CDH5*, and *REN1*. The arrowhead indicates a *REN1*+ cell below the *CDH5*+ endothelium. The images in (Ai) show a magnification of the boxed region in (A).

(B) Expression of REN+ cells enveloping the endothelium of an Ao V branching vessel (BV) directed toward the Mn. The images in (Bi) show a magnification of the boxed region in (B).

(C and D) Immunostaining highlighting V CDH5+Runx1+CD45+ IAHCs (C' and D') and, on the sister section, a higher *EDN1* signal in a corresponding position (C'' and D''). Arrowheads indicate positions of IAHCs.

(E) Representative binary image of *EDN1* expression across the Ao with *EDN1* hotspots (pixels > 300, 2,048 × 2,048 pixel image) numbered and outlined in red. A line divides the AoD (top) from the AoV (bottom). The box-and-whisker plot shows the percentage of *EDN1* hotspots found in the AoV or AoD in each section (n = 14). $p < 0.01$, t test.

(F) CL of rounded EDN1-expressing cells attached to the CDH5+ endothelial lining (arrows). Images in (Fi) show a magnification of the boxed region in (F).

(G) Correlation between the position of Runx1+ IAHCs in each section with the position of *EDN1* hotspots (*R*, correlation coefficient).

For (A)–(C), (E), and (F), protein or RNA expression is indicated by non-italicized and italicized names, respectively. SMA, superior mesenteric artery. The D-V axis is indicated. Images show transverse sections of CS15–CS16 embryos. Scale bars, 50 μm.

CellPress

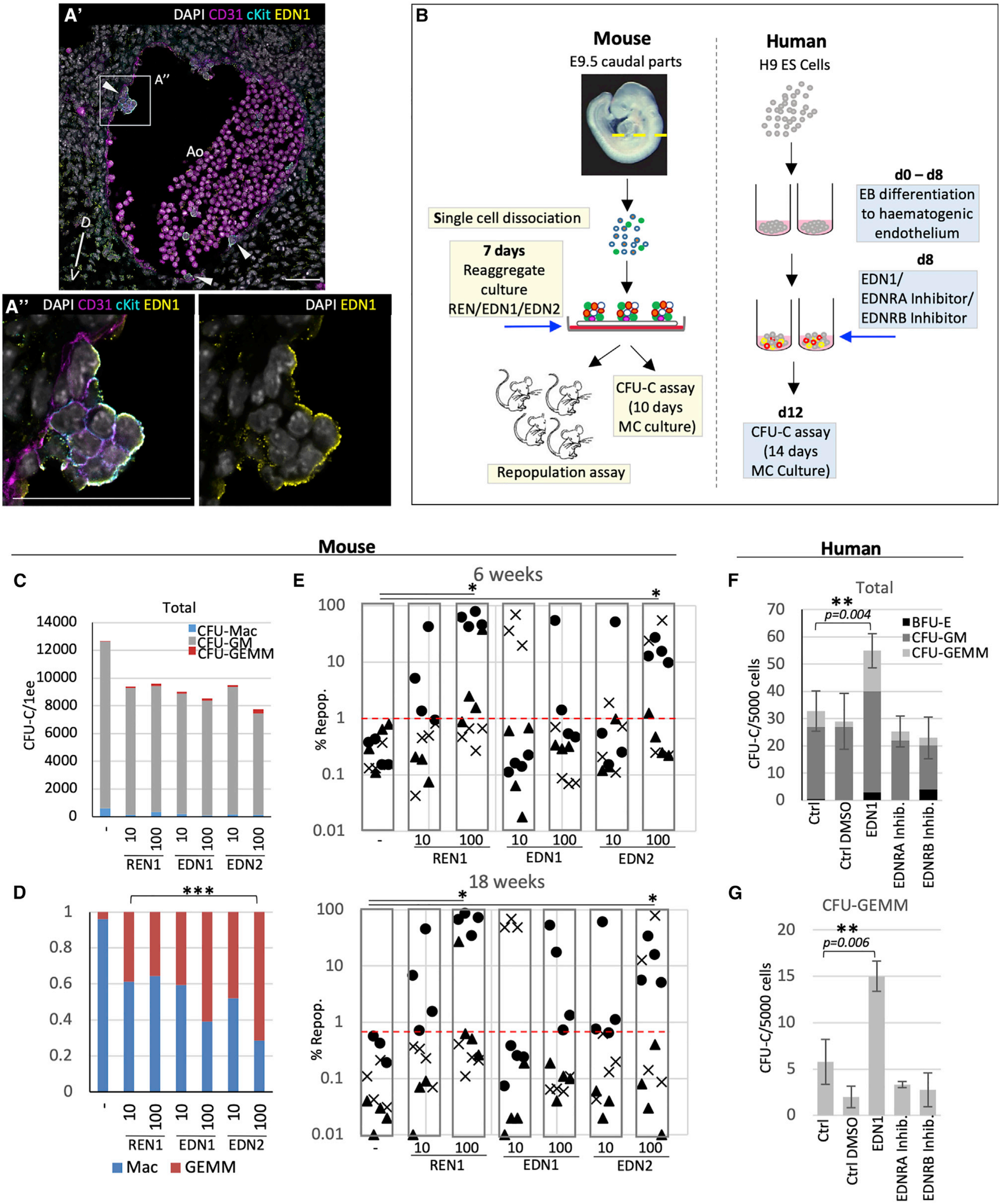

Figure 7. Endothelins Promote Hematopoiesis in the Mouse and Human Model Systems

(A) CD31, cKit, and EDN1 immunostaining in the Ao of an E10.5 mouse embryo. Arrowheads indicate cKit+EDN1+ IAHCs or individual emerging Hem cells. The images in (A'') show a magnification of the boxed region in (A'). Scale bars: 50 μm.

(B) Experimental strategies for mouse and human model systems. MC, methocult.

(legend continued on next page)

We then explored the effects of endothelin on human hematopoietic development. Because human AGM region cultures showed limited potential for analysis of HSC development (Easterbrook et al., 2019), we employed human embryonic stem cells (hESCs), used broadly to model hematopoietic development (Figure 7B). In contrast to the mouse, EDN1 was differentially expressed in the human HSC niche (EDN2 showed no expression and was not used for analysis).

hESCs were cultured as described previously (Ng et al., 2016). At day 8, during induction of hematogenic endothelium, individual cultures were supplemented with 100 ng/μL EDN1, 1 nM EDNRA inhibitor, or 100 nM EDNRB inhibitor. Within 4 days, 5,000 cells from each culture were plated in duplicates in methylcellulose culture, and colonies were scored 14 days later. We found that EDN1 significantly increased CFU-C production compared with control cultures, including a significant increase in complex CFU-GEMM colonies (paired t test, $p < 0.01$) (Figures 7F and 7G). Some reductions in total CFU-C numbers supplemented with EDNRA and EDNRB inhibitors were not significant.

DISCUSSION

Developing HSCs are embedded in and influenced by the highly heterogeneous molecular milieu of the AGM region. Like many other developmental processes, HSC development is underpinned by embryonic inductive interactions and cross-signaling between adjacent tissues and regions. Although model organisms provide valuable insights into the mechanisms underlying hematopoietic development, these cannot be extrapolated directly to humans because of the specifics of hematopoietic development (Easterbrook et al., 2019).

We transcriptionally profiled sorted Ao hematoendothelial populations (endothelial cells, HSPCs, and mature blood cells) that allowed us to track signaling dynamics during EHT. To further increase resolution, we performed single-cell RNA-seq analysis, which revealed rich cellular heterogeneity within the Ao. We identified a central endothelial network consisting of 5 populations expressing venous markers (*APLNR*+, *COUP-TF2*+) with a loosely connected large arterial CL (*GJA5*+, *HEY2*+, and upregulated Notch signaling). The CDH5+CD45+ HSPC population (Ivanovs et al., 2014) was closely associated with the central endothelial network and, unexpectedly, was indirectly linked to the arterial CL via a bridging endothelial population with a downregulated arterial signature. Many previous studies have recognized the close relationship between arterial and hematopoietic developmental programs (Clements and Traver, 2013; Park et al., 2018)). This bridging population showed increased proliferation, in line with previously reported activation of proliferation during HSC maturation in the mouse (Batsivari et al., 2017) suggesting similarity to the bridging population with mouse pro/pre-HSCs (Taoudi et al., 2008; Rybtsov et al., 2011, 2014). Downregulation of arterial markers in the bridging population is likely a consequence of downregulation of NOTCH signaling (described as a prerequisite for HSC/IAHC maturation in the mouse), probably because of upregulation of COUP-TFII (a Notch suppressor) observed in our dataset (Lizama et al., 2015; Bos et al., 2015; Souilhol et al., 2016b; Tang et al., 2013; Richard et al., 2013; Gama-Norton et al., 2015; You et al., 2005).

Our observation is in line with evidence that hESC-derived hematogenic endothelium is negative for arterial (*KDR*) and venous (*NT5E*) markers and emerges separately from the arterial endothelium (Ditadi et al., 2015). It is likely that the recently proposed arterial origin of hematogenic endothelium at CS13 (Zeng et al., 2019) can be explained by exclusion of venous cells from analysis. Notably, the cells in our dataset expressing venous markers also expressed classical arterial markers, including neuropilin 1 (*NRP1*) and *SOX17* (Herzog et al., 2001; Corada et al., 2013). In the early mouse Ao, some cells also co-express arterial and venous markers (Lindskog et al., 2014). The distinction between arterial and venous identity during embryogenesis is therefore less defined than usually presumed.

We interrogated human HSC developmental niche signaling using spatial transcriptomics to map ventrally polarized signals potentially associated with IAHCs/HSCs emergence. Signaling in close proximity to IAHCs/HSCs included some known pathways involved in HSC development: TNF-α signaling via NF-κB (Espín-Palazón et al., 2014), MTORC1 signaling (Zhou et al., 2016), and NO signaling (North et al., 2009; Adamo et al., 2009). Here we decided to focus on the ventrally enriched cardiac EGF pathway, which was not implicated previously in HSC development. A major regulator of this pathway, *EDN1* (which also contributes to TNF-α signaling via NF-κB), showed strong ventral polarization. Interactome analysis revealed a high number of endothelin 1 interactions with other niche factors, suggesting its key role in the developing HSC niche. Our analyses also revealed ventral polarization of renin along with other components of the renin-angiotensin pathway, which has a known reciprocal relationship with endothelin 1 (Beierwaltes and Carretero, 1992; Lin et al., 1993; Ackermann et al., 1995; Rossi et al., 1999). Based on these data, we focused on investigation of endothelin 1 and renin functions in HSPC development.

Functional analysis in the mouse system demonstrated a strong influence of endothelin signaling on hematopoietic development because EDN1 and EDN2 increased the number of early multipotent progenitors (CFU-GEMM) in E9.5 caudal tissue *ex vivo* (Rybtsov et al., 2014). Importantly, addition of EDN2 (and REN1) substantially stimulated mouse HSC development. Furthermore, using the hESC culture system, we demonstrated

(C) Production of CFU-Cs in E9.5 caudal tissues cultured with each of the test factors (REN1, EDN1, and EDN2) at 10 ng/μL and 100 ng/μL. n = 4 independent experiments.

(D) Proportion of CFU-Mac and CFU-GEMM from (B), normalized to 1; n = 4 independent experiments.

(E) Repopulation with E9.5 caudal part cells cultured with the test factors (REN1, EDN1, and EDN2) and 18 weeks following transplantation (*$p < 0.05$, paired t test, n = 3 independent experiments). The shape on the graph indicates the experiment. The red line shows the upper limit of control repopulation levels). The numbers of long-term repopulating mice (18 weeks) higher than 1% are as follows: CTRL, 0/11; REN1_10, 3/11; REN1_100, 5/12; EDN1_10, 3/11; EDN1_100, 3/12; EDN2_10, 2/11; EDN2_100, 6/12.

(F) Effect of test factors on production of CFU-Cs by human ES cells at 12 days of differentiation. The number of day 14 CFU-Cs per 5,000 plated cells is shown; factors were added at day 8 of culture (**$p < 0.01$, paired t test; error bars, ± SEM, n = 4).

(G) Production of CFU-GEMM from (E) (**$p < 0.01$, paired t test; error bars, ± SEM).

that EDN1 (the major isoform in the human AGM region) significantly increased production of early multipotent hematopoietic progenitors at the expense of unipotent CFU-M. Collectively, these experiments show that endothelins are important regulators of HSC development in humans.

Our transcriptome analyses provided some insight into potential mechanisms of endothelin action. *EDN1* is expressed mainly by endothelial fractions and mature blood cells. However, to become active, it requires processing with the maturation factor ECE1, which is expressed mainly by the arterial CL, suggesting that it is the main source of active EDN1 in the niche. Here EDN1 is well positioned to signal to all main constituents of the niche. The hotspots of EDN1 discovered in this study immediately adjacent to IAHCs/HSCs can directly influence emerging HSPCs and single cells budding into the lumen through EDNRA and EDNRB, respectively. Additionally, EDN1 may act on HSC development indirectly, via other AGM populations that express endothelin receptors, including endothelium, pericytes, mesenchymal cells, the neural crest, and macrophages. For example, EDNRB+ ventral endothelial cells might mediate EDN1 action, being in immediate contact with developing HSCs. Of note, the most significantly upregulated TF in the ventral endothelium, GATA4, is induced by endothelin 1 in response to increased blood pressure (Hautala et al., 2001; Kitta et al., 2001). This raises the possibility that GATA4 can be involved in EHT stimulation by shear stress (Adamo et al., 2009; North et al., 2009).

Combined transcriptome analysis revealed many other secreted factors that would be worth exploring further. One such example is *SPP1*, which has a documented role in the fetal and adult bone marrow, modulating HSC proliferation and migration (Nilsson et al., 2005; Cao et al., 2019; Stier et al., 2005), and binds CD44, recently implicated in EHT (Luo et al., 2011; Oatley et al., 2020). Our population and single-cell analysis showed that SPP1 is mainly generated by hematopoietic populations, perhaps by macrophages (Lund et al., 2009).

In conclusion, our study not only identifies important regulators of HSC development in humans but also provides a valuable resource for the community for in-depth investigation of mechanisms underlying development of the adult hematopoietic system in humans. Exploration of multiple heterogeneous cell populations as described here may inform new protocols for human HSC derivation from pluripotent cells for clinical applications.

Limitations of Study

Because of the rarity of human embryonic tissues, the sample numbers in this study are lower than optimal. Therefore, functional validation of secreted factors was performed using mouse embryo *ex vivo* and hESC *in vitro* models. Although significant, transplantation results between experiments are variable, likely reflecting natural heterogeneity and heterochrony in hematopoietic development between embryos (Taylor et al., 2010). Further analysis of direct endothelin 1 effects on human HSC development *in vivo* may become possible in the future when appropriate approaches become available.

STAR★METHODS

Detailed methods are provided in the online version of this paper and include the following:

- KEY RESOURCES TABLE
- RESOURCE AVAILABILITY
  - Lead Contact
  - Materials Availability
  - Data and Code Availability
- EXPERIMENTAL MODEL AND SUBJECT DETAILS
  - Human embryonic material
  - Mouse models
  - Cell lines
- METHOD DETAILS
  - Laser Capture Microdissection
  - Human embryo cell sorting
  - RNA-Seq Library Preparation
  - RNA-Sequencing
  - LCM-Seq and Bulk Population Transcriptome analysis
  - 10x Single Cell Analysis
  - Imaging
  - *Ex vivo* floating membrane aggregate cultures
  - ES Cell Differentiation
  - Colony-forming assays
  - Long-term repopulation assay
  - Flow cytometric analysis of cells
- QUANTIFICATION AND STATISTICAL ANALYSIS

SUPPLEMENTAL INFORMATION

Supplemental Information can be found online at https://doi.org/10.1016/j.stem.2020.08.004.

ACKNOWLEDGMENTS

We thank Anne Saunderson and Norma A. Forson (Clinical Research Staff, MRC Centre for Reproductive Health) for sourcing and providing human samples; additional human embryonic and fetal material was provided by the Joint MRC / Wellcome (MR/R006237/1) Human Developmental Biology Resource (www.hdbr.org); the CRM Animal Facility for mouse maintenance, breeding, and irradiation; Bertrand Vernay and Matthieu Vermeren for assistance with microscopy; and Elisa Laurenti, Katrin Ottersbach, and Steven Pollard for helpful feedback and advice. This work was supported by the Medical Research Council UK.

AUTHOR CONTRIBUTIONS

E.I.C. designed and performed (transcriptome, *in vitro*, *in vivo,* imaging, and flow cytometry) experiments and bioinformatics analyses, produced figures, and wrote the manuscript. S.G.-K. and S.R. helped with AGM dissection and *in vitro* and transplantation experiments. S.R. performed cell sorting. A.B.-C. helped prepare samples for 10X Genomics and provided input and feedback. H.F. performed immunostaining in mice. K.K. and T.C. helped with 10X Genomics settings. N.C.N. helped with the ESC differentiation protocol. S.T. provided guidance for immunostaining and *in vitro* experiments, input, and feedback. D.J.W. provided advice and endothelin inhibitors. F.R. performed cell sorting and provided flow cytometry guidance. R.A.A. provided access to human embryo samples, input, and feedback. A.M. conceived the study, supervised experimental design and interpretation, and wrote the manuscript.

DECLARATION OF INTERESTS

The authors declare no competing interests.

Received: March 27, 2020
Revised: May 25, 2020
Accepted: August 7, 2020
Published: September 17, 2020

REFERENCES

Ackermann, M., Ritthaler, T., Riegger, G., Kurtz, A., and Krämer, B.K. (1995). Endothelin inhibits cAMP-induced renin release from isolated renal juxtaglomerular cells. J. Cardiovasc. Pharmacol. *26* (*Suppl 3*), S135–S137.

Adamo, L., Naveiras, O., Wenzel, P.L., McKinney-Freeman, S., Mack, P.J., Gracia-Sancho, J., Suchy-Dicey, A., Yoshimoto, M., Lensch, M.W., Yoder, M.C., et al. (2009). Biomechanical forces promote embryonic haematopoiesis. Nature *459*, 1131–1135.

Ajuebor, M.N., Flower, R.J., Hannon, R., Christie, M., Bowers, K., Verity, A., and Perretti, M. (1998). Endogenous monocyte chemoattractant protein-1 recruits monocytes in the zymosan peritonitis model. J. Leukoc. Biol. *63*, 108–116.

Andrews, S. (2010). FastQC: A Quality Control Tool for High Throughput Sequence Data. http://www.bioinformatics.babraham.ac.uk/projects/fastqc/.

Ashburner, M., Ball, C.A., Blake, J.A., Botstein, D., Butler, H., Cherry, J.M., Davis, A.P., Dolinski, K., Dwight, S.S., Eppig, J.T., et al.; The Gene Ontology Consortium (2000). Gene ontology: tool for the unification of biology. Nat. Genet. *25*, 25–29.

Ayllón, V., Bueno, C., Ramos-Mejía, V., Navarro-Montero, O., Prieto, C., Real, P.J., Romero, T., García-León, M.J., Toribio, M.L., Bigas, A., and Menendez, P. (2015). The Notch ligand DLL4 specifically marks human hematoendothelial progenitors and regulates their hematopoietic fate. Leukemia *29*, 1741–1753.

Baron, C.S., Kester, L., Klaus, A., Boisset, J.C., Thambyrajah, R., Yvernogeau, L., Kouskoff, V., Lacaud, G., van Oudenaarden, A., and Robin, C. (2018). Single-cell transcriptomics reveal the dynamic of haematopoietic stem cell production in the aorta. Nat. Commun. *9*, 2517.

Batsivari, A., Rybtsov, S., Souilhol, C., Binagui-Casas, A., Hills, D., Zhao, S., Travers, P., and Medvinsky, A. (2017). Understanding Hematopoietic Stem Cell Development through Functional Correlation of Their Proliferative Status with the Intra-aortic Cluster Architecture. Stem Cell Reports *8*, 1549–1562.

Beierwaltes, W.H., and Carretero, O.A. (1992). Nonprostanoid endothelium-derived factors inhibit renin release. Hypertension *19* (2, Suppl), II68–II73.

Benjamini, Y., and Hochberg, Y. (1995). Controlling the False Discovery Rate: A Practical and Powerful Approach to Multiple Testing. J. R. Stat. Soc. Series B Stat. Methodol. *57*, 289–300.

Bertrand, J.Y., Giroux, S., Golub, R., Klaine, M., Jalil, A., Boucontet, L., Godin, I., and Cumano, A. (2005). Characterization of purified intraembryonic hematopoietic stem cells as a tool to define their site of origin. Proc. Natl. Acad. Sci. USA *102*, 134–139.

Bertrand, J.Y., Chi, N.C., Santoso, B., Teng, S., Stainier, D.Y., and Traver, D. (2010). Haematopoietic stem cells derive directly from aortic endothelium during development. Nature *464*, 108–111.

Boisset, J.C., van Cappellen, W., Andrieu-Soler, C., Galjart, N., Dzierzak, E., and Robin, C. (2010). In vivo imaging of haematopoietic cells emerging from the mouse aortic endothelium. Nature *464*, 116–120.

Bos, F.L., Hawkins, J.S., and Zovein, A.C. (2015). Single-cell resolution of morphological changes in hemogenic endothelium. Development *142*, 2719–2724.

Calvi, L.M., Adams, G.B., Weibrecht, K.W., Weber, J.M., Olson, D.P., Knight, M.C., Martin, R.P., Schipani, E., Divieti, P., Bringhurst, F.R., et al. (2003). Osteoblastic cells regulate the haematopoietic stem cell niche. Nature *425*, 841–846.

Cao, H., Cao, B., Heazlewood, C.K., Domingues, M., Sun, X., Debele, E., McGregor, N.E., Sims, N.A., Heazlewood, S.Y., and Nilsson, S.K. (2019). Osteopontin is An Important Regulative Component of the Fetal Bone Marrow Hematopoietic Stem Cell Niche. Cells *8*, 985.

Chen, M.J., Yokomizo, T., Zeigler, B.M., Dzierzak, E., and Speck, N.A. (2009). Runx1 is required for the endothelial to haematopoietic cell transition but not thereafter. Nature *457*, 887–891.

Ciau-Uitz, A., Walmsley, M., and Patient, R. (2000). Distinct origins of adult and embryonic blood in Xenopus. Cell *102*, 787–796.

Ciau-Uitz, A., Wang, L., Patient, R., and Liu, F. (2013). ETS transcription factors in hematopoietic stem cell development. Blood Cells Mol. Dis. *51*, 248–255.

Ciau-Uitz, A., Patient, R., and Medvinsky, A. (2016). Ontogeny of the Haematopoietic System. In Encyclopedia of Immunobiology, *Volume 1*, M.J.H. Ratcliffe, ed. *(Academic Press), pp. 1–14*.

Clements, W.K., and Traver, D. (2013). Signalling pathways that control vertebrate haematopoietic stem cell specification. Nat. Rev. Immunol. *13*, 336–348.

Corada, M., Orsenigo, F., Morini, M.F., Pitulescu, M.E., Bhat, G., Nyqvist, D., Breviario, F., Conti, V., Briot, A., Iruela-Arispe, M.L., et al. (2013). Sox17 is indispensable for acquisition and maintenance of arterial identity. Nat. Commun. *4*, 2609.

Croft, D., O'Kelly, G., Wu, G., Haw, R., Gillespie, M., Matthews, L., Caudy, M., Garapati, P., Gopinath, G., Jassal, B., et al. (2011). Reactome: a database of reactions, pathways and biological processes. Nucleic Acids Res. *39*, D691–D697.

Cruciat, C.M., and Niehrs, C. (2013). Secreted and transmembrane wnt inhibitors and activators. Cold Spring Harb. Perspect. Biol. *5*, a015081.

de Bruijn, M.F., Speck, N.A., Peeters, M.C., and Dzierzak, E. (2000). Definitive hematopoietic stem cells first develop within the major arterial regions of the mouse embryo. EMBO J. *19*, 2465–2474.

de Pater, E., Kaimakis, P., Vink, C.S., Yokomizo, T., Yamada-Inagawa, T., van der Linden, R., Kartalaei, P.S., Camper, S.A., Speck, N., and Dzierzak, E. (2013). Gata2 is required for HSC generation and survival. J. Exp. Med. *210*, 2843–2850.

Ditadi, A., Sturgeon, C.M., Tober, J., Awong, G., Kennedy, M., Yzaguirre, A.D., Azzola, L., Ng, E.S., Stanley, E.G., French, D.L., et al. (2015). Human definitive haemogenic endothelium and arterial vascular endothelium represent distinct lineages. Nat. Cell Biol. *17*, 580–591.

Ditadi, A., Sturgeon, C.M., and Keller, G. (2017). A view of human haematopoietic development from the Petri dish. Nat. Rev. Mol. Cell Biol. *18*, 56–67.

Dobin, A., Davis, C.A., Schlesinger, F., Drenkow, J., Zaleski, C., Jha, S., Batut, P., Chaisson, M., and Gingeras, T.R. (2013). STAR: ultrafast universal RNA-seq aligner. Bioinformatics *29*, 15–21.

Dodt, M., Roehr, J.T., Ahmed, R., and Dieterich, C. (2012). FLEXBAR-Flexible Barcode and Adapter Processing for Next-Generation Sequencing Platforms. Biology (Basel) *1*, 895–905.

Dubois, N.C., Adolphe, C., Ehninger, A., Wang, R.A., Robertson, E.J., and Trumpp, A. (2008). Placental rescue reveals a sole requirement for c-Myc in embryonic erythroblast survival and hematopoietic stem cell function. Development *135*, 2455–2465.

Easterbrook, J., Rybtsov, S., Gordon-Keylock, S., Ivanovs, A., Taoudi, S., Anderson, R.A., and Medvinsky, A. (2019). Analysis of the Spatiotemporal Development of Hematopoietic Stem and Progenitor Cells in the Early Human Embryo. Stem Cell Reports *12*, 1056–1068.

Efremova, M., Vento-Tormo, M., Teichmann, S.A., and Vento-Tormo, R. (2020). CellPhoneDB: inferring cell-cell communication from combined expression of multi-subunit ligand-receptor complexes. Nat. Protoc. *15*, 1484–1506.

Espín-Palazón, R., Stachura, D.L., Campbell, C.A., García-Moreno, D., Del Cid, N., Kim, A.D., Candel, S., Meseguer, J., Mulero, V., and Traver, D. (2014). Proinflammatory signaling regulates hematopoietic stem cell emergence. Cell *159*, 1070–1085.

Fitch, S.R., Kimber, G.M., Wilson, N.K., Parker, A., Mirshekar-Syahkal, B., Göttgens, B., Medvinsky, A., Dzierzak, E., and Ottersbach, K. (2012). Signaling from the sympathetic nervous system regulates hematopoietic stem cell emergence during embryogenesis. Cell Stem Cell *11*, 554–566.

Gama-Norton, L., Ferrando, E., Ruiz-Herguido, C., Liu, Z., Guiu, J., Islam, A.B., Lee, S.U., Yan, M., Guidos, C.J., López-Bigas, N., et al. (2015). Notch signal strength controls cell fate in the haemogenic endothelium. Nat. Commun. *6*, 8510.

Garcia-Porrero, J.A., Godin, I.E., and Dieterlen-Lièvre, F. (1995). Potential intraembryonic hemogenic sites at pre-liver stages in the mouse. Anat. Embryol. (Berl.) *192*, 425–435.

Goyama, S., Yamamoto, G., Shimabe, M., Sato, T., Ichikawa, M., Ogawa, S., Chiba, S., and Kurokawa, M. (2008). Evi-1 is a critical regulator for hematopoietic stem cells and transformed leukemic cells. Cell Stem Cell *3*, 207–220.

Hadland, B.K., Varnum-Finney, B., Poulos, M.G., Moon, R.T., Butler, J.M., Rafii, S., and Bernstein, I.D. (2015). Endothelium and NOTCH specify and amplify aorta-gonad-mesonephros-derived hematopoietic stem cells. J. Clin. Invest. *125*, 2032–2045.

Hautala, N., Tokola, H., Luodonpää, M., Puhakka, J., Romppanen, H., Vuolteenaho, O., and Ruskoaho, H. (2001). Pressure overload increases GATA4 binding activity via endothelin-1. Circulation *103*, 730–735.

Herzog, Y., Kalcheim, C., Kahane, N., Reshef, R., and Neufeld, G. (2001). Differential expression of neuropilin-1 and neuropilin-2 in arteries and veins. Mech. Dev. *109*, 115–119.

Ivanovs, A., Rybtsov, S., Welch, L., Anderson, R.A., Turner, M.L., and Medvinsky, A. (2011). Highly potent human hematopoietic stem cells first emerge in the intraembryonic aorta-gonad-mesonephros region. J. Exp. Med. *208*, 2417–2427.

Ivanovs, A., Rybtsov, S., Anderson, R.A., Turner, M.L., and Medvinsky, A. (2014). Identification of the niche and phenotype of the first human hematopoietic stem cells. Stem Cell Reports *2*, 449–456.

Jaffredo, T., Gautier, R., Eichmann, A., and Dieterlen-Lièvre, F. (1998). Intraaortic hemopoietic cells are derived from endothelial cells during ontogeny. Development *125*, 4575–4583.

Jokubaitis, V.J., Sinka, L., Driessen, R., Whitty, G., Haylock, D.N., Bertoncello, I., Smith, I., Péault, B., Tavian, M., and Simmons, P.J. (2008). Angiotensin-converting enzyme (CD143) marks hematopoietic stem cells in human embryonic, fetal, and adult hematopoietic tissues. Blood *111*, 4055–4063.

Kanehisa, M., and Goto, S. (2000). KEGG: kyoto encyclopedia of genes and genomes. Nucleic Acids Res. *28*, 27–30.

Kim, I., Saunders, T.L., and Morrison, S.J. (2007). Sox17 dependence distinguishes the transcriptional regulation of fetal from adult hematopoietic stem cells. Cell *130*, 470–483.

Kissa, K., and Herbomel, P. (2010). Blood stem cells emerge from aortic endothelium by a novel type of cell transition. Nature *464*, 112–115.

Kitta, K., Clément, S.A., Remeika, J., Blumberg, J.B., and Suzuki, Y.J. (2001). Endothelin-1 induces phosphorylation of GATA-4 transcription factor in the HL-1 atrial-muscle cell line. Biochem. J. *359*, 375–380.

La Manno, G., Soldatov, R., Zeisel, A., Braun, E., Hochgerner, H., Petukhov, V., Lidschreiber, K., Kastriti, M.E., Lönnerberg, P., Furlan, A., et al. (2018). RNA velocity of single cells. Nature *560*, 494–498.

Labastie, M.C., Cortés, F., Roméo, P.H., Dulac, C., and Péault, B. (1998). Molecular identity of hematopoietic precursor cells emerging in the human embryo. Blood *92*, 3624–3635.

Lapidot, T., and Kollet, O. (2002). The essential roles of the chemokine SDF-1 and its receptor CXCR4 in human stem cell homing and repopulation of transplanted immune-deficient NOD/SCID and NOD/SCID/B2m(null) mice. Leukemia *16*, 1992–2003.

Li, H., Handsaker, B., Wysoker, A., Fennell, T., Ruan, J., Homer, N., Marth, G., Abecasis, G., and Durbin, R.; 1000 Genome Project Data Processing Subgroup (2009). The Sequence Alignment/Map format and SAMtools. Bioinformatics *25*, 2078–2079.

Liberzon, A., Birger, C., Thorvaldsdóttir, H., Ghandi, M., Mesirov, J.P., and Tamayo, P. (2015). The Molecular Signatures Database (MSigDB) hallmark gene set collection. Cell Syst. *1*, 417–425.

Lin, H., Sangmal, M., Smith, M.J., Jr., and Young, D.B. (1993). Effect of endothelin-1 on glomerular hydraulic pressure and renin release in dogs. Hypertension *21*, 845–851.

Lindskog, H., Kim, Y.H., Jelin, E.B., Kong, Y., Guevara-Gallardo, S., Kim, T.N., and Wang, R.A. (2014). Molecular identification of venous progenitors in the dorsal aorta reveals an aortic origin for the cardinal vein in mammals. Development *141*, 1120–1128.

Lizama, C.O., Hawkins, J.S., Schmitt, C.E., Bos, F.L., Zape, J.P., Cautivo, K.M., Borges Pinto, H., Rhyner, A.M., Yu, H., Donohoe, M.E., et al. (2015). Repression of arterial genes in hemogenic endothelium is sufficient for haematopoietic fate acquisition. Nat. Commun. *6*, 7739.

Love, M.I., Huber, W., and Anders, S. (2014). Moderated estimation of fold change and dispersion for RNA-seq data with DESeq2. Genome Biol. *15*, 550.

Lund, S.A., Giachelli, C.M., and Scatena, M. (2009). The role of osteopontin in inflammatory processes. J. Cell Commun. Signal. *3*, 311–322.

Luo, X., Ruhland, M.K., Pazolli, E., Lind, A.C., and Stewart, S.A. (2011). Osteopontin stimulates preneoplastic cellular proliferation through activation of the MAPK pathway. Mol. Cancer Res. *9*, 1018–1029.

McGarvey, A.C., Rybtsov, S., Souilhol, C., Tamagno, S., Rice, R., Hills, D., Godwin, D., Rice, D., Tomlinson, S.R., and Medvinsky, A. (2017). A molecular roadmap of the AGM region reveals BMPER as a novel regulator of HSC maturation. J. Exp. Med. *214*, 3731–3751.

McInnes, L., Healy, J., and Melville, J. (2018). UMAP: Uniform Manifold Approximation and Projection for Dimension Reduction. arXiv, arXiv:1802.03426 https://arxiv.org/abs/1802.03426.

Medvinsky, A., and Dzierzak, E. (1996). Definitive hematopoiesis is autonomously initiated by the AGM region. Cell *86*, 897–906.

Medvinsky, A.L., Gan, O.I., Semenova, M.L., and Samoylina, N.L. (1996). Development of day-8 colony-forming unit-spleen hematopoietic progenitors during early murine embryogenesis: spatial and temporal mapping. Blood *87*, 557–566.

Medvinsky, A., Rybtsov, S., and Taoudi, S. (2011). Embryonic origin of the adult hematopoietic system: advances and questions. Development *138*, 1017–1031.

Mikkola, H.K., Fujiwara, Y., Schlaeger, T.M., Traver, D., and Orkin, S.H. (2003). Expression of CD41 marks the initiation of definitive hematopoiesis in the mouse embryo. Blood *101*, 508–516.

Mohamed, I.A., Gadeau, A.P., Fliegel, L., Lopaschuk, G., Mlih, M., Abdulrahman, N., Fillmore, N., and Mraiche, F. (2015). Na+/H+ exchanger isoform 1-induced osteopontin expression facilitates cardiomyocyte hypertrophy. PLoS ONE *10*, e0123318.

Mucenski, M.L., McLain, K., Kier, A.B., Swerdlow, S.H., Schreiner, C.M., Miller, T.A., Pietryga, D.W., Scott, W.J., Jr., and Potter, S.S. (1991). A functional c-myb gene is required for normal murine fetal hepatic hematopoiesis. Cell *65*, 677–689.

Nataf, V., Lecoin, L., Eichmann, A., and Le Douarin, N.M. (1996). Endothelin-B receptor is expressed by neural crest cells in the avian embryo. Proc. Natl. Acad. Sci. USA *93*, 9645–9650.

Ng, E.S., Azzola, L., Bruveris, F.F., Calvanese, V., Phipson, B., Vlahos, K., Hirst, C., Jokubaitis, V.J., Yu, Q.C., Maksimovic, J., et al. (2016). Differentiation of human embryonic stem cells to HOXA$^+$ hemogenic vasculature that resembles the aorta-gonad-mesonephros. Nat. Biotechnol. *34*, 1168–1179.

Nichterwitz, S., Chen, G., Benitez, J.A., Yilmaz, M., Storvall, H., Cao, M., Sandberg, R., Deng, Q., and Hedlund, E. (2016). Laser capture microscopy coupled with Smart-seq2 for precise spatial transcriptomic profiling. Nat. Commun. *7*, 12139.

Nilsson, S.K., Johnston, H.M., Whitty, G.A., Williams, B., Webb, R.J., Denhardt, D.T., Bertoncello, I., Bendall, L.J., Simmons, P.J., and Haylock, D.N. (2005). Osteopontin, a key component of the hematopoietic stem cell niche and regulator of primitive hematopoietic progenitor cells. Blood *106*, 1232–1239.

North, T.E., Goessling, W., Peeters, M., Li, P., Ceol, C., Lord, A.M., Weber, G.J., Harris, J., Cutting, C.C., Huang, P., et al. (2009). Hematopoietic stem cell development is dependent on blood flow. Cell *137*, 736–748.

Nottingham, W.T., Jarratt, A., Burgess, M., Speck, C.L., Cheng, J.F., Prabhakar, S., Rubin, E.M., Li, P.S., Sloane-Stanley, J., Kong-A-San, J., and de Bruijn, M.F. (2007). Runx1-mediated hematopoietic stem-cell emergence is controlled by a Gata/Ets/SCL-regulated enhancer. Blood *110*, 4188–4197.

Oatley, M., Bölükbası, O.V., Svensson, V., Shvartsman, M., Ganter, K., Zirngibl, K., Pavlovich, P.V., Milchevskaya, V., Foteva, V., Natarajan, K.N., et al. (2020). Single-cell transcriptomics identifies CD44 as a marker and regulator of endothelial to haematopoietic transition. Nat. Commun. *11*, 586.

Oka, T., Maillet, M., Watt, A.J., Schwartz, R.J., Aronow, B.J., Duncan, S.A., and Molkentin, J.D. (2006). Cardiac-specific deletion of Gata4 reveals its requirement for hypertrophy, compensation, and myocyte viability. Circ. Res. *98*, 837–845.

Park, M.A., Kumar, A., Jung, H.S., Uenishi, G., Moskvin, O.V., Thomson, J.A., and Slukvin, I.I. (2018). Activation of the Arterial Program Drives Development of Definitive Hemogenic Endothelium with Lymphoid Potential. Cell Rep. *23*, 2467–2481.

Peeters, M., Ottersbach, K., Bollerot, K., Orelio, C., de Bruijn, M., Wijgerde, M., and Dzierzak, E. (2009). Ventral embryonic tissues and Hedgehog proteins induce early AGM hematopoietic stem cell development. Development *136*, 2613–2621.

Pellegrini, M., Pantano, S., Lucchini, F., Fumi, M., and Forabosco, A. (1997). Emx2 developmental expression in the primordia of the reproductive and excretory systems. Anat. Embryol. (Berl.) *196*, 427–433.

Pendeville, H., Winandy, M., Manfroid, I., Nivelles, O., Motte, P., Pasque, V., Peers, B., Struman, I., Martial, J.A., and Voz, M.L. (2008). Zebrafish Sox7 and Sox18 function together to control arterial-venous identity. Dev. Biol. *317*, 405–416.

Picelli, S., Faridani, O.R., Björklund, A.K., Winberg, G., Sagasser, S., and Sandberg, R. (2014). Full-length RNA-seq from single cells using Smart-seq2. Nat. Protoc. *9*, 171–181.

Quinlan, A.R., and Hall, I.M. (2010). BEDTools: a flexible suite of utilities for comparing genomic features. Bioinformatics *26*, 841–842.

Richard, C., Drevon, C., Canto, P.Y., Villain, G., Bollérot, K., Lempereur, A., Teillet, M.A., Vincent, C., Rosselló Castillo, C., Torres, M., et al. (2013). Endothelio-mesenchymal interaction controls runx1 expression and modulates the notch pathway to initiate aortic hematopoiesis. Dev. Cell *24*, 600–611.

Rönn, R.E., Guibentif, C., Moraghebi, R., Chaves, P., Saxena, S., Garcia, B., and Woods, N.B. (2015). Retinoic acid regulates hematopoietic development from human pluripotent stem cells. Stem Cell Reports *4*, 269–281.

Rossi, G.P., Sacchetto, A., Cesari, M., and Pessina, A.C. (1999). Interactions between endothelin-1 and the renin-angiotensin-aldosterone system. Cardiovasc. Res. *43*, 300–307.

Rybtsov, S., Sobiesiak, M., Taoudi, S., Souilhol, C., Senserrich, J., Liakhovitskaia, A., Ivanovs, A., Frampton, J., Zhao, S., and Medvinsky, A. (2011). Hierarchical organization and early hematopoietic specification of the developing HSC lineage in the AGM region. J. Exp. Med. *208*, 1305–1315.

Rybtsov, S., Batsivari, A., Bilotkach, K., Paruzina, D., Senserrich, J., Nerushev, O., and Medvinsky, A. (2014). Tracing the origin of the HSC hierarchy reveals an SCF-dependent, IL-3-independent CD43(-) embryonic precursor. Stem Cell Reports *3*, 489–501.

Schindelin, J., Arganda-Carreras, I., Frise, E., Kaynig, V., Longair, M., Pietzsch, T., Preibisch, S., Rueden, C., Saalfeld, S., Schmid, B., et al. (2012). Fiji: an open-source platform for biological-image analysis. Nat. Methods *9*, 676–682.

Shah, B.H., and Catt, K.J. (2003). A central role of EGF receptor transactivation in angiotensin II -induced cardiac hypertrophy. Trends Pharmacol. Sci. *24*, 239–244.

Shannon, P., Markiel, A., Ozier, O., Baliga, N.S., Wang, J.T., Ramage, D., Amin, N., Schwikowski, B., and Ideker, T. (2003). Cytoscape: a software environment for integrated models of biomolecular interaction networks. Genome Res. *13*, 2498–2504.

Sinka, L., Biasch, K., Khazaal, I., Péault, B., and Tavian, M. (2012). Angiotensin-converting enzyme (CD143) specifies emerging lympho-hematopoietic progenitors in the human embryo. Blood *119*, 3712–3723.

Slukvin, I.I. (2013). Deciphering the hierarchy of angiohematopoietic progenitors from human pluripotent stem cells. Cell Cycle *12*, 720–727.

Souilhol, C., Gonneau, C., Lendinez, J.G., Batsivari, A., Rybtsov, S., Wilson, H., Morgado-Palacin, L., Hills, D., Taoudi, S., Antonchuk, J., et al. (2016a). Inductive interactions mediated by interplay of asymmetric signalling underlie development of adult haematopoietic stem cells. Nat. Commun. *7*, 10784.

Souilhol, C., Lendinez, J.G., Rybtsov, S., Murphy, F., Wilson, H., Hills, D., Batsivari, A., Binagui-Casas, A., McGarvey, A.C., MacDonald, H.R., et al. (2016b). Developing HSCs become Notch independent by the end of maturation in the AGM region. Blood *128*, 1567–1577.

Stier, S., Ko, Y., Forkert, R., Lutz, C., Neuhaus, T., Grünewald, E., Cheng, T., Dombkowski, D., Calvi, L.M., Rittling, S.R., and Scadden, D.T. (2005). Osteopontin is a hematopoietic stem cell niche component that negatively regulates stem cell pool size. J. Exp. Med. *201*, 1781–1791.

Subramanian, A., Tamayo, P., Mootha, V.K., Mukherjee, S., Ebert, B.L., Gillette, M.A., Paulovich, A., Pomeroy, S.L., Golub, T.R., Lander, E.S., and Mesirov, J.P. (2005). Gene set enrichment analysis: a knowledge-based approach for interpreting genome-wide expression profiles. Proc. Natl. Acad. Sci. USA *102*, 15545–15550.

Subramanian, A., Kuehn, H., Gould, J., Tamayo, P., and Mesirov, J.P. (2007). GSEA-P: a desktop application for Gene Set Enrichment Analysis. Bioinformatics *23*, 3251–3253.

Sugiyama, T., Kohara, H., Noda, M., and Nagasawa, T. (2006). Maintenance of the hematopoietic stem cell pool by CXCL12-CXCR4 chemokine signaling in bone marrow stromal cell niches. Immunity *25*, 977–988.

Szklarczyk, D., Franceschini, A., Wyder, S., Forslund, K., Heller, D., Huerta-Cepas, J., Simonovic, M., Roth, A., Santos, A., Tsafou, K.P., et al. (2015). STRING v10: protein-protein interaction networks, integrated over the tree of life. Nucleic Acids Res. *43*, D447–D452.

Szklarczyk, D., Gable, A.L., Lyon, D., Junge, A., Wyder, S., Huerta-Cepas, J., Simonovic, M., Doncheva, N.T., Morris, J.H., Bork, P., et al. (2019). STRING v11: protein-protein association networks with increased coverage, supporting functional discovery in genome-wide experimental datasets. Nucleic Acids Res. *47* (D1), D607–D613.

Tang, Y., Bai, H., Urs, S., Wang, Z., and Liaw, L. (2013). Notch1 activation in embryonic VE-cadherin populations selectively blocks hematopoietic stem cell generation and fetal liver hematopoiesis. Transgenic Res. *22*, 403–410.

Taoudi, S., and Medvinsky, A. (2007). Functional identification of the hematopoietic stem cell niche in the ventral domain of the embryonic dorsal aorta. Proc. Natl. Acad. Sci. USA *104*, 9399–9403.

Taoudi, S., Morrison, A.M., Inoue, H., Gribi, R., Ure, J., and Medvinsky, A. (2005). Progressive divergence of definitive haematopoietic stem cells from the endothelial compartment does not depend on contact with the foetal liver. Development *132*, 4179–4191.

Taoudi, S., Gonneau, C., Moore, K., Sheridan, J.M., Blackburn, C.C., Taylor, E., and Medvinsky, A. (2008). Extensive hematopoietic stem cell generation in the AGM region via maturation of VE-cadherin+CD45+ pre-definitive HSCs. Cell Stem Cell *3*, 99–108.

Tavian, M., Coulombel, L., Luton, D., Clemente, H.S., Dieterlen-Lièvre, F., and Péault, B. (1996). Aorta-associated CD34+ hematopoietic cells in the early human embryo. Blood *87*, 67–72.

Tavian, M., Hallais, M.F., and Péault, B. (1999). Emergence of intraembryonic hematopoietic precursors in the pre-liver human embryo. Development *126*, 793–803.

Taylor, E., Taoudi, S., and Medvinsky, A. (2010). Hematopoietic stem cell activity in the aorta-gonad-mesonephros region enhances after mid-day 11 of mouse development. Int. J. Dev. Biol. *54*, 1055–1060.

Thambyrajah, R., Mazan, M., Patel, R., Moignard, V., Stefanska, M., Marinopoulou, E., Li, Y., Lancrin, C., Clapes, T., Möröy, T., et al. (2016). GFI1 proteins orchestrate the emergence of haematopoietic stem cells through recruitment of LSD1. Nat. Cell Biol. *18*, 21–32.

The Gene Ontology Consortium (2019). The Gene Ontology Resource: 20 years and still GOing strong. Nucleic Acids Res. *47*, D330–D338.

Traag, V.A., Waltman, L., and van Eck, N.J. (2019). From Louvain to Leiden: guaranteeing well-connected communities. Sci. Rep. *9*, 5233.

Ulloa, L., and Tabibzadeh, S. (2001). Lefty inhibits receptor-regulated Smad phosphorylation induced by the activated transforming growth factor-beta receptor. J. Biol. Chem. *276*, 21397–21404.

Villa, N., Walker, L., Lindsell, C.E., Gasson, J., Iruela-Arispe, M.L., and Weinmaster, G. (2001). Vascular expression of Notch pathway receptors and ligands is restricted to arterial vessels. Mech. Dev. *108*, 161–164.

Wang, L., Zhang, P., Wei, Y., Gao, Y., Patient, R., and Liu, F. (2011). A blood flow-dependent klf2a-NO signaling cascade is required for stabilization of hematopoietic stem cell programming in zebrafish embryos. Blood *118*, 4102–4110.

Wickham, H. (2019). ggplot2: Elegant Graphics for Data Analysis (Springer).

Wilson, A., Murphy, M.J., Oskarsson, T., Kaloulis, K., Bettess, M.D., Oser, G.M., Pasche, A.C., Knabenhans, C., Macdonald, H.R., and Trumpp, A. (2004). c-Myc controls the balance between hematopoietic stem cell self-renewal and differentiation. Genes Dev. *18*, 2747–2763.

Wolf, F.A., Angerer, P., and Theis, F.J. (2018). SCANPY: large-scale single-cell gene expression data analysis. Genome Biol. *19*, 15.

Wolf, F.A., Hamey, F.K., Plass, M., Solana, J., Dahlin, J.S., Göttgens, B., Rajewsky, N., Simon, L., and Theis, F.J. (2019). PAGA: graph abstraction reconciles clustering with trajectory inference through a topology preserving map of single cells. Genome Biol. *20*, 59.

Wright, D.E., Bowman, E.P., Wagers, A.J., Butcher, E.C., and Weissman, I.L. (2002). Hematopoietic stem cells are uniquely selective in their migratory response to chemokines. J. Exp. Med. *195*, 1145–1154.

Yamamizu, K., Matsunaga, T., Uosaki, H., Fukushima, H., Katayama, S., Hiraoka-Kanie, M., Mitani, K., and Yamashita, J.K. (2010). Convergence of Notch and beta-catenin signaling induces arterial fate in vascular progenitors. J. Cell Biol. *189*, 325–338.

Yokomizo, T., and Dzierzak, E. (2010). Three-dimensional cartography of hematopoietic clusters in the vasculature of whole mouse embryos. Development *137*, 3651–3661.

Yokomizo, T., Watanabe, N., Umemoto, T., Matsuo, J., Harai, R., Kihara, Y., Nakamura, E., Tada, N., Sato, T., Takaku, T., et al. (2019). Hlf marks the developmental pathway for hematopoietic stem cells but not for erythro-myeloid progenitors. J. Exp. Med. *216*, 1599–1614.

You, L.R., Lin, F.J., Lee, C.T., DeMayo, F.J., Tsai, M.J., and Tsai, S.Y. (2005). Suppression of Notch signalling by the COUP-TFII transcription factor regulates vein identity. Nature *435*, 98–104.

Zeng, Y., He, J., Bai, Z., Li, Z., Gong, Y., Liu, C., Ni, Y., Du, J., Ma, C., Bian, L., et al. (2019). Tracing the first hematopoietic stem cell generation in human embryo by single-cell RNA sequencing. Cell Res. *29*, 881–894.

Zhou, F., Li, X., Wang, W., Zhu, P., Zhou, J., He, W., Ding, M., Xiong, F., Zheng, X., Li, Z., et al. (2016). Tracing haematopoietic stem cell formation at single-cell resolution. Nature *533*, 487–492.

Zovein, A.C., Hofmann, J.J., Lynch, M., French, W.J., Turlo, K.A., Yang, Y., Becker, M.S., Zanetta, L., Dejana, E., Gasson, J.C., et al. (2008). Fate tracing reveals the endothelial origin of hematopoietic stem cells. Cell Stem Cell *3*, 625–636.

STAR★METHODS

KEY RESOURCES TABLE

REAGENT or RESOURCE	SOURCE	IDENTIFIER
Antibodies		
Mouse monoclonal anti-CD144	BD Biosciences	Cat# 555661, RRID:AB_396015
Sheep polyclonal anti-CD31/PECAM1	R and D Systems	Cat# AF806, RRID:AB_355617
Rabbit monoclonal anti-RUNX1	Abcam	Cat# ab92336, RRID:AB_1580795
Mouse monoclonal anti-CD45	BD Biosciences	Cat# 555480, RRID:AB_395872
Rabbit monoclonal anti-Renin	Abcam	Cat#ab212196, Clone:EPR20693
Mouse monoclonal anti-Endothelin 1	Abcam	Cat# ab2786, RRID:AB_303299
Mouse monoclonal anti- Endothelin A Receptor	R and D Systems	Cat# MAB65381, Clone:485709
Rabbit polyclonal anti-Endothelin B Receptor	Abcam	Cat# ab117529, RRID:AB_10902070
Rat monoclonal anti-CD31/PECAM1	Biolegend	Cat#102501, RRID:AB_312908
Goat polyclonal anti-c-Kit	R and D Systems	Cat# AF1356, RRID:AB_354750
Rabbit polyclonal anti-Endothelin 1	Abcam	Cat# ab117757, RRID:AB_10901366
Donkey polyclonal anti-Sheep IgG NL557	R and D Systems	Cat# NL010, RRID:AB_884220
Goat polyclonal anti-Mouse IgG (H+L) AF488	Thermo Fisher Scientific	Cat# A-11001, RRID:AB_2534069
Donkey polyclonal anti-Rabbit IgG (H+L) AF647	Abcam	Cat# ab150075, RRID:AB_2752244
Goat polyclonal anti-Rat IgG (H+L) AF546	Thermo Fisher Scientific	Cat# A-11081, RRID:AB_2534125
Donkey polyclonal anti-Goat IgG (H+L) AF488	Thermo Fisher Scientific	Cat# A-11055, RRID:AB_2534102
Mouse anti-CD45.1, V450 Conjugated, Clone A20	BD Biosciences	Cat# 560520, RRID:AB_1727490
Mouse anti-CD45.1 APC Conjugated, Clone A20	Thermo Fisher Scientific	Cat# 17-0453-82, RRID:AB_469398
Mouse anti-CD45.2 V500 Conjugated, Clone 104	BD Biosciences	Cat#562129, RRID:AB_10897142
Mouse anti-CD45.2, PE Conjugated, Clone 104	Thermo Fisher Scientific	Cat# 12-0454-82, RRID:AB_465678
Rat anti-CD45R, APC-Cy7 Conjugated, Clone RA3-6B2	BD Biosciences	Cat# 552094, RRID:AB_394335
Armenian Hamster anti-CD11c, PE/Cy7 Conjugated, Clone N418	BioLegend	Cat# 117317, RRID:AB_493569
Rat anti-TER119, FITC Conjugated	Thermo Fisher Scientific	Cat# 11-5921-82, RRID:AB_465311
Rat anti-Ly-6G/Ly-6C, PE Conjugated, Clone RB6-8C5	Thermo Fisher Scientific	Cat# 12-5931-81, RRID:AB_466044
Rat anti-CD335, BV711 Conjugated, Clone 29A1.4	BioLegend	Cat# 137621, RRID:AB_2563289
Armenian Hamster anti-CD3e, APC Conjugated, Clone 145-2C11	Thermo Fisher Scientific	Cat# MA1-10186, RRID:AB_11153519
Rat anti-CD4, APC Conjugated, Clone GK1.5	Thermo Fisher Scientific	Cat# MA1-10218, RRID:AB_11152647
Mouse Anti-Human CD144, PE Conjugated, Clone TEA 1/31	Beckman Coulter	Cat# A07481
Mouse Anti-Human CD45 Monoclonal Antibody, V450 Conjugated	BD Biosciences	Cat# 560368, RRID: AB_1645574

(*Continued on next page*)

Continued

REAGENT or RESOURCE	SOURCE	IDENTIFIER
Mouse Anti-CD235a Monoclonal Antibody, Allophycocyanin Conjugated, Clone GA-R2 (HIR2)	BD Biosciences	Cat# 551336, RRID:AB_398499
Rat anti-CD8a, BV711 Conjugated, Clone 53-6.7	BioLegend	Cat# 100747, RRID:AB_11219594
Chemicals, Peptides, and Recombinant Proteins		
Tissue-Tek® O.C.T. Compound	VWR	Cat# 25608-930
Hematoxylin Solution, Mayer's	Sigma-Aldrich	Cat# MHS32-1L
Eosin Y Solution	Sigma-Aldrich	Cat# HT110216
Diethyl pyrocarbonate	Sigma-Aldrich	Cat# 40718
RNaseAWAY ®	Sigma-Aldrich	Cat# 83931
Recombinant RNase Inhibitor	Takara	Cat# 2313B
10mM dNTP mix	Thermo Fisher Scientific	Cat# 18427013
SuperScript IV Reverse Transcriptase	Thermo Fisher Scientific	Cat# 18090010
KAPA HiFi HotStart ReadyMix	Roche	Cat# KK2601
Nuclease-Free Water (not DEPC-Treated)	Thermo Fisher Scientific	Cat# AM9939
ProLong Gold Antifade Mountant	Thermo Fisher Scientific	Cat# P36930
DAPI Solution	Thermo Fisher Scientific	Cat# 62248
Opal 520	Perkin Elmer	Cat# FP1487001KT
Opal 570	Perkin Elmer	Cat# FP1488001KT
Opal 690	Perkin Elmer	Cat# FP1497001KT
Collagenase/Dispase	Roche	Cat# 10269638001
DNase I recombinant	Roche	Cat# 4716728001
IMDM	Thermo Fisher Scientific	Cat# 21980032
DPBS, calcium, magnesium	Thermo Fisher Scientific	Cat# 14040091
HyClone Fetal Bovine Serum, South American Origin	Fisher Scientific	Cat# 10309433
Human Endothelin-1	Sigma-Aldrich	Cat# E7764
Methocult H4034 Optimum	StemCell Technologies, Inc.	N/A
MethoCult GF M3434	StemCell Technologies, Inc.	N/A
Mouse recombinant Endothelin 1	LSBio	Cat# LS-G26630-10
β-Endothelin mouse (Endothelin 2)	Sigma-Aldrich	Cat# SCP0259
Mouse recombinant Renin	Sigma-Aldrich	Cat# SRP6266
mTeSR1 Complete Kit – GMP	StemCell Technologies, Inc.	Cat# 85850
ACCUTASE	StemCell Technologies, Inc.	Cat# 07922
STEMdiff APEL2	StemCell Technologies, Inc.	Cat# 05270
Recombinant Human BMP-4 Protein	R and D Systems	Cat# 314-BP
Recombinant Human/Mouse/Rat Activin A Protein	R and D Systems	Cat# 338-AC
Human VEGF 165	Peprotech	Cat# 100-20
Human EPO	Peprotech	Cat# 100-64
Human FGF-basic	Peprotech	Cat# 100-18B
Human IGF-II	Peprotech	Cat# 100-12
Human IL-3	Peprotech	Cat# 200-03
Human IL-6	Peprotech	Cat# 200-06
Human SCF	Peprotech	Cat# 300-07
Human TPO	Peprotech	Cat# 300-18

(*Continued on next page*)

Continued

REAGENT or RESOURCE	SOURCE	IDENTIFIER
Y-27632 dihydrochloride	R and D Systems	Cat# 1254
SB431542	Cayman Chemicals	Cat# 13031
Chir99021	Tocris Biosciences	Cat# 4423
Critical Commercial Assays		
PicoPure RNA Isolation Kit	Thermo Fisher Scientific	Cat# KIT0204
SMARTer Stranded Total RNA-Seq Kit v2 - Pico Input Mammalian	Takara	Cat# 634412
CD34 MicroBead Kit, human	Miltenyi Biotec	Cat# 130-046-702
RNAScope Multiplex Fluorescent Reagent Kit v2	ACD, Biotechne	Cat# 323100
Deposited Data		
RNA-Seq Raw Data for LCM-Seq, Bulk and Single-cell datasets	This paper	GEO: GSE151877
Experimental Models: Cell Lines		
ES Cells H9		N/A
OP9 Cells		N/A
Experimental Models: Organisms/Strains		
C5BL/6(CD45.2/2) mice		N/A
C57BL/6 CD45.1/2		N/A
Oligonucleotides		
oligodT (5′-AAGCAGTGGTATCAACGCA GAGTACTTTTTTTTTTTTTTT TTTTTTTTTTTTTTTVN-3′)	IDT	N/A
TSO-LNA-oligo (5′- AAGCAGTGGTATCAA CGCAGAGTACATrGrG+G –3′)	Exiqon	N/A
ISPCR primers (5′ - AAG CAG TGG TAT CAA CGC AGA GT – 3′)	IDT	N/A
Software and Algorithms		
R (R-3.2.3 – R-3.6.1)	The R Foundation	https://www.r-project.org
Cellranger (v2.1.0)	10X Genomics	https://support.10xgenomics.com
FastQC	Andrews, 2010	N/A
Flexbar	Dodt et al., 2012	N/A
STAR	Dobin et al., 2013	N/A
SAMtools	Li et al., 2009	N/A
Multicov, BEDtools	Quinlan and Hall, 2010	N/A
DESeq2	Love, Huber and Anders, 2014	N/A
ggplot2	Wickham, 2019	https://ggplot2.tidyverse.org/
EnhancedVolcano		https://github.com/kevinblighe/EnhancedVolcano
GSEA software	Subramanian et al., 2005, 2007; Broad Institute	N/A
String-DB	Szklarczyk et al., 2019	https://string-db.org/
Cytoscape software	Shannon et al., 2003	N/A
EnrichmentMap		https://www.baderlab.org/Software/EnrichmentMap
CellPhoneDB v2.0	Efremova et al., 2020	N/A
Loompy		https://github.com/linnarsson-lab/loompy
SCANPY	Wolf et al, 2018	N/A
Partition-based graph abstraction (PAGA)	Wolf et al., 2019	N/A
scVelo		https://github.com/theislab/scvelo

(*Continued on next page*)

Continued		
REAGENT or RESOURCE	SOURCE	IDENTIFIER
Other		
RNAScope Probe: Hs-EDN1	ACD, Biotechne	Cat# 459381
RNAScope Probe: Hs-EDNRA-C2	ACD, Biotechne	Cat# 443661-C2
RNAScope Probe: Hs-EDNRB-C3	ACD, Biotechne	Cat# 528301-C3
RNAScope Probe: Hs-REN-C3	ACD, Biotechne	Cat# 401921
RNAScope Probe: Hs-CDH5-C2	ACD, Biotechne	Cat# 437451-C2

RESOURCE AVAILABILITY

Lead Contact

Further information and requests for resources and reagents should be directed to and will be fulfilled by the Lead Contact, Alexander Medvinsky (A.Medvinsky@ed.ac.uk).

Materials Availability

This study did not generate new unique reagents.

Data and Code Availability

The accession number for the RNA-Sequencing raw data reported in this paper is GEO: GSE151877.

EXPERIMENTAL MODEL AND SUBJECT DETAILS

Human embryonic material

Human embryonic samples of Carnegie Stages 15 – 17 were provided by the MRC Centre for Reproductive Health and by the Joint MRC / Wellcome (MR/R006237/1) Human Developmental Biology Resource (https://www.hdbr.org/). This study was approved by the Lothian Research Ethics Committee. The embryos were obtained immediately after elective termination of pregnancy for which each patient gave informed consent in writing. Embryos were either used immediately as fresh tissue or flash frozen in Optimal Cutting Temperature (OCT) compound and stored at −80°C. Human embryo karyotypes: CS17 N2-3 (LCM-Seq1): 46,XY; CS16 N1-N3 (LCM-Seq2) + CS15 (FACS sorted): rsa(13,15,16,18,21,22)X2,(X,Y)X1; CS17 N1 (LCM-Seq1) + CS16 (FACS sorted) + 2x CS16 (Single-cell RNA-Seq): Unknown.

Mouse models

All mouse experiments were performed under a Project License granted by the Home Office (UK), University of Edinburgh Ethical Review Committee, and conducted in accordance with local guidelines. Animals were housed within the University of Edinburgh adhering to the Animals Scientific Procedures Act, UK, 1986. All mouse experiments were carried out by researchers with a personal license granted by the Home Office. Mice were kept in stable light cycling conditions (14 hours light and 10 hours dark) with a regular supply of chow food and water. Embryos were used from C57BL/6 (CD45.2/2) (Jackson Laboratories) mice and C57BL/6-Ly5.1 (CD45.1) mice were used as hosts for transplantation experiments.

To obtain embryos of the correct stage paired matings were set up and the morning of discovery of a vaginal plug is considered embryonic day (E) 0.5. The embryos were collected at E9.5. Following schedule 1 culling of the pregnant dam, the uterine horns were removed and the embryos dissected out of the extra-embryonic tissues including the yolk-sac and the amniotic sac. The embryos were then more accurately staged by counting the somite pairs (SPs) (E9.5 = 25 −29 SPs). The caudal parts were taken which includes everything below the heart.

Cell lines

The H9 human embryonic stem (ES) cell line was used in ES differentiation experiments and maintained in mTeSR1 (StemCell Technologies, Inc.) on Biolaminin 521 (BioLamina) coated wells at 37°C and 95% humidity in an atmosphere of 5% CO2. Media was changed daily and cells were passaged every 4-5 days using 1x Accutase® (Sigma-Aldrich) for dissociation.

OP9 cells were used in co-aggregates with mouse E9.5 primary cells. They were maintained in Iscove's modified Dulbecco's medium (IMDM, Invitrogen), 20% Foetal Calf Serum (FCS, GIBCO), L-glutamine (4 mM) and penicillin/streptomycin (P/S, GIBCO) (50 U/ml) at 37°C and 95% humidity in an atmosphere of 5% CO2.

METHOD DETAILS

Laser Capture Microdissection

Human embryos embedded in OCT stored at −80°C were equilibrated to −24°C and sectioned in a caudal-to-rostral direction using a cryotome FSE cryostat (Thermo Scientific). Frequent checks under the microscope verified the level reached along the

rostral-caudal axis as defined by anatomical landmarks. Once the appropriate level had been reached cryosections were transferred onto nuclease-free polyethylene napthalate (PEN) membrane slides (Zeiss). At intervals, a sister section would be transferred to a SuperFrost slide for future validation of ventral IAHCs by immunohistochemical analysis. Sections were stained using a rapid Haematoxylin and Eosin staining protocol - 3 minutes (min) in 70% ethanol, 1 min H_2O, 4 min Mayer's Haematoxylin Solution (Sigma-Aldrich), 2 min tap H_2O, 15 s Eosin Y (Sigma-Aldrich), 1 min 70% ethanol, 1 min 90% ethanol, 3 mins 100% ethanol. All H_2O was treated with diethyl pyrocarbonate (DEPC) and all reagents were pre-cooled in ice except 100% ethanol which was room temperature.

The laser capture microscope used was the PALM microbeam (Zeiss). The microscope and surrounding area were sprayed down with RNaseAWAY ® (Sigma-Aldrich). Sections were viewed and microdissected in brightfield using a 10X objective. The microdissected regions were collected into the caps of AdhesiveCap 500 opaque (Zeiss) 500μl PCR tubes. 15μl lysis buffer (0.2% Triton X-100 (Sigma-Aldrich), 2U/μl RNase inhibitor (Takara), Phosphate Buffer Solution (PBS; Nichterwitz et al., 2016) was added directly on top of the dissected tissue in the tube caps and the tubes closes in an inverted position.

Human embryo cell sorting

The dorsal aortas were dissected from human embryos CS15-16 (N = 2) then further bisected into ventral and dorsal portions (AoV and AoD respectively). Tissues were dissociated into single cells in 1mg/ml Collagenase-Dispase (Roche) and 0.12 mg/ml of DNase I (Roche) for 35 min in a 37°C rotating water bath and stained with conjugated antibodies anti-human VE-Cadherin-PE (Beckman Coulter, 5μg/ml), anti-human CD45 –v450 (Clone: HI30, BioLegend, RRID:AB_1645574, 6μg/ml) and anti-human CD235A – APC (BD Bioscience, RRID:AB_398499, 0.2μg/ml) for 1 hour at 4°C. Dead cells and erythroid cells were excluded by 7AAD and CD235A staining respectively. VC+CD45-, VC+CD45+ and VC-CD45+ populations were sorted using a FACS Aria-II (BD Bioscience) into Eppendorf tubes containing RLT buffer from the RNeasy Micro kit ready for RNA purification. Data acquisition and analysis was performed using FlowJo software (Tree Star).

For the 10X single cell sequencing, following AoV cell dissociation, CD34+ cells were isolated using the human CD34 MicroBead Kit and LS MACS Columns (both Miltenyi Biotec). This procedure was carried out as per the manufacturer's guidelines.

RNA-Seq Library Preparation

The previously published LCM-Seq protocol (Picelli et al., 2014) was used for RNA-Seq library preparation from LCM material in lysis buffer. LCM caps were vortexed for 15 s and spun in a tabletop centrifuge (8000 g) for 5 min. 5 μL lysate was added to 2 μL 10mM dNTP mix (Thermo Fisher) and 1 μL 10 μM oligodT (5′-AAGCAGTGGTATCAACGCAGAGTACTTTTTTTTTTTTTTTTTTTTTTTTTTTTTTVN-3′, (IDT)). This was vortexed briefly and spun in a microcentrifuge for 30 s (700 g) then incubated at 72°C for 3 min and immediately snap cooled on ice. To each reaction, 2 μl SSRTIV 5 × buffer, 0.5 μl 100 mM DTT, 0.5 μl 200 U μl−1 SSRTIV (all Thermo Fisher), 2 μl 5 M betaine (Sigma-Aldrich), 0.1 μl 1 M MgCl2 (Sigma-Aldrich), 0.25 μl 40 U μl−1 RNase inhibitor (Takara) and 0.1 μl 100 μM TSO-LNA-oligo 5′- AAGCAGTGGTATCAACGCAGAGTACATrGrG+G −3′, Exiqon) was added. The reverse transcription reaction was performed in a thermal heat cycler with the following conditions; 90min 42°C, 10 cycles of (2 min 50°C, 2 min 42°C) and 15 s 70°C. For the amplification reaction 12.5 μl 2X KAPA HiFI Hotstart Mix (KAPA Biosystems), 0.2 μl 10 μM ISPCR primers (IDT, 5′ - AAG CAG TGG TAT CAA CGC AGA GT – 3′) and 2.3 μl nuclease-free H_2O (Invitrogen) was added to each reaction. This was heat cycled as follows: 3 min 98°C, 18 cycles of (20 s 98°C, 15 s 67°C, 6 min 72°C) and 5 min 72°C. After bead purification using AMPure XP beads (Beckman Coulter), the concentration of the cDNA library was measured with an Agilent 2200 TapeStation using the High Sensitvity DNA 5000 kit (Agilent). 1 ng of cDNA from this reaction was amplified and barcoded using the Nextera XT DNA sample preparation kit and Nextera XT index kit (Illumina) following the manufacturer's protocol. The libraries were purified again using AMPure XP beads, analyzed on the Tapestation 2200 using High Sensitivity DNA 100 Kit and quantified using the Qubit fluorometer and Qubit dsDNA HS Assay Kit (Thermo Fisher Scientific).

For sorted populations the SMARTer® Stranded Total RNA-Seq Kit v2 -Pico Input Mammalian kit (Takara) was used. Input RNA was purified using the PicoPure RNA Isolation Kit and the protocol was followed according to the manufacturer's guidelines.

RNA-Sequencing

All RNA-Sequencing was carried out at Edinburgh Genomics on a NovaSeq SP flowcell generating 50 base pair (bp) or 75 bp paired end reads. LCM-Seq samples were sequenced at a read depth of approximately 29 million reads per sample. Bulk-sorted population samples were sequenced at a read depth of approximately 47 million reads per sample. 10X samples were sequenced on a NovaSeq S1 flow cell with a 26/8/91 cycle set up at a read depth of approximately 70,000 reads per cell.

LCM-Seq and Bulk Population Transcriptome analysis

The quality of the reads including Phred quality score (quality of the identification of nucleobases during sequencing) adaptor contamination, GC content, duplicate levels was assessed using the tool FastQC (Andrews, 2010). Illumina adaptor sequences were trimmed off reads using the tool Flexbar (Dodt et al., 2012). Reads were mapped to the human reference genome hg38 (Ensembl version 85) using STAR (Dobin et al., 2013). SAMtools (Li et al., 2009) was used to sort and index the aligned reads. The multicov function of BEDtools (Quinlan and Hall, 2010) was used to count the read fragments per gene and generate a matrix of reads per gene.

In R, the tool DESeq2 (Love et al., 2014) was used for DGE analysis which provides $\log_2$ fold change of genes between two conditions. Per embryo batch effects were corrected for. The p. value is dependent on the statistical test used within the tool which was

either the Wald test or the likelihood ratio test (LRT) as indicated throughout the results chapter. P values were adjusted for by multiple hypothesis correction with the Benjamini and Hochberg method to produce adjusted p. values (p.adj) (Benjamini and Hochberg, 1995). Genes were considered significant with a p.adj < 0.05. Principle component analyses were carried out on variance stabilizing transformed datasets using in-built R statistical tools and visualized using ggplot2 (Wickham, 2019). Heatmaps were also carried out on variance stabilizing transformed datasets using in-built R statistical tools and visualized using the R package pheatmap. Volcano plots were generated using EnhancedVolcano.

Enriched gene sets were found from differentially expressed genes between two conditions using the GSEA tool from the broad institute (Subramanian et al., 2005). In particular annotated gene sets from the following databases were used; Hallmark gene sets (Liberzon et al., 2015), BioCarta, KEGG (Kanehisa and Goto, 2000), Reactome (Croft et al., 2011) and Gene ontology (GO) (Ashburner et al., 2000; The Gene Ontology Consortium, 2019). Genes were input as a ranked list by Wald statistic and pathways were considered significant with a false discover rate (FDR) of < 0.25.

The String-DB was used to find interactions between proteins from lists of genes (Szklarczyk et al., 2015, 2019). These interactions are both direct (physical) and indirect (functional) interactions. The tool determined interactions between proteins by known interactions from curated databases or that had been experimentally determined, predicted interactions from gene neighborhood, gene fusions and gene co-occurrence databases, and by text mining publications, co-expression and protein homology databases. Interactions were given a confidence score scaled between 0 and 1 which is a combined score for each type of evidence given and is the estimated likelihood that a given interaction is 'biologically meaning, specific and reproducible'. Only high confidence score (0.7) interactions were used. The network matrices generated in the string-db web interface were opened in the Cytoscape software (Shannon et al., 2003) for production of figures and scaling of nodes by the number of interactions associated with it. EnrichmentMap was used to consolidate pathways with a high number of overlapping contributing genes (https://www.baderlab.org/Software/EnrichmentMap).

CellPhoneDB v2.0 (Efremova et al., 2020) was used to find ligand receptor interactions between subpopulations. Results were computed using default settings with 1000 statistical iterations. Significant interactions were considered as having a p.value < 0.05.

Sequencing data is accessible through GEO Series accession number GEO: GSE151877.

10x Single Cell Analysis

Libraries from 10,000 CD34+ AoV cells were prepared using the Single Cell 3′ Reagent Kit v2 (10x Genomics) across two lanes of the Chromium Single Cell Chip (10x Genomics). The libraries were prepared according to the manufacturer's protocol.

The Cell Ranger 2.1.0 (10x Genomics) analysis pipeline was used to process the 10x single cell RNA-Seq output by aligning reads to GRCh38 human transcriptome (Ensembl). The single-cell gene expression data was converted into a loom file using the python tool Loompy (https://github.com/linnarsson-lab/loompy). SCANPY (Wolf et al., 2018) was used for the follow steps 1 – 15. 1) Filtering out cells with less than 200 genes. 2) filtering out genes that were in less than 3 cells. 3) Filtering out cells with a) high percentage mitochondrial genes (> 0.05) and b) a high number of genes (> 3500). 4) Normalizing reads per cell. 5) Logarithmise the data. 6) Regress out the effects of total counts per cell and the percentage mitochondrial genes. 7) Regress out cell cycle effect. 8) Identification of highly variable genes for principal component analysis (PCA) 9) PCA 10) Compute the neighborhood graph (n_neighbors = 8). 11) Embed the neighborhood graph in 2 dimensions using Uniform Manifold Approximation and Projection (UMAP) (McInnes, Healy and Melville, 2018). 12) Clustering the neighborhood graph using Leiden (Traag et al., 2019). 13) Finding marker genes per cluster using a Wilcoxon rank-sum (Mann-Whitney-U) test. 14) Mapping gene expression onto the UMAP embedding. 15) Generation of additional graphical plots. Partition-based graph abstraction (PAGA) (Wolf et al., 2019) was used to make lineage inferences from the neighborhood graph, using the Leiden clustering. scVelo (https://github.com/theislab/scvelo) was used to estimate the direction of RNA Velocity (La Manno et al., 2018) across the UMAP embedding and in turn infer lineage trajectories from the PAGA-inferred cluster relationships.

Sequencing data is accessible through GEO Series accession number GSE151877.

Imaging

Immunofluorescence

SuperFrost Plus (VWR) slides with 7μm cryosections were taken out of −20°C storage and placed immediately in cold 4% PFA (Sigma Aldrich) for 10 min. Staining steps were as follows; 3x wash in PBS, 5 min each. 10 min permeabilisation in PBS/0.5% Triton X-100 (Sigma Aldrich). 2x 5 min PBS wash. 30 min PBS/10% FCS protein block. Overnight incubation with a primary antibody diluted in PBS/2% FCS. 2x 5 min PBS wash. 2-hour incubation with secondary antibody diluted in PBS/2% FCS at room temperature. 2x 5 min PBS wash, 5 min 30nM DAPI (ThermoFisher) incubation. 1x 5 min PBS wash. Mount in Prolong Gold Antifade (ThermoFisher) and coverslip of thickness 1.5 (VWR). Slides were left at room temperature in the dark overnight before imaging. For each staining experiment there was a negative control with no primary antibodies and all secondary antibodies or with an isotype control and all secondary antibodies.

RNAScope

RNAScope was carried out using the RNAscope® Multiplex Fluorescent Reagent Kit v2 (bio-techne) largely following the manufacturer's instructions for use with fresh frozen samples. Differences were an initial 30 min fixation of the 7μm cryosections in 4% PFA at 4°C and 30 min incubation at 37°C following dehydration. Protease IV was applied for 10 min at RT. The fluorophores used were all Opal™ Dyes (Perkin Elmer); Opal 520, Opal 570 and Opal 690. The probes used were all human RNAscope® Probes; Hs-EDN1,

Hs-EDNRA-C2, Hs-EDNRB-C3, Hs-REN-C3 Hs-CDH5-C2. Positive and negative control slides were also stained using positive and negative probes supplied in the kit.

Slides were imaged using a 5 laser Confocal TCS SP8 (Leica) and an Axio Observer Z1 Inverted Microscope (Zeiss). All image analysis was done using ImageJ FIJI software (Schindelin et al., 2012). To quantify RNAScope *EDN1* hotspots (clusters of probe signals) the image threshold was first adjusted to filter out weak and background signals and a binary color image of probe signal versus non-signal was generated. Process → Binary → Watershed was used to help separate merged signals. The analysis tool was then used to highlight any signal clusters that were 300 pixels or larger. Histology images were annotated on PowerPoint (Microsoft).

Ex vivo floating membrane aggregate cultures

E9.5 caudal parts from CD45.2/.2 BL6J mice were dissociated in 1mg/ml Collagenase-Dispase (Roche) and 0.12 mg/ml of DNase I (Roche) for 35 min in a 37°C rotating water bath. FACS buffer (PBS without Ca^{2+} and Mg^{2+}, 2% Foetal Calf Serum (FCS), and 50 U/ml P/S) was added to neutralise the collagenase and cells were centrifuged for 5 min at 300 g. Supernatant was aspirated and tissues mechanically dissociated by gentle pipetting up and down in FACS buffer. Cells were then centrifuged and washed again. Cells were then resuspended in IMDM/20% heat inactivated FCS (Hyclone) with addition of one of the recombinant proteins; Endothelin-1 (LS-Bioscience), Endothelin-2 (Sigma Aldrich), Renin (Sigma Aldrich) at a concentration of 10ng/ml or 100ng/ml. A no-protein control was also included. Dissociated cells were distributed into p200 pipette tips sealed at the ends with paraffin; 1 embryo equivalent (ee) per tube in 20μl. Tips were centrifuged at 460 g for 12 min to form a pellet. For OP9 co-aggregate experiments, cells were centrifuged with 100,000 OP9s per aggregate. The pellets were carefully deposited onto 0.8μm nitrocellulose membrane filters (Millipore) floating on Aggregate Media (control or + 1 recombinant protein at 10ng/ml or 100ng/ml). A maximum of 5 aggregates were cultured per membrane at the gas-liquid interface for 7 days at 37°C, 5% CO_2. After 7 days of E9.5 floating membrane aggregate culture, aggregates were dissociated in 1mg/ml Collagenase-Dispase (Roche) at 37°C for 40 min for down-stream assays.

ES Cell Differentiation

H9 ES cells were differentiated toward hematopoietic progenitors following a previously published protocol (Ng et al., 2016). All incubation was carried out at 37°C and 95% humidity in an atmosphere of 5% CO2. All proteins are human recombinant. Day 0: Cells are dissociated with Accutase and plated in a U-bottom 96 well plate at 3000 cells per well in STEMdiff APEL2 (StemCell Technologies, Inc.) with BMP4 (20 ng/ml, R&D), VEGF (25 ng/ml, Peprotech), Activin A (10 ng/ml, Peprotech), FGF2 (10 ng/ml, Peprotech), SCF (25 ng/ml, Peprotech) and ROCK inhibitor (10μM, R&D). Day 2: Addition of SB431542 (3μM, Cayman Chemicals) and Chiron (3μM, Tocris Bioscience). Day 4: Media change; STEMdiff APEL2 (StemCell Technologies, Inc.) with BMP4 (20 ng/ml, Biotechne), VEGF (50 ng/ml, Peprotech), FGF2 (10 ng/ml, Peprotech), SCF (50 ng/ml, Peprotech) and IGF2 (30mg/ml, Peprotech). Day 8: 96-well flat bottom plates were coated with Biolaminin 521 (BioLamina) and EBs were transferred to new wells in new media; STEMdiff APEL2 (StemCell Technologies, Inc.) with SCF (50 ng/ml, Peprotech), VEGF (150 ng/ml, Peprotech), FLT3L (25 ng/ml, Peprotech), TPO (25 ng/ml, Peprotech), IL6 (25 ng/ml, Peprotech), IGF2 (20 ng/ml, Peprotech), FGF2 (10 ng/ml, Peprotech), EPO (3U/ml, Peprotech) and IL3 (50 ng/ml, Peprotech). Day 12: EBs and media were both collected for downstream analysis. Endothelin-1 (100ng/ml Sigma-Aldrich), 1nM ABT-627/atrasentan and 100nM A-192621 (kindly donated by Dr. David Webb) were added separately at day 8 of the protocol. Equal amounts of DMSO were added to each test condition and control (1:10000). Human EBs were dissociated in 1mg/ml Collagenase-Dispase (Roche) at 37°C for 40 min for downstream assays.

Colony-forming assays

For mouse experiments, following culture single cells were plated on MethoCult GF M3434 (StemCell Technologies, Inc.) at a frequency of 0.05 embryo equivalents per plate. Plates were cultured at 37°C and 95% humidity in an atmosphere of 5% CO2. Colonies were counted and identified on day 7 of culture.

Dissociated human EBs were plated in MethoCult™ (StemCell Technologies, Inc.) at a frequency of 5000 cells per dish. Colonies were identified and counted after 14 days.

Long-term repopulation assay

Collagenase dissociated cells from E9.5 7 day aggregate cultures were washed and resuspended in FACS buffer with heat-inactivated FCS (hyclone). CD45.1/.2 mice were irradiated prior to transplantation with a dose of 9.5 Gy, split in two doses with a 3-hour gap in between and delivered by a sealed Cs source at a rate of 21.6 rad/min. 0.5ee were injected with a 30-gauge syringe needle into the lateral tail veins of pre-irradiated CD45.1/.2 mice with 100,000 bone marrow carrier cells isolated from the femurs of CD45.1/.1 mice. Blood was collected from the tail vein by superficial incision of the tail vein at 8 and 18 weeks post-transplantation to analyze blood chimerism. Rat anti-mouse CD45.1-APC (Invitrogen, 2 μg/ml, Clone: A20) and rat anti-mouse CD45.1-PE (eBioscience, 2 μg/ml, Clone: 104) were used to stain the peripheral blood cells which were analyzed by a NovoCyte Flow Cytometer (Acea Biosciences, Inc). At 18 weeks the mice were culled and hematopoietic tissues taken for multilineage analysis including the spleen, bone marrow, thymus and blood. Bone marrow was flushed from the femur with FACS buffer using a syringe and 30-gauge needle. Thymus and spleen were chopped with dissection scissors into fragments and pipetted up and down to flush out the hematopoietic cells before filtering out the remaining tissue. Red blood cells in the blood were lysed with Red Blood Cell Lysis Buffer (bioRad) for 15 min at room temperature. Cells were then stained as described below.

Flow cytometric analysis of cells

At 20 weeks post-transplantation cells from the hematopoietic organs of recipient mice were processed as described above and analyzed for multilineage repopulation. Cells were dissociated in 1mg/ml Collagenase-Dispase (Roche) at 37°C for 40 min and filtered through a 35μm nylong mesh. Cells were washed in FACS buffer (PBS without Ca^{2+} and Mg^{2+}, 2% Foetal Calf Serum (FCS), and 50 U/ml P/S) and centrifuged for 5 min at 300 g. Cells were stained for 1 hour in CD45_1-V450 (A20, BD Horizon, RRID:AB_1727490), CD45_2-V500 (104, Biolegend, RRID:AB_10897142), b220-APC-cy7 (RA3-6B2, eBioscience, RRID:AB_394335), CD11c-PE-cy7 (N418, BioLegend, RRID:AB_493569), Ter119-FITC (Thermo Fisher, RRID:AB_465311), Gr1-PE (RB6-8C5, Thermo Fisher, RRID:AB_466044), CD335-BV711 (29A1.4, BioLegend, RRID:AB_2563289), CD3e-APC (145-2C11, Thermo Fisher, RRID:AB_11153519), CD4-APC (GK1.5, Thermo Fisher, RRID:AB_11152647), CD8-BV711 (53.67, BioLegend, RRID:AB_11219594). Cells were washed in FACS buffer and 7AAD was added as live/dead marker. Cells were then analyzed on a BD LSR Fortessa (BD). In all cases, FMOs were used to gate negative populations. OneComp and UltraComp beads (both ThermoFisher) were stained with single antibodies for automatic compensation by the Fortessa. All data was analyzed on FLOWJo software (BD).

QUANTIFICATION AND STATISTICAL ANALYSIS

All statistical analyses except those incorporated into transcriptome analyses were carried out using R (https://www.r-project.org/). Details of the statistical test used and n values where "n" represents the number of experiments and "N" represents the number of embryos are provided in the figure legends. Measure of significance i.e., p value is also provided in the figure legends. Data were analyzed using t test and paired t test for pairwise comparisons of means following verification of normal distribution with Shapiro-Wilk test. For linear correlations a Pearson correlation test was used.

Cell Stem Cell, Volume *27*

Supplemental Information

Multi-layered Spatial Transcriptomics Identify Secretory Factors Promoting Human Hematopoietic Stem Cell Development

Edie I. Crosse, Sabrina Gordon-Keylock, Stanislav Rybtsov, Anahi Binagui-Casas, Hannah Felchle, Nneka C. Nnadi, Kristina Kirschner, Tamir Chandra, Sara Tamagno, David J. Webb, Fiona Rossi, Richard A. Anderson, and Alexander Medvinsky

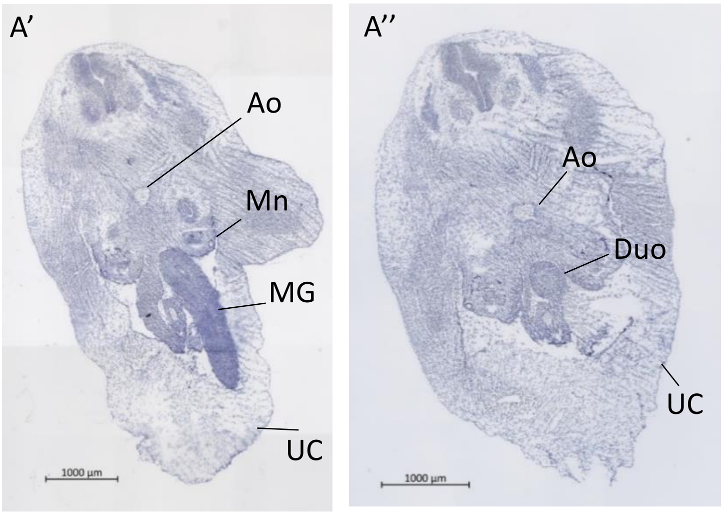

B

	[V] vs [VL]+[DL]+[D]	[VL] vs [V]+[DL]+[D]	[DL] vs [V]+[VL]+[D]	[D] vs [V]+[VL]+[DL]
**LCM-Seq1** No. of genes	+33	+28	+116	+267
	-100	-7	-911	-7
	V_Inner vs D_Inner	V_Mid vs D_Mid	V_Outer vs D_Outer	
**LCM-Seq2** No. of genes	+52	+129	+17	
	-53	-100	-47	

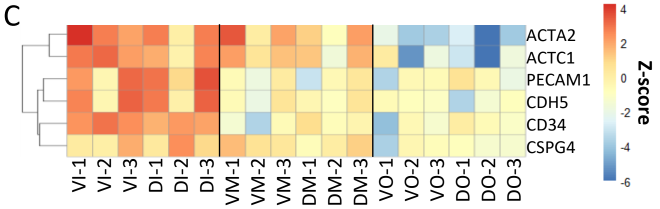

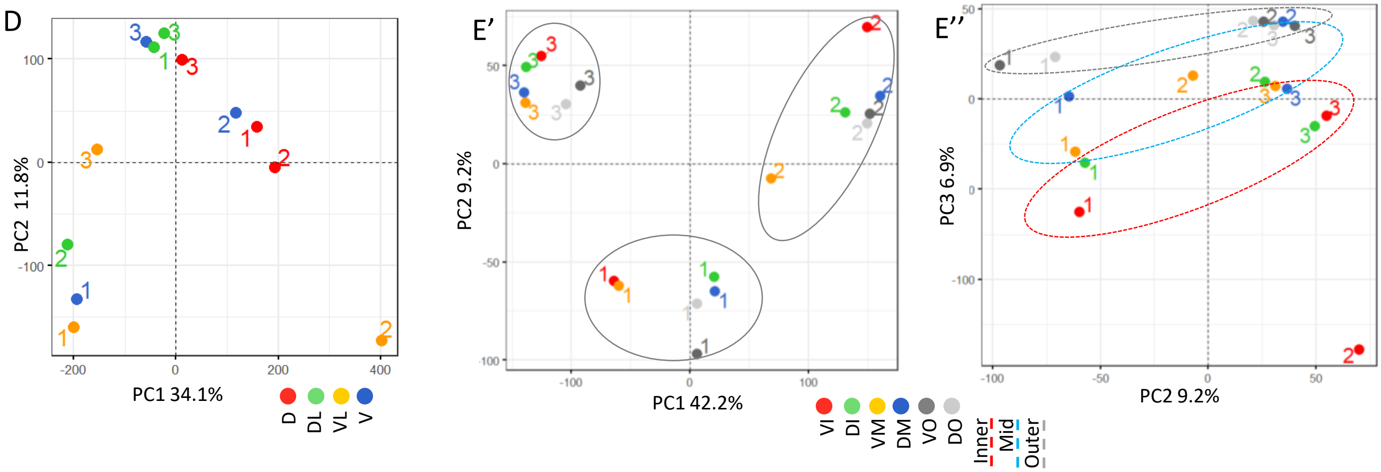

Figure S1, related to Figure 1.

(A) Representative images of anatomical landmarks used for identification of the region to take for LCM-Seq from CS16 transverse sections. A’ shows the midgut loop (MG) protruding into the umbilical cord (UC), Ao= Dorsal Aorta, Mn = Mesonephros. A’’ shows appearance of the duodenum (Duo) and disappearance of the MG.

(B) Table of differentially expressed genes in LCMSeq1 and LCMSeq2 analyses (+ = upregulated, - = downregulated, p.adj<0.05).

(C) Heatmap of ln-normalised gene expression for key endothelial and mesenchymal markers (LCM-Seq2).

(D) PCA analysis of LCM-Seq1 showing PC1 vs PC2. V=Ventral, VL=Ventro-lateral, DL=Dorsal=lateral, D=Dorsal

(E) PCA analysis of LCM-Seq2 showing PC1 vs PC2 (E’) and PC2 vs PC3 (E’’). V/DI = Ventral/Dorsal Inner, V/DM = Ventral/Dorsal Mid, V/DO = Ventral/Dorsal Outer, N=3 embryos.

A

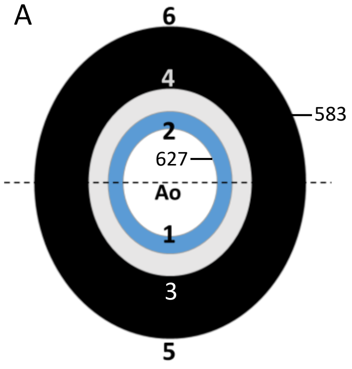

	NES	FDR
Reactome Defensins	-1.99	0.02
Spermatogenesis	-1.45	0.05
Nucleotide-like Purinergic Receptors	-1.73	0.07
Smooth Muscle Contraction	2.75	0.00
Integrin B3 Pathway	2.26	0.00
Epithelial Mesenchymal Transition	2.32	0.00
TGF Beta Signaling	2.31	0.00
TNFA Signaling Via Nfkb	2.23	0.00
Androgen Response	2.14	0.00
Hypoxia	2.08	0.00
IL2 Stat5 Signaling	1.94	0.00
MTORC1 Signaling	1.88	0.00
Myogenesis	1.82	0.00
Coagulation	1.76	0.00
Rho GTPases Activate PAKS	2.20	0.00
Wnt Beta Catenin Signaling	1.76	0.00
Unfolded Protein Response	1.78	0.00
PDGFRb Pathway	2.17	0.00
Cell Extracellular Matrix Interactions	2.18	0.00
Response To Elevated Platelet Cytosolic Ca2plus	2.19	0.00
Nectin Pathway	2.17	0.00
Apoptosis	1.72	0.00
Reactive Oxygen Species Pathway	1.71	0.00
Lymph Angiogenesis Pathway	2.12	0.00

B

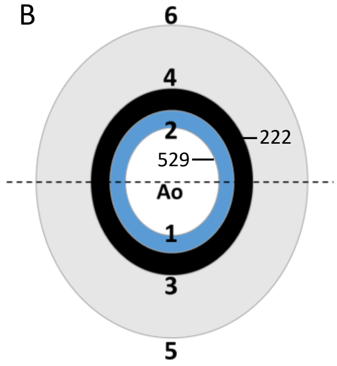

	NES	FDR
DNA Methylation	-2.13	0.00
Mitosis	-2.00	0.00
Transcription	-1.99	0.01
TGF Beta Signaling	1.71	0.03
Nectin Pathway	2.00	0.05
IL23 Pathway	1.89	0.06
Maturity Onset Diabetes of the Young	1.86	0.06
Integrin A9b1 Pathway	1.84	0.07
Integrin Cell Surface Interactions	1.77	0.07
IL2 Stat5 Signaling	1.57	0.07
Leukocyte Transendothelial Migration	1.77	0.07
Platelet Activation Signaling And Aggregation	1.82	0.08
Gap Junction Assembly	1.75	0.09
Notch4 Intracellular Domain Regulates Transcription	1.73	0.09
Pre Notch Processing In Golgi	1.73	0.09
Integrin3 Pathway	1.71	0.10
Signaling By Ras Mutants	1.65	0.14
Oncogenic Mapk Signaling	1.62	0.16
Response To Elevated Platelet Cytosolic Ca2plus	1.59	0.18
Erbb4 Pathway	1.57	0.19
Basigin Interactions	1.54	0.19
Thrombin Par4 Pathway	1.55	0.19
Il8 Cxcr2 Pathway	1.55	0.19

C

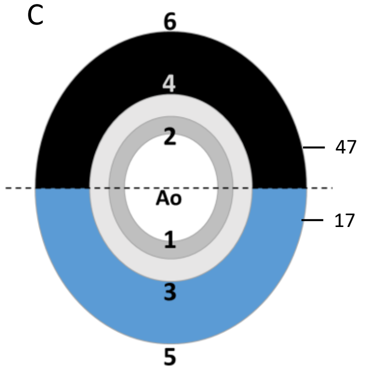

	NES	FDR
E2F Targets	-2.66	0.00
Myc Targets V1	-2.60	0.00
DNA Replication/Mitosis pathways	-2.42	0.00
Oxidative Phosphorylation	-2.15	0.00
Regulation of mRNA Stability By Proteins That Bind AU Rich Elements	-2.28	0.00
Fatty Acid Metabolism	-2.00	0.00
Ribosome	-2.17	0.00
Gluconeogenesis	-2.17	0.00
Mitochondrial Translation	-2.14	0.00
Regulation Of Runx3 Expression And Activity	-2.10	0.00
PDGFRA Pathway	2.15	0.02
EPO Pathway	2.16	0.02
IGF1 Pathway	1.97	0.07
NFAT TF pathway	1.98	0.08
Receptor Type Tyrosine Protein Phosphatases	1.99	0.09
Notch HLH Transcription Pathway	1.93	0.10
PDGF Pathway	1.90	0.11
TPO Pathway	1.89	0.11
Nucleotide Like Purinergic Receptors	1.82	0.13
CD8 TCR Downstream	1.85	0.14
EGF Pathway	1.84	0.14
Renin Angiotensin System	1.84	0.14

D

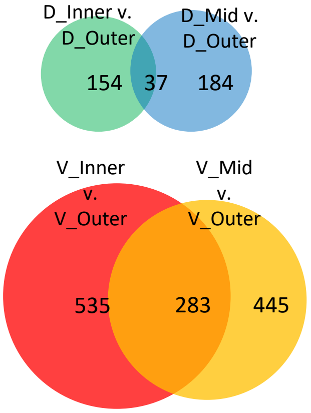

E

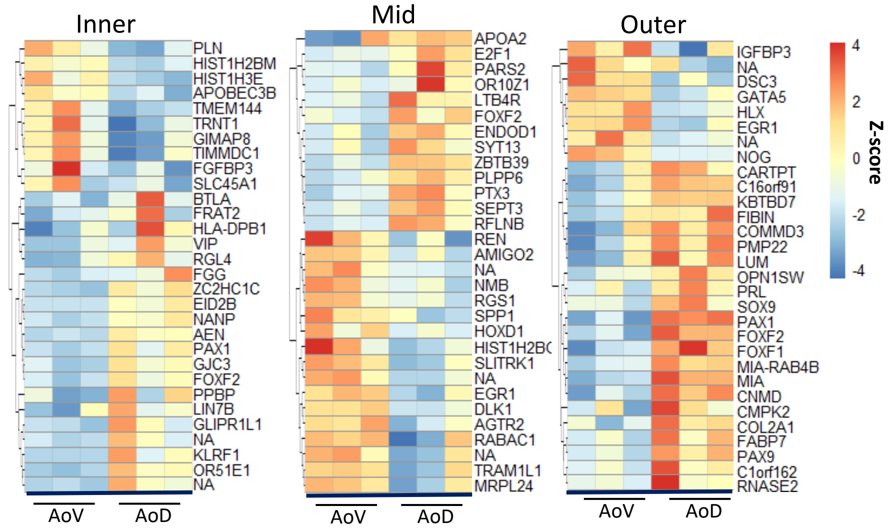

Figure S2, related to Figure 1.

(A) Selected pathways enriched in both V_Inner vs V_Outer and D_Inner vs D_Outer comparisons, FDR<0.25,

(B) Selected pathways enriched in both V_Inner vs V_Mid and D_Inner vs D_Mid comparisons, FDR<0.25. ((A-C) Numbers on schematics show the numbers of genes differentially upregulated in specific sub-domain, p.adj<0.05)

(C) Top differentially enriched pathways ranked by FDR for V_Outer vs D_Outer, FDR<0.25

(D) Number of overlapping pathways (FDR<0.25) between Inner vs Outer and Mid vs Outer for the dorsal (top) and ventral (bottom).

(E) Top 30 genes by significance for ventral vs. dorsal contrast in each concentric layer (p.adj<0.05, NA = Ensembl ID has no assigned gene name).

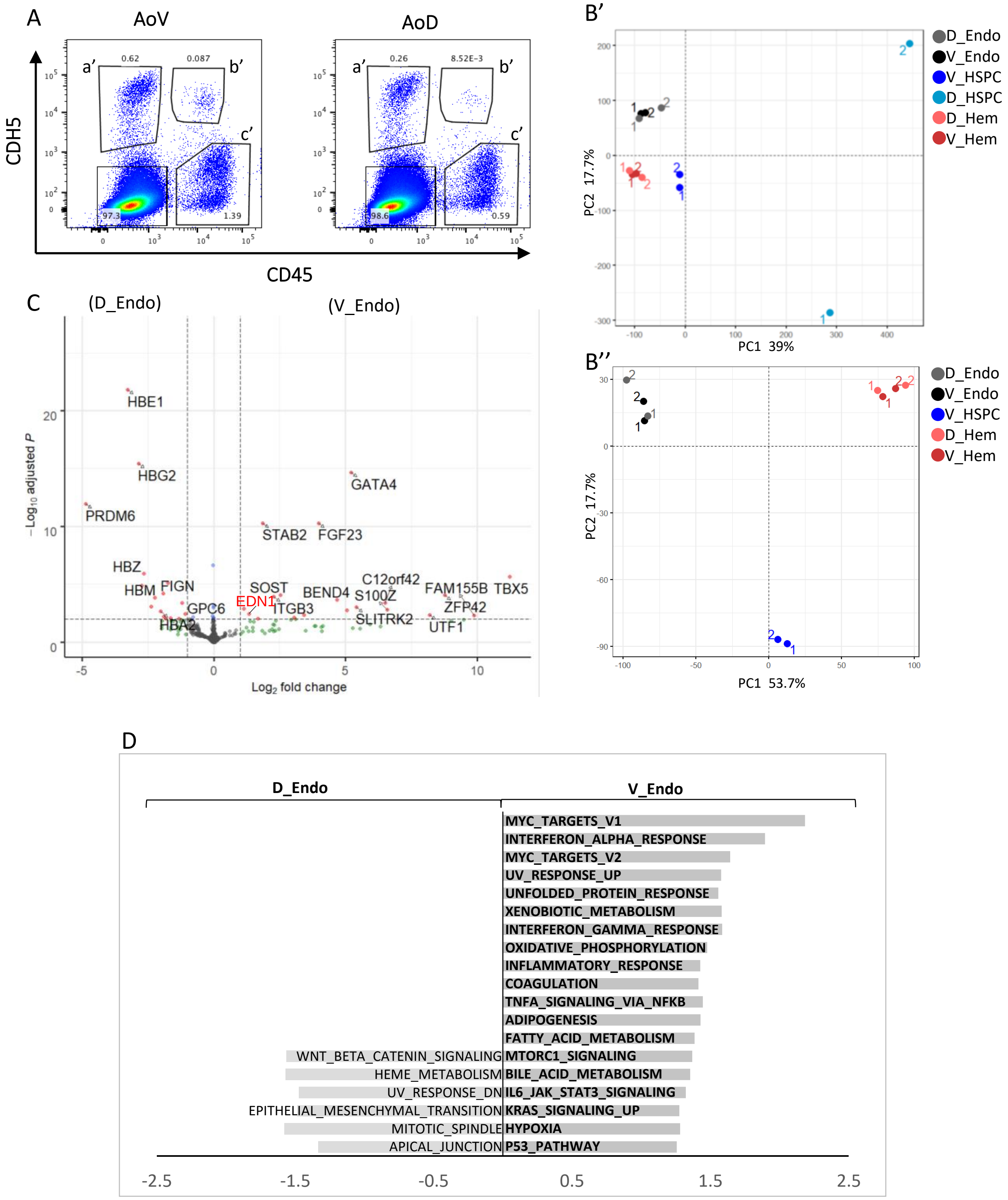
A
AoV
AoD
CDH5
CD45
a'
b'
c'
0.62
0.087
97.3
1.39
0.26
8.52E-3
98.6
0.59
B'
PC1 39%
PC2 17.7%
D_Endo
V_Endo
V_HSPC
D_HSPC
D_Hem
V_Hem
B''
PC1 53.7%
PC2 17.7%
D_Endo
V_Endo
V_HSPC
D_Hem
V_Hem
C
(D_Endo)
(V_Endo)
HBE1
HBG2
PRDM6
HBZ
HBM
PIGN
GPC6
HBA2
EDN1
SOST
ITGB3
BEND4
GATA4
STAB2
FGF23
C12orf42
S100Z
SLITRK2
FAM155B
TBX5
ZFP42
UTF1
Log2 fold change
−Log10 adjusted P
D
D_Endo
V_Endo
MYC_TARGETS_V1
INTERFERON_ALPHA_RESPONSE
MYC_TARGETS_V2
UV_RESPONSE_UP
UNFOLDED_PROTEIN_RESPONSE
XENOBIOTIC_METABOLISM
INTERFERON_GAMMA_RESPONSE
OXIDATIVE_PHOSPHORYLATION
INFLAMMATORY_RESPONSE
COAGULATION
TNFA_SIGNALING_VIA_NFKB
ADIPOGENESIS
FATTY_ACID_METABOLISM
WNT_BETA_CATENIN_SIGNALING
MTORC1_SIGNALING
HEME_METABOLISM
BILE_ACID_METABOLISM
UV_RESPONSE_DN
IL6_JAK_STAT3_SIGNALING
EPITHELIAL_MESENCHYMAL_TRANSITION
KRAS_SIGNALING_UP
MITOTIC_SPINDLE
HYPOXIA
APICAL_JUNCTION
P53_PATHWAY
-2.5
-1.5
-0.5
0.5
1.5
2.5

Figure S3, related to Figure 3.

(A) Sorting strategy for hematoendothelial populations. The dorsal aorta (Ao) was manually bisected into ventral (AoV) and dorsal (AoD) and sorted based on VC and CD45 expression that gave V/D_Endo (a'), V/D_HSPC (b'), V/D_Hem (c') populations.

(B) PCA of haematoendothelial populations including D_HSPC population (B') and after exclusion from further consideration (B''). 1 and 2 indicate independent embryos.

(C) Volcano plot of differentially expressed genes between V_Endo and D_Endo populations (positive and negative loading respectively).

(D) All GSEA pathways for V_Endo vs D_Endo populations.

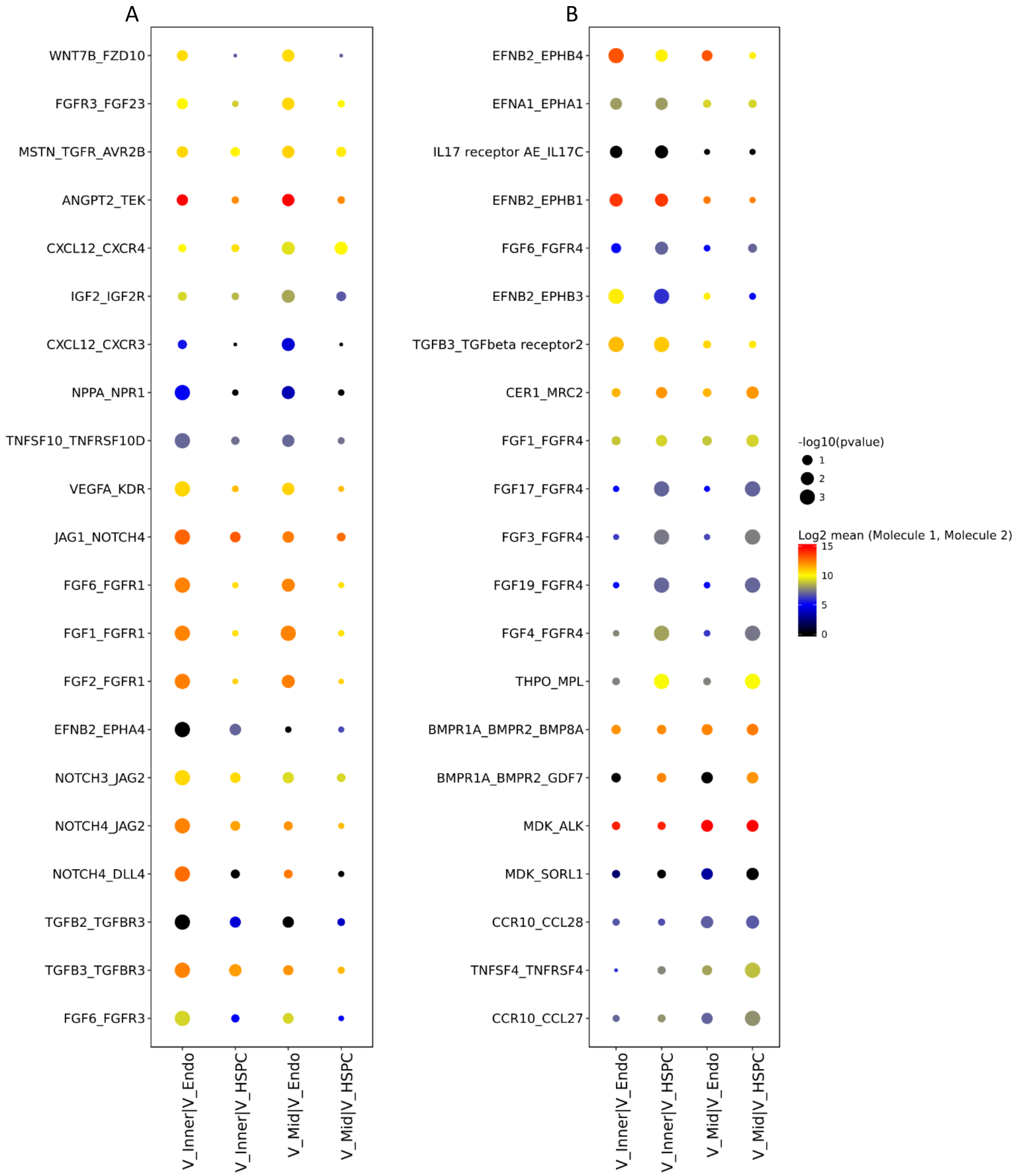

Figure S4, related to Figure 3. CellPhoneDB v2.0 ligand receptor interactions for V_Inner and V_Mid subdomains with V_Endo and V_HSPC populations. Size of spot indicates significance (-log10(pvalue)). Colour indicates expression levels (Log2 mean (Molecule 1 – Molecule 2).

(A) Top interactions by p.value for V_Inner/Mid vs V_Endo.

(B) (B) Top interactions by p.value for V_Inner/Mid vs V_HSPC. p<0.01

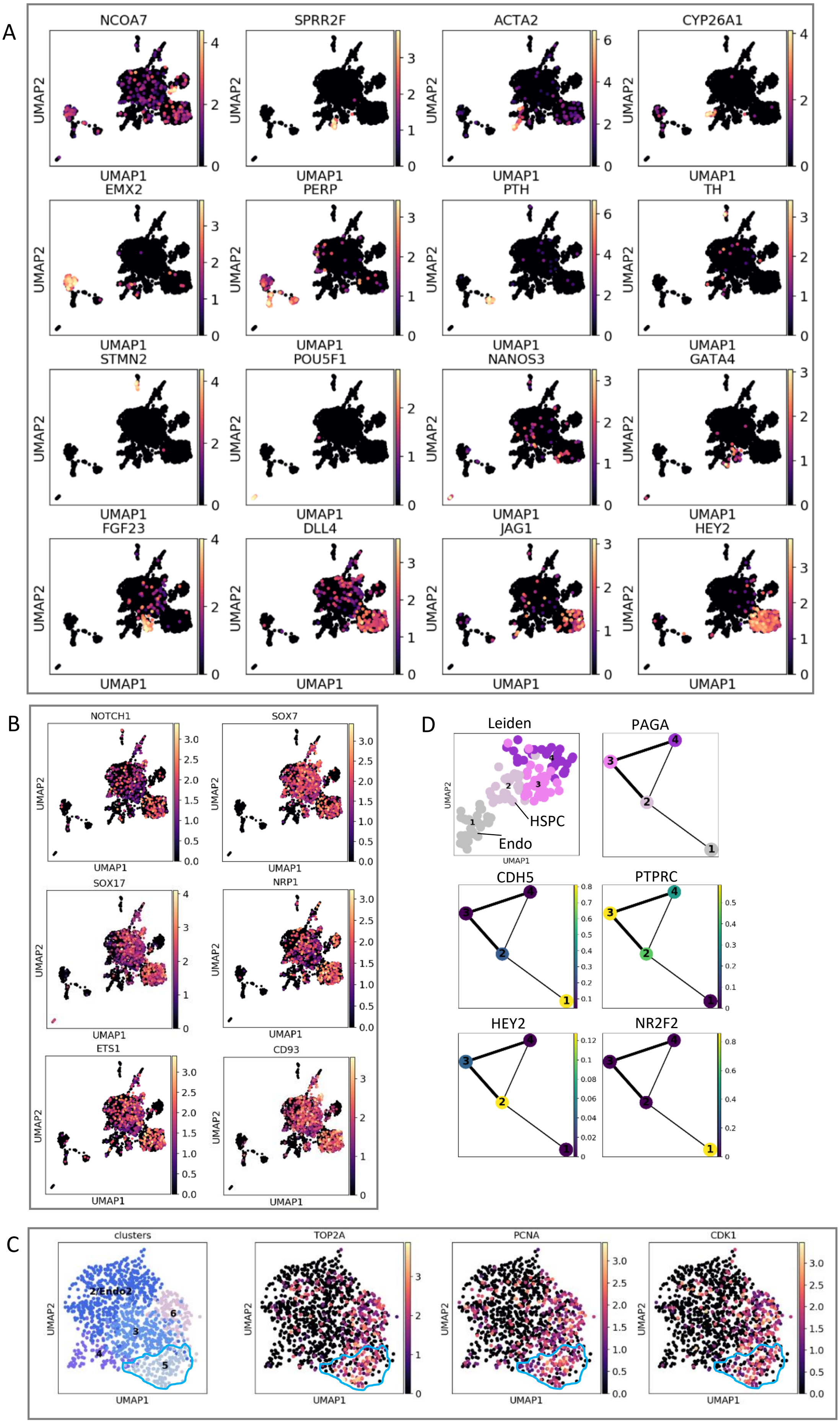
A
NCOA7
SPRR2F
ACTA2
CYP26A1
EMX2
PERP
PTH
TH
STMN2
POU5F1
NANOS3
GATA4
FGF23
DLL4
JAG1
HEY2
UMAP1
UMAP2
B
NOTCH1
SOX7
SOX17
NRP1
ETS1
CD93
D
Leiden
PAGA
HSPC
Endo
CDH5
PTPRC
HEY2
NR2F2
C
clusters
2/Endo2
TOP2A
PCNA
CDK1

Figure S5, related to Figure 4.

(A) Expression mapping of ln-normalised top marker genes to CS16 AoV single cell dataset UMAP.

(B) Expression mapping of additional ln-normalised arterial markers to CS16 AoV single cell dataset UMAP.

(C) Subsetted central endothelial network clusters from AoV dataset with mapped ln-normalised expression of proliferation associated genes. The blue line outlines cluster 5.

(D) Second CS16 single cell dataset of Ao endothelial and HSPCs (N=1). Lineage relationships predicted by PAGA with expression levels of key genes indicated for each cluster.

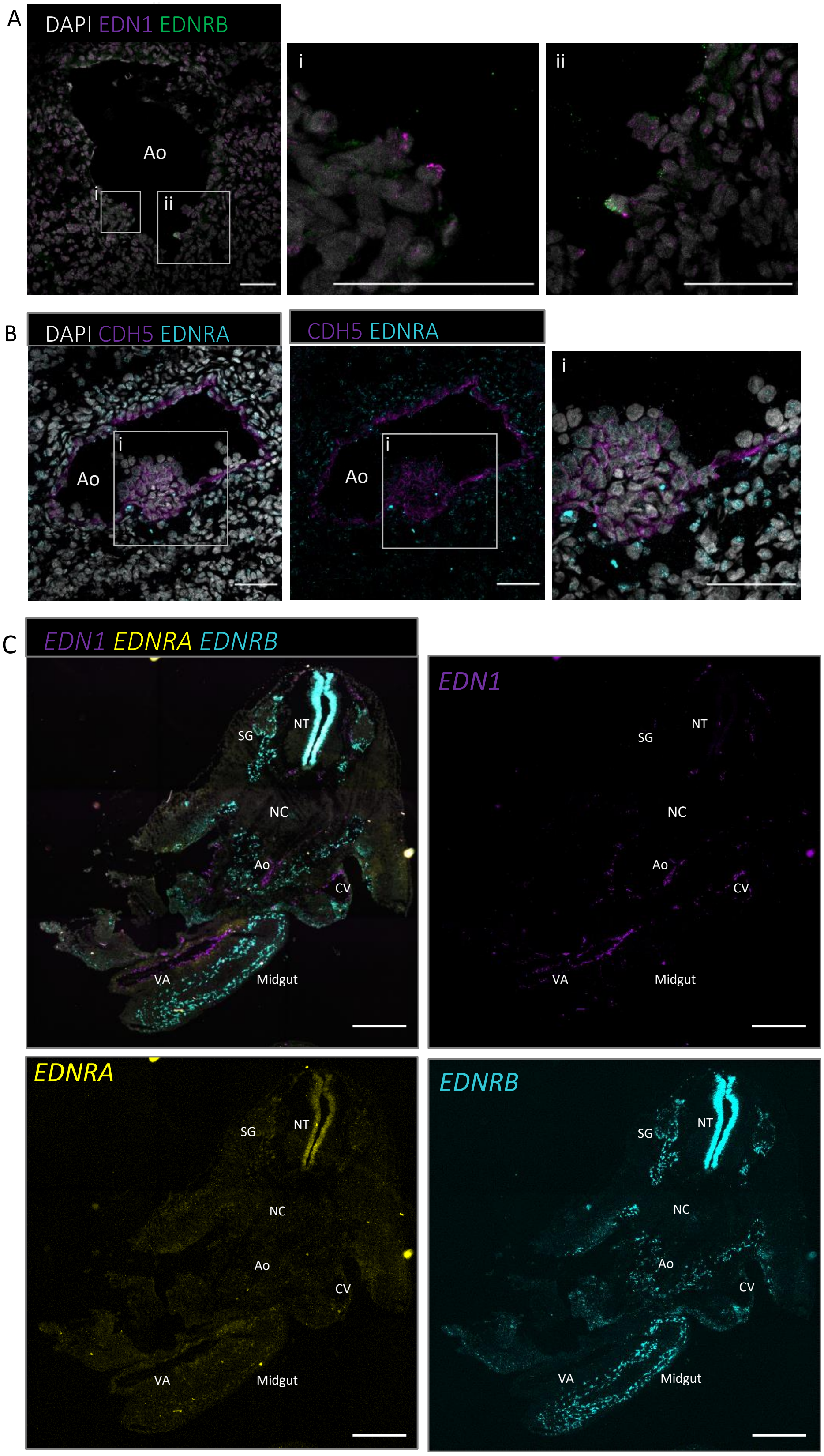

A
DAPI EDN1 EDNRB
Ao
i
ii
B
DAPI CDH5 EDNRA
CDH5 EDNRA
Ao
i
C
EDN1 EDNRA EDNRB
EDN1
EDNRA
EDNRB
NT
SG
NC
Ao
CV
VA
Midgut

Figure S6, related to Figure 6.

(A) EDN1, EDNRB immunostaining (confocal microscopy). Images in i and ii show enlarged area of the boxed region in (A).

(B) CDH5 and EDNRA immunostaining in the dorsal aorta (Ao) and surrounding mesenchyme. Scale bars = 50µm. Image in i show enlarged area of the boxed region in (B).

(A) EDN1, EDNRA and EDNRB mRNA across a CS16 whole tissue section (RNAScope). NT = neural tube, NC = notochord, Ao = dorsal aorta, CV = cardinal vein, VA = vitelline artery. SG = spinal ganglia, scale bars = 500µm.

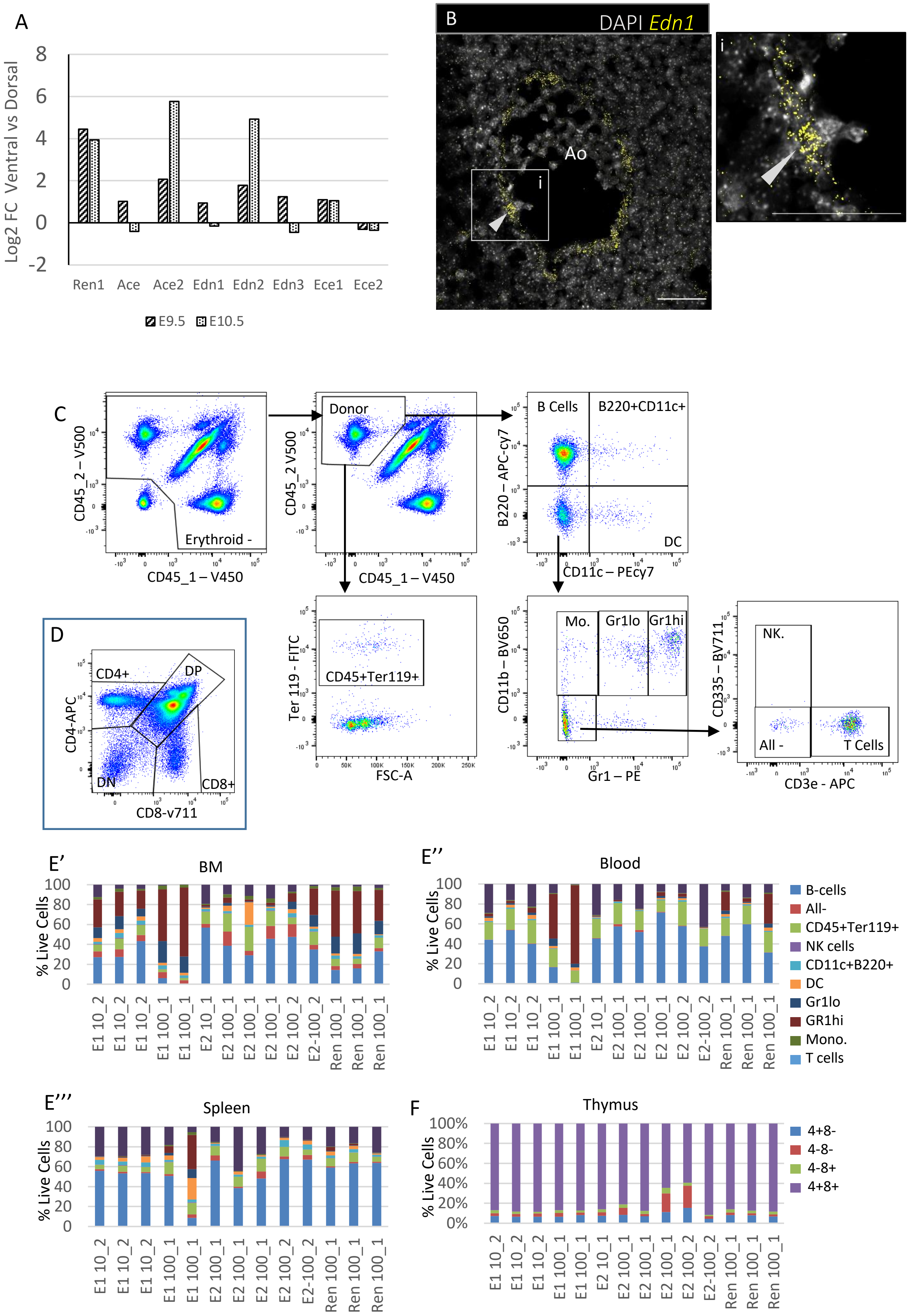

A
Log2 FC Ventral vs Dorsal
Ren1 Ace Ace2 Edn1 Edn2 Edn3 Ece1 Ece2
E9.5 E10.5
B
DAPI Edn1
Ao
i
C
CD45_2 – V500
CD45_1 – V450
Erythroid -
Donor
B Cells
B220+CD11c+
DC
B220 – APC-cy7
CD11c – PEcy7
Ter 119 - FITC
CD45+Ter119+
FSC-A
CD11b – BV650
Mo.
Gr1lo
Gr1hi
Gr1 – PE
CD335 – BV711
NK.
All -
T Cells
CD3e - APC
D
CD4-APC
CD4+
DP
DN
CD8+
CD8-v711
E'
BM
% Live Cells
E''
Blood
B-cells
All-
CD45+Ter119+
NK cells
CD11c+B220+
DC
Gr1lo
GR1hi
Mono.
T cells
E'''
Spleen
F
Thymus
4+8-
4-8-
4-8+
4+8+
E1 10_2
E1 100_1
E2 10_1
E2 100_1
E2 100_2
E2-100_2
Ren 100_1

Figure S7, related to Figure 7.

(A) Log2 fold change of the mouse AoV vs AoD at E9.5 and E10.5. Dataset from A.McGarvey et all., 2017.

(B) *Edn1* mRNA expression in E10.5 dorsal aorta (Ao). Arrowhead shows *Edn1* hotspot directly below IAHC.

(C) Representative multilineage analysis of harvested recipient's organs from reconstituted recipients shown in Figure 7D;

(D) Representative donor-derived cell contribution into the recipient's thymus of reconstituted recipients.

(E, E', E'', F) Donor-derived cell composition of the bone marrow (BM), blood,spleen and thymus (each population is shown as a percentage of live cells).

Analysis set	Dissection strategy	Subdomain	Shorthand	Description
LCM-Seq1	D-V axis across entire Ao calibre (Figure 1A)	Ventral	[V]	Vessel wall (3-4 cells depth)
		Ventro-lateral	[VL]	
		Dorsal-lateral	[DL]	
		Dorsal	[D]	
LCM-Seq2	Concentric layers radiating from Ao (Figure 2A)	Ventral Inner	V_Inner	Endothelial/Perivascular
		Dorsal Inner	D_Inner	Endothelial/Perivascular
		Ventral Mid	V_Mid	Sub-aortic stroma
		Dorsal Mid	D_Mid	Sub-aortic stroma
		Ventral Outer	V_Outer	Outer stroma
		Dorsal Outer	D_Outer	Outer stroma

Table S1, related to Figures 1 and 2. Nomenclature for sub-domains microdissected for LCM-Seq1 and LCM-Seq2 analyses.

Analysis set	Dorsal/Ventral	Population	Shorthand	Description
Sorted haematoendothelial populations from AoV and AoD (RNAseq)	Ventral	VC+CD45-	V_Endo	Endothelial
	Ventral	VC+CD45+	V_HSPC	Haematopoietic Stem Progenitor Cells
	Ventral	VC-CD45+	V_Hem	Mature haematopoietic
	Dorsal	VC+CD45-	D_Endo	Endothelial
	Dorsal	VC+CD45+	D_HSPC	Haematopoietic Stem Progenitor Cells
	Dorsal	VC-CD45+	D_Hem	Mature haematopoietic

Table S2, related to Figure 3. Nomenclature for the sorted HE populations dataset.
